# Targeting aurora kinases limits tumour growth through DNA damage-mediated senescence and blockade of NF-κB impairs this drug-induced senescence

**DOI:** 10.1002/emmm.201201378

**Published:** 2012-11-25

**Authors:** Yan Liu, Oriana E Hawkins, Yingjun Su, Anna E Vilgelm, Tammy Sobolik, Yee-Mon Thu, Sara Kantrow, Ryan C Splittgerber, Sarah Short, Katayoun I Amiri, Jeffery A Ecsedy, Jeffery A Sosman, Mark C Kelley, Ann Richmond

**Affiliations:** 1 Department of Veterans Affairs, Tennessee Valley Healthcare SystemNashville, TN, USA; 2 Department of Cancer Biology, Vanderbilt University Medical CenterNashville, TN, USA; 3 Division of Dermatology, Vanderbilt University Medical CenterNashville, TN, USA; 4 Millennium Pharmaceuticals, Inc.Cambridge, MA, USA; 5 Division of Hematology/Oncology, Department of Medicine, Vanderbilt University Medical CenterNashville, TN, USA; 6 Division of Surgical Oncology, Department of Surgery, Vanderbilt University School of MedicineNashville, TN, USA

**Keywords:** aurora kinase, DNA damage, melanoma, NF-κB, senescence

## Abstract

Oncogene-induced senescence can provide a protective mechanism against tumour progression. However, production of cytokines and growth factors by senescent cells may contribute to tumour development. Thus, it is unclear whether induction of senescence represents a viable therapeutic approach. Here, using a mouse model with orthotopic implantation of metastatic melanoma tumours taken from 19 patients, we observed that targeting aurora kinases with MLN8054/MLN8237 impaired mitosis, induced senescence and markedly blocked proliferation in patient tumour implants. Importantly, when a subset of tumour-bearing mice were monitored for tumour progression after pausing MLN8054 treatment, 50% of the tumours did not progress over a 12-month period. Mechanistic analyses revealed that inhibition of aurora kinases induced polyploidy and the ATM/Chk2 DNA damage response, which mediated senescence and a NF-κB-related, senescence-associated secretory phenotype (SASP). Blockade of IKKβ/NF-κB led to reversal of MLN8237-induced senescence and SASP. Results demonstrate that removal of senescent tumour cells by infiltrating myeloid cells is crucial for inhibition of tumour re-growth. Altogether, these data demonstrate that induction of senescence, coupled with immune surveillance, can limit melanoma growth.

## INTRODUCTION

Cellular senescence was originally believed to be a cell culture artifact that limits proliferation of normal cultured cells after a finite number of divisions (Hayflick, 1965). Recent *in vivo* studies demonstrate that cellular senescence is a physiological process, which may lead to growth arrest in response to diverse forms of endogenous or exogenous stress [reviewed in (Campisi & d'Adda di Fagagna, 2007; Kuilman et al, 2010)]. Senescent cells generally display an enlarged and flattened morphology with increased activity of senescence-associated beta-galactosidase (SA-β-gal). Other features of senescence include high levels of p21/WAF1 and p16/INK4a proteins, the DNA damage response (DDR), as well as the senescence-associated secretory phenotype (SASP) (Campisi, 2011). Altogether, these properties make up the “senescent phenotype.”

Senescence is an important tumour-suppressive mechanism in the early-stages of neoplastic transformation. Since senescent cells undergo extended growth arrest, this process can limit the proliferation of damaged cells and provide a potent barrier to neoplastic transformation (Campisi & d'Adda di Fagagna, 2007). Several lines of evidence support the concept of oncogene-induced senescence (OIS) preventing tumour progression (Bennecke et al, 2010; Braig et al, 2005; Chen et al, 2005; Guerra et al, 2011; Michaloglou et al, 2005). For example: senescence is induced in murine prostate cells with *Pten* loss, resulting in suppression of tumourigenesis (Chen et al, 2005). Another well-studied model is the melanocytic nevus, which is a benign tumour. A large majority of nevi have oncogenic BRAF mutations, but have a low probability of progressing to melanoma. The common characteristics of nevi are their low proliferative rate and increased senescence (Michaloglou et al, 2005).

While senescent cells undergo extended cell cycle arrest, they remain metabolically active and develop SASP after severely damaged DNA accumulates (Coppe et al, 2008; Kuilman et al, 2010). Their secretory profile is composed of several different cytokines and growth factors (Campisi, 2011). Due to the production of specific growth factors, senescent fibroblasts can induce premalignant and malignant epithelial cells to proliferate *in vitro*, potentially contributing to tumour formation in aged organisms (Krtolica et al, 2001; Yang et al, 2006a). Senescent fibroblasts can also promote early tumour growth *in vivo* by secreting matrix metalloproteinase (Liu & Hornsby, 2007). In addition, Jackson et al reported that induction of p53-dependent senescence can impair the response to chemotherapy in breast cancer (Jackson et al, 2012).

Although some cytokines can promote tumour proliferation in certain models, the biological functions of the SASP are complex, as some components such as IL-6 and IL-8 actively participate in the maintenance of cellular senescence (Acosta et al, 2008; Kuilman et al, 2008). The SASP can also stimulate immune cells and has anti-tumourigenic effects (Kang et al, 2011; Xue et al, 2007). In addition, inhibition of NF-κB-induced SASP can bypass senescence and contribute to drug resistance in a mouse lymphoma model (Chien et al, 2011; Jing et al, 2011). Therefore, it remains unclear whether therapy-induced senescence results in tumour promotion or tumour suppression.

Here, we utilized an orthotopic implant model of advanced melanoma to evaluate the effect of aurora kinase inhibitor-induced senescence on tumour growth. We also investigated the role of the IKKβ/NF-κB signalling pathway in drug-induced senescence.

## RESULTS

### Targeting aurora kinases limits growth of orthotopic implants of melanoma tumour in mice

Although a recent study reported overexpression of AURKA and AURKB in human melanoma at the tissue level (Wang et al, 2010), it is possible that the elevated expression of AURKA and AURKB was due to the high proliferative capacity of cancer cells, since AURKs are expressed largely during cell division. To evaluate AURK levels in normal melanocytes and melanoma cell populations at the same point in the cell cycle, we synchronized melanoma cell lines and primary melanocytes by treating them with 100 ng/ml of nocodazole for 16 h, followed by mitotic shake-off, and performed Western blotting to analyse AURKA and AURKB protein levels. We observed that the levels of both AURKA and AURKB were significantly higher in synchronized melanoma cell lines than in synchronized normal melanocytes (Supporting Information Fig S1A). To determine whether the AURKA inhibitor MLN8237 inhibits the activation of AURKA phosphorylation on threonine-288 (T288) in melanoma cells, we treated Hs294T cells with MLN8237 for 3 days and performed Western blot analysis for phospho-AURKA (T-288) or phospho-AURKB (T-232). Results revealed that MLN8237 inhibits the phosphorylation of both AURKA and AURKB, though it is more specific to AURKA (Supporting Information Fig S1B).

**Figure 1:**
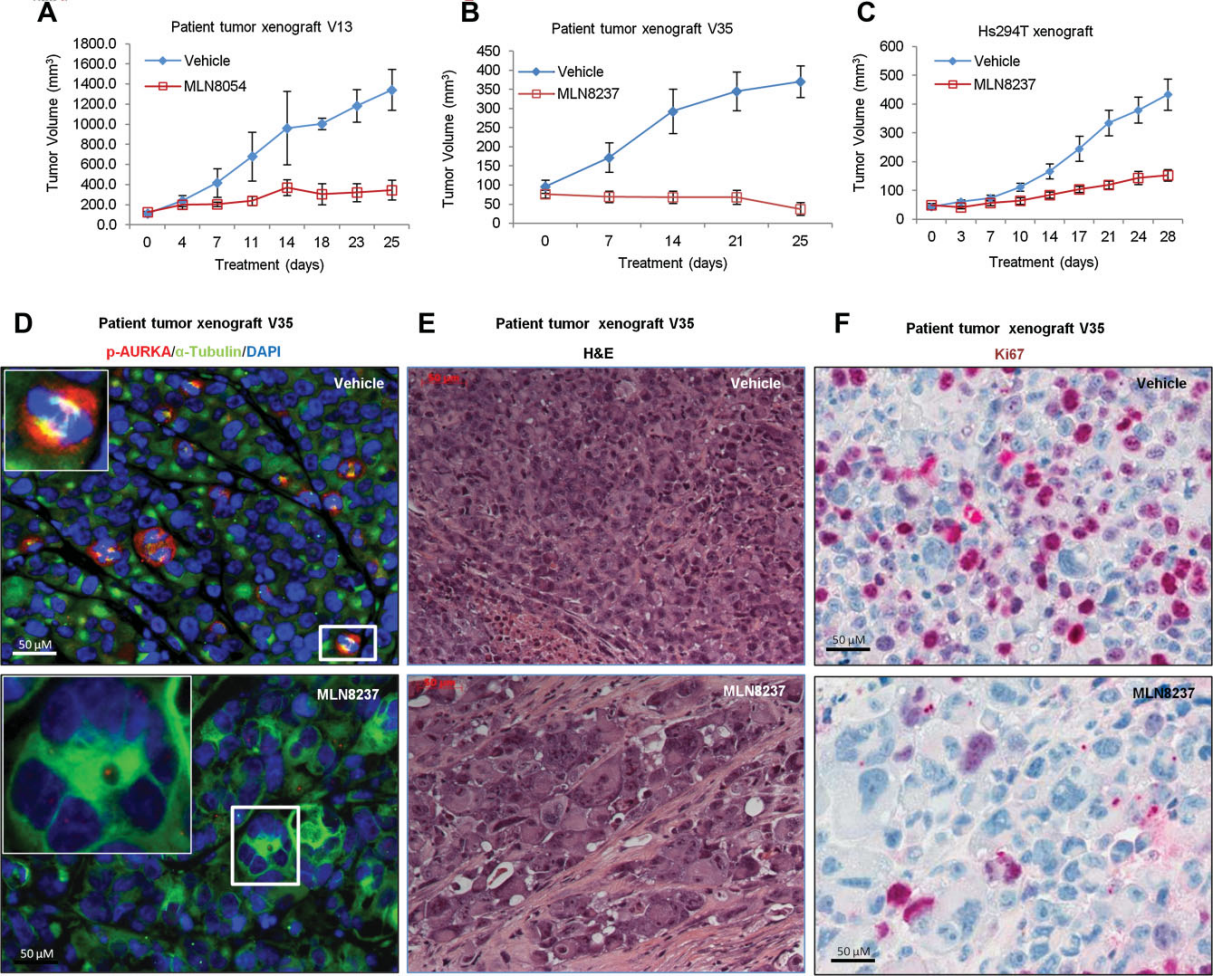
Targeting aurora kinases limits melanoma growth **A,B.** Biopsy tumour tissues from 19 melanoma patients were implanted into nude mice and when tumours formed they were passaged by implantation into treatment groups (*n* ≥ 4 mice per group). Tumour-bearing mice received MLN8054 (60 mg/kg) or vehicle alone (**A**), MLN8237 (30 mg/kg) or vehicle alone (**B**), once daily by oral gavage for 2–6 weeks depending on their response to the treatment or the tumour volume. Mean tumour volumes ± SEM are shown for patient V13 (**A**) or V35 (**B**) as representative patient tumours.**C.** Hs294T melanoma cells were injected subcutaneously into nude mice (2 × 10^6^ cells per mouse). After 1 week, tumour-bearing mice were treated with vehicle control or MLN8237 (30 mg/kg) once daily for 28 days. Tumour volume was then evaluated to determine response to the drug. Mean tumour volumes ± SEM for five mice per treatment group are shown.**D.** Tissue microarray (TMA) slides from patient melanoma tumour implants growing on mice treated with vehicle control or MLN8237 were stained for pAURKA (T-288) (red), alpha tubulin (green) and DAPI (blue) to identify cells with p-AURKA associated with the nuclear spindle microtubule complex (yellow) (see insert enlargement in upper panel).**E.** Histological features of the H&E stained patient melanoma tissues from mice treated with vehicle or MLN8237.**F.** Ki67 (proliferation marker) staining of patient tissues from mice treated with MLN8237 or vehicle control.

To determine whether targeting aurora kinase can inhibit melanoma growth *in vivo*, we implanted surgically resected tumours from melanoma patients into Fox nu/nu mice and then propagated tumours from the 19 patients whose tumour grew in mice by transplantation into additional Fox nu/nu mice. Tumour-bearing mice received oral doses of AURKA inhibitors, MLN8054 (60 mg/kg, QD), MLN8237 (30 mg/kg, QD) or vehicle control once daily. Substantial and significant inhibition of tumour growth was observed in implants from 18 of 19 patients. Representative graphs of the growth response to MLN8054 or MLN8237 are shown in Fig 1A and B. Graphs depicting growth response curves of all other patient tumour implants are presented in Supporting Information Figs S2 and S3. In addition to patient melanoma tissues, we also investigated the effects of MLN8237 on the growth of Hs294T metastatic melanoma cell line xenografts. There was a 70% decrease in tumour volume in MLN8237-treated mice compared to vehicle control-treated mice (Fig 1C).

Histological analysis of the effects of targeting aurora kinase in melanoma tumours was performed on tissue microarrays (TMA). These arrays were constructed for each of the 19 patients, where four separate cores from each tumour grown in each of four mice treated with either vehicle or MLN8054/MLN8237 were used. Two tumours, V23 and V32, were necrotic or highly pigmented, respectively, and were not evaluable in the TMA analysis. To determine whether blockade of aurora kinase impairs mitosis, we analysed nuclei, alpha-tubulin and phosphorylated AURKA (p-T288-AURKA) on the TMA slides by immunofluorescence. In vehicle-treated samples, cells were dividing with the expression of p-AURKA (red) localized around the α-tubulin in centrosomes and bipolar spindles (green) (Fig 1D upper). In contrast, MLN8237-treated samples exhibited cells with non-bipolar or multi-polar spindles without detection of p-AURKA (Fig 1D lower), indicating that MLN8237 inhibited phosphorylation of AURKA, impaired the formation of the bipolar spindle, and blocked mitosis. Supporting Information Fig S4 shows the quantitative analysis of the results for p-AURKA staining on all patient tumours receiving vehicle control or MLN8237/MLN8054 treatment.

H&E staining of TMA slides reveals that cells in the MLN8237/8054-treated tumours, both implanted patient tumours and the Hs294T cell line xenograft exhibited greatly enlarged cellular size and these cells were often multi-nucleated (Fig 1E and Supporting Information Fig S5). When cell proliferation was examined by Ki67 staining, proliferation was reduced in MLN8237/MLN8054-treated tumours compared to vehicle-treated tumours (Fig 1F and Supporting Information Fig S6), suggesting that targeting aurora kinases inhibits cell proliferation.

Since blocking AURK leads to polyploidy, there was concern that treatment with MLN8237 might increase formation of spontaneous tumours in normal tissues of ageing mice. We thus sought to investigate whether MLN8237 treatment can induce spontaneous tumour formation. We treated 12-month-old FVB mice for 4 months with 40 mg/kg MLN8237 daily. No macroscopic tumours were observed in any of the treated or control mice, so organs were fixed, embedded, sectioned, H&E stained and examined for hyperplasia or tumour formation by a veterinary pathologist who was blind to the study groups. Tumours were found in the lungs of only 2/22 MLN8237-treated mice and no spontaneous tumours were observed in the control group (*p* = 0.499, Fisher's Exact Test). Liver hyperplasia was observed in 3/22 treated mice and 1/16 control mice (*p* = 0.625, Fisher's Exact Test), while colon hyperplasia was present in 1/22 drug-treated mice but not in the control group (*p* = 0.99, Fisher's Exact Test) (Supporting Information Table S1). These non-significant *p*-values are not proof that MLN8237 has no effect on spontaneous tumour formation, but suggest that the effect is small, requiring a much larger sample size to detect a potential effect. Our data suggest that secondary tumour formation should be evaluated in the ongoing MLN8237 clinical trials.

To evaluate the persistence of inhibition of melanoma tumour growth after treatment with MLN8054, treatment was suspended in 14 tumour-bearing mice carrying three different patient tumours and tumour growth was monitored. We observed that 7 of 14 tumours did not regrow over a period of more than 12 months, whereas 7 of the tumours relapsed within 1–3 months after drug administration was paused (Table 1). The H&E staining showed that some areas of the relapsed tumour did not display the enlarged cellular size and multi-nucleated characteristics associated with the MLN8054/8237 response (Supporting Information Fig S7).

**Table 1:** Tumour relapse after treatment

Mouse no.	Tumour regrowth-free period	Further tumour passaging	Response to second treatment
V23A1	43 days	V23P3A (*n* = 9)	0/9
V23A2	59 days	N/A	
V23A3	>12 months	N/A	
V23A4	>12 months	N/A	
V23A5	>12 months	N/A	
V24A1	38 days	V24P3A (*n* = 5)	4/5
V24A2	45 days	N/A	
V24A3	>12 months	N/A	
V24A4	52 days	N/A	
V26A1	72 days	N/A	
V26A2	>12 months	N/A	
V26A3	61 days	V26P3A (*n* = 7)	7/7
V26A4	>12 months	N/A	
V26A5	>12 months	N/A	

*Note*: V24A5 died during the treatment. Tumours that did not exhibit additional growth or exhibited a reduction in tumour size compared to the original tumour volume after 2–3 weeks of treatment with MLN8054 were considered responders.

To investigate whether the relapsed tumours would respond to further drug treatment, three of the seven-relapsed tumours were re-implanted into mice and treated with a second regime of MLN8054. If tumours did not exhibit additional growth or exhibited a reduction in tumour size compared to the original tumour volume after 2–3 weeks of re-treatment, they were considered ‘responders’. Results revealed that one tumour did not respond (V23), whereas mice bearing implants from the other two tumours (V24 [4/5], V26 [7/7]) responded to further treatment with MLN8054 (Table 1). Tumours from patient V23 that did not respond to the second round of treatment did not display the enlarged cellular morphology associated with the senescent phenotype (Supporting Information Fig S8A), whereas tumours from patient V26 responded and exhibited the enlarged cellular morphology associated with the senescent phenotype based upon H&E staining (Supporting Information Fig S8B). One of five V24 patient tumours did not respond to an additional round of treatment (Supporting Information Fig S8C), while the other four responded to treatment (Supporting Information Fig S8D). These data suggest that a second round of treatment may be useful for some patients and when tumours respond to the treatment, they display features associated with senescence, *i.e.* an enlarged cellular morphology.

### Inhibition of aurora kinases results in senescence *in vitro* independent of p53

**Figure 2:**
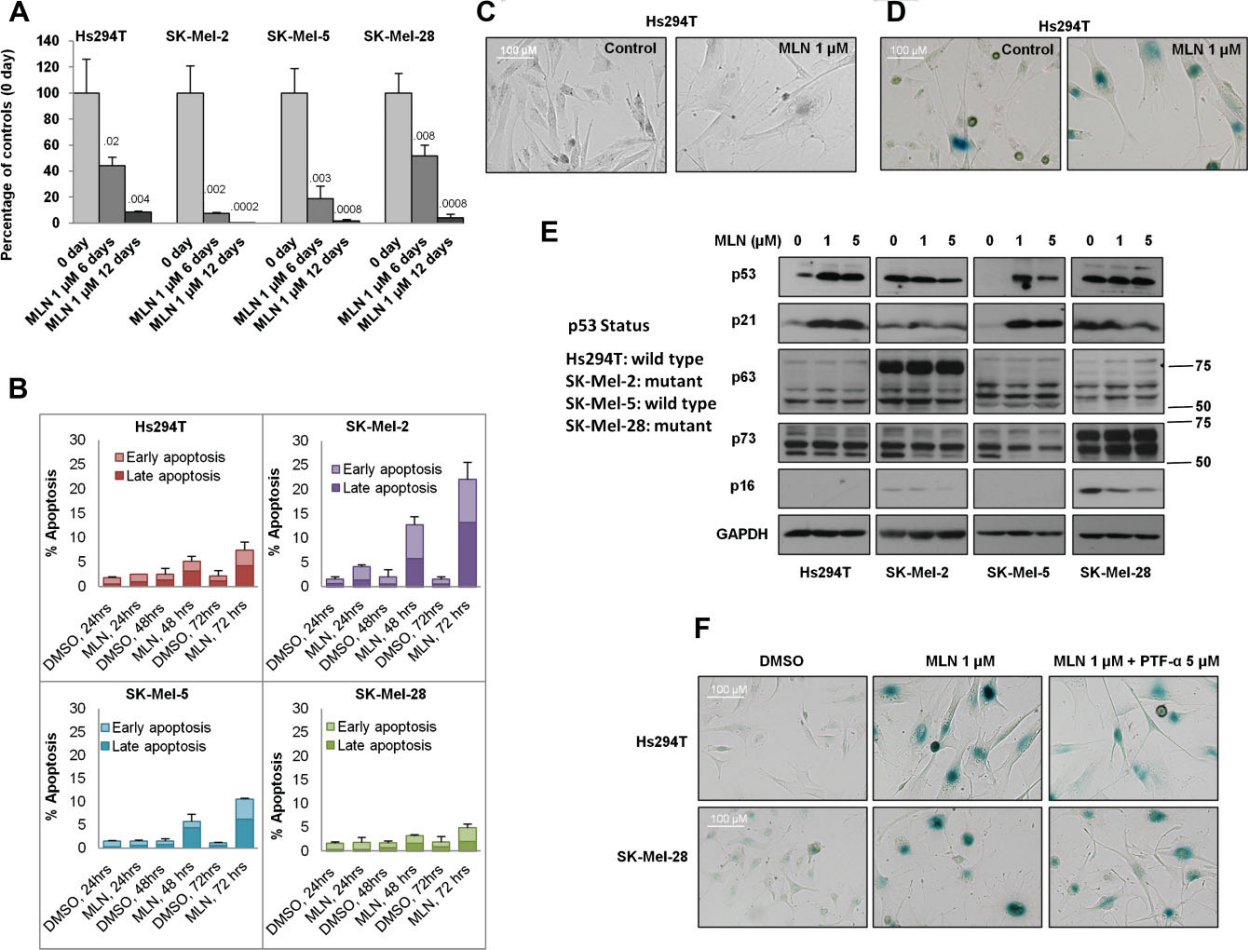
Inhibition of aurora kinases induces cellular senescence *in vitro* independent of p53 Hs294T (p53WT), SK-Mel-2 (p53 mutant), SK-Mel-5 (p53WT) and SK-Mel-28 (p53 mutant) cells were treated with 1 µM MLN8237 for 6 or 12 days. After treatment, viable cells were counted and compared to the initial cell number. Data indicate mean values ± SD (*n* = 3) from one representative of three independent experiments. *p*-value shown represents difference between treated group and day 0 control group (Student's *t*-test).Cultures of Hs294T, SK-Mel-2, SK-Mel-5 and SK-Mel-28 cells were treated with MLN8237 or vehicle for indicated time points and apoptosis was analysed by FACS analysis for propidium iodide (PI) and Annexin V staining. Data indicate mean values ± SD (*n* = 3) from triplicate experiments.Hs294T cells were treated with 1 µM MLN8237 or vehicle for 5 days and the change in morphology was captured with an AxioVision microscope.Hs294T cells were treated with 1 µM MLN8237 for 5 days, and senescence was determined by β-galactosidase staining.Melanoma cell lines with wild-type p53 (Hs294T and SK-Mel-5) or mutated p53 (SK-Mel-2 and SK-Mel-28) were treated with 1 µM or 5 µM MLN8237 for 5 days. After treatment, p53, p63, p73, p21, p16 and GAPDH were analysed by Western blot.Hs294T and SK-Mel-28 cells were treated with 1 µM MLN8237 or vehicle in the presence or absence of the p53 inhibitor pifithrin-α (PTF-α, 5 µM) or DMSO for 5 days. After treatment, β-galactosidase staining was performed. All experiments were conducted at least three times independently with reproducible results.

Although previous reports demonstrated that blocking AURKA/B using small-molecule inhibitors induces widespread apoptosis in different types of human cancer (Dar et al, 2008; Gorgun et al, 2010), we observed little apoptosis in MLN8054/MLN8237-treated melanoma patient tumour implants (Supporting Information Fig S9). *In vitro* studies showed that while the treatment with MLN8237 markedly reduced the number of viable cells (Fig 2A), it induced apoptosis in only 25% of SK-Mel-2 cells and in <10% of cells in three other melanoma cell lines (Fig 2B).

As apoptosis could not account for the significant reduction in cell number in three out of four melanoma cell lines studied or in the tumour implants, we predicted that other processes were responsible for reduced tumour growth in response to drug treatment. Indeed, after 5 days of treatment *in vitro*, we observed that the cellular size was greatly enlarged (Fig 2C), which is a characteristic associated with senescence. The morphological change we observed was consistent with the senescence phenotype described in AURKA- or AURKB-knockdown cells (Kim et al, 2011; Lee et al, 2011). To determine whether the phenotype we observed is caused by senescence, β-galactosidase activity was evaluated and found to be enhanced in drug-treated Hs294T cells (Fig 2D) and in other melanoma cell lines (SK-Mel-2, SK-Mel-5 and SK-Mel-28; Supporting Information Fig S10).

To investigate the mechanism of this therapy-induced senescence, we examined the expression of p53, p63, p73, p21 and p16 in MLN8237-treated cells with either mutated or wild-type p53 status by Western blot. In response to drug treatment, p53 was induced in wild-type (wt) p53 cell lines (Hs294T and SK-Mel-5), but not in mutant p53 cell lines (SK-Mel-2 and SK-Mel-28, Fig 2E). While neither p63 nor p73 was significantly increased in response to the treatment (Fig 2E), p21 was induced in p53 wt Hs294T and SK-Mel-5, but not in p53-mutant SK-Mel-2 and SK-Mel-28 cells. Although p16 is reported to be involved in cellular senescence (Alcorta et al, 1996), it was downregulated in two cell lines (SK-Mel-2 and SK-Mel-28) and was not detected in the other two cell lines (Hs294T and SK-Mel-5, Fig 2E). These results suggest that p53, p21 and p16 are not essential regulators of MLN8237-induced senescence. To further evaluate these findings, we blocked p53 in Hs294T (wild-type p53) and SM-Mel-28 (mutant p53) cells using the p53-specific inhibitor pifithrin-α (PTF-α) (Komarov et al, 1999). Blocking p53 did not alter drug-induced senescence in Hs294T or SK-Mel-28 cells (Fig 2F), indicating that p53 is not required for MLN8237-induced senescence.

### Formation of polyploidy and DNA damage response are induced by MLN8237 treatment

**Figure 3:**
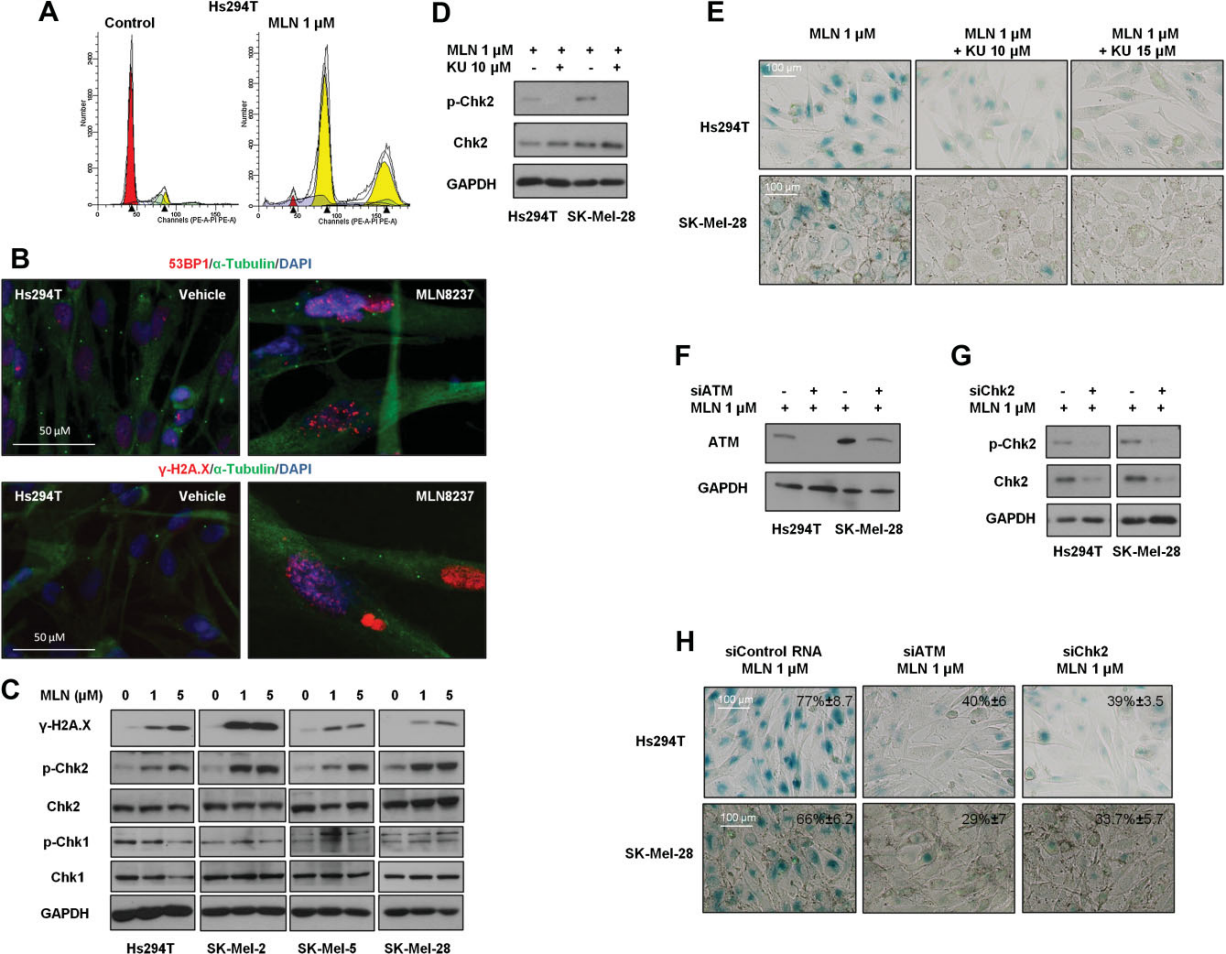
Inhibition of aurora kinases induces polyploidy and DNA damage **A.** Hs294T cells were treated with 1 µM MLN8237 or vehicle control for 2 days and DNA content was examined by FACS.**B.** Hs294T cells were treated with 1 µM MLN8237 or vehicle control for 5 days. After treatment, 53BP1 and γ-H2A.X were evaluated by immunocytochemistry. Cell nuclei were counterstained with Hoechst dye, and the samples were visualized by microscopy.**C.** Hs294T, SK-Mel-2, SK-Mel-5 and SK-Mel-28 cells were treated with vehicle alone, 1 or 5 µM MLN8237 for 5 days, and the levels of γ-H2AX, p-Chk2, Chk2, p-Chk1 and Chk1 were analysed by Western blot.**D,E.** Hs294T cells and SK-Mel-28 cells were treated with 1 µM MLN8237 with or without the ATM inhibitor KU-55933 at the indicated concentrations for 48 h. After treatment, the level of p-Chk2 was examined by Western blot (**D**) and senescence was determined by β-galactosidase staining (**E**).**F–H.** Hs294T cells and SK-Mel-28 cells were transfected with siATM or siChk2. Twenty-four hours after transfection, cells were treated with 1 µM MLN8237 for 3 days. Levels of ATM (**F**), p-Chk2 and Chk2 (**G**) were analysed by Western blot. Senescence after transfection of siATM or siChk2 was evaluated by β-galactosidase staining and representative photomicrographs are shown (**H**). One hundred cells were counted from three independent experiments and mean % senescent cells is noted in each micrograph.

Since aurora kinases play an essential role in cell division (Vader & Lens, 2008), we explored whether treating melanoma cells with an aurora kinase inhibitor would result in aberrant mitosis. Hs294T cells were treated with MLN8237 for 2 days, followed by DNA content analysis by FACS, which revealed this treatment induces polyploidy (Fig 3A). Since polyploidy results in genetic/chromosomal instability (Storchova & Kuffer, 2008; Storchova & Pellman, 2004), we investigated whether MLN8237 treatment induces DNA damage by examining 53BP1 and γ-H2A.X by immunofluorescence. DNA damage in drug-treated but not in control cultures was confirmed by the formation of 53BP1 and γ-H2A.X foci in the nucleus (Fig 3B). To identify which DDR is activated, we examined the levels of p-Chk2 and p-Chk1 in drug-treated cells. Only p-Chk2 was induced in response to the treatment (Fig 3C), indicating that the ATM/Chk2 pathway is activated upon the treatment.

### ATM/Chk2 is required for aurora kinase inhibitor-induced senescence

To investigate whether MLN8237-induced senescence is driven by the ATM/Chk2 pathway, we treated Hs294T and SK-Mel-28 cells with both MLN8237 (1 µM) and an ATM-specific inhibitor KU55933 (10 and 15 µM). KU55933 blocked phosphorylation of the ATM target protein Chk2 (Fig 3D) and impaired senescence in drug-treated melanoma cells (Fig 3E), suggesting that ATM/Chk2 mediates drug-induced senescence. To further confirm our results, we knocked down either ATM (Fig 3F) or Chk2 (Fig 3G) and found that senescence was reduced >30% in knockdown cells (Fig 3H), indicating that the ATM/Chk2 pathway mediates MLN8237-induced senescence.

### Therapy-induced senescence initiates the senescence-associated secretory phenotype (SASP) via NF-κB activation

**Figure 4:**
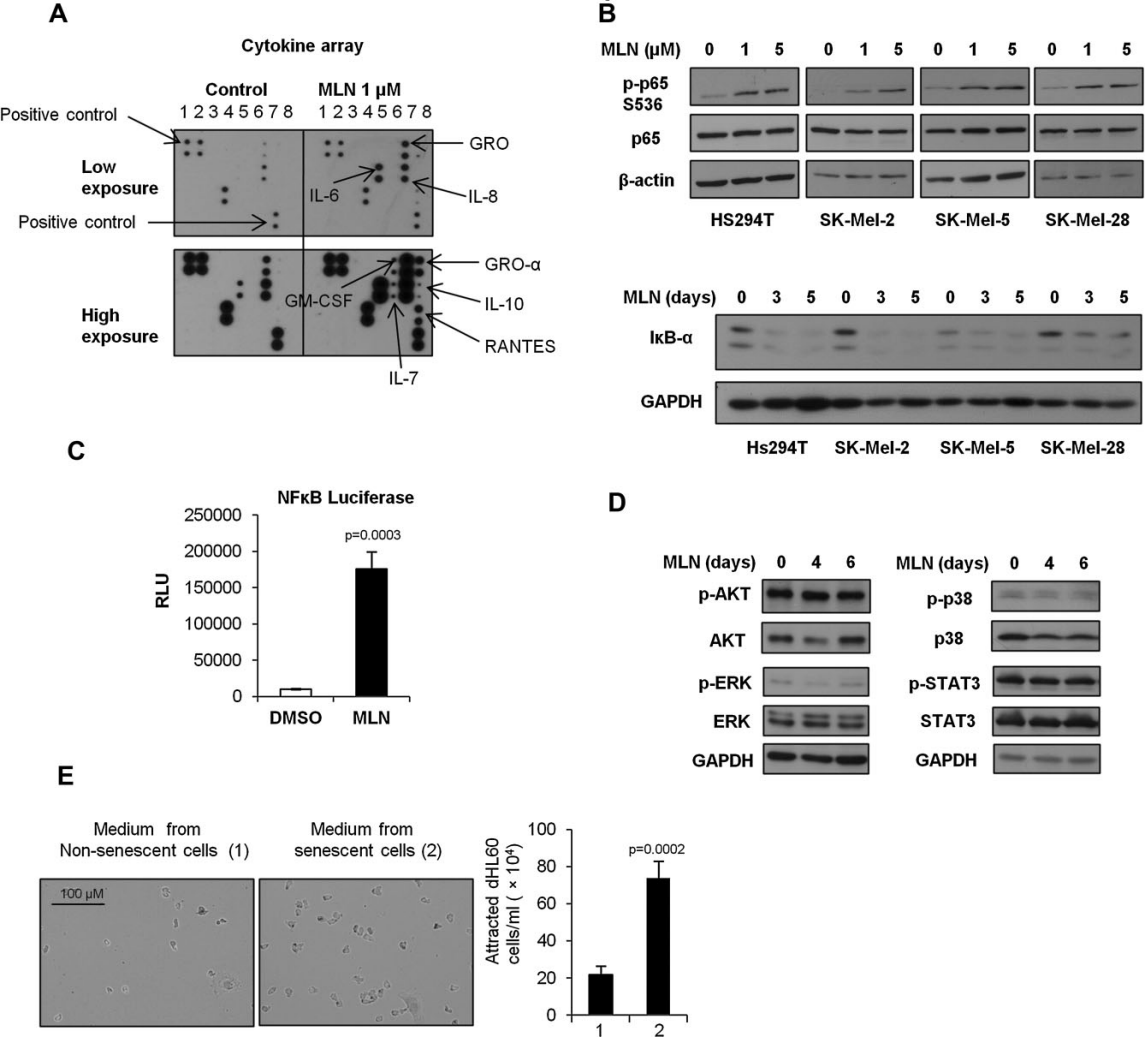
Drug-induced senescence initiates SASP and activates NF-κB Hs294T cells were pre-treated with 1 µM MLN8237 or vehicle control for 5 days. After treatment, cytokine secretion into the medium was assayed by cytokine array.Hs294T, SK-Mel-2, SK-Mel-5 and SK-Mel-28 cells were treated with 1 or 5 µM MLN8237 for 5 days and the levels of p-p65 (S536) and p65 were analysed by Western blot. Hs294T, SK-Mel-2, SK-Mel-5 and SK-Mel-28 cells were treated with 1 µM MLN8237 for 3 days or 5 days, and the level of IκB-α was analysed by Western blot.NF-κB reporter-stable Hs294T cells were treated with 1 µM MLN8237 for 5 days. After treatment, NF-κB transcriptional activity was measured by luciferase assay and the results were normalized by cell number. Data indicate mean values ± SD (*n* = 3) from a representative experiment performed three times.Hs294T cells were treated with 1 or 5 µM MLN8237 for 5 days. Levels of phospho-AKT (p-AKT), total AKT, phospho-ERK (p-ERK), total ERK, phospho-p38 MAPK (p-p38), total p38, phospho-STAT3 (p-STAT3), total STAT3 and GAPDH were measured by Western blot.Conditioned medium from senescent Hs294T cells was added to the bottom wells in 96 transwell cell-plates. 200 µl (10^6^ cells/ml) of dHL60 cells (human promyelocytic leukcmia cells differentiated along a neutrophil cell lineage) were seeded in the chemotaxis chamber. The chamber was incubated at 37°C 5% CO_2_ for 1 h, then the transmigrated cells were counted by haemocytometer. Data indicate mean values ± SD (*n* = 4).

To investigate whether therapy-induced senescence alters the SASP in melanoma cells, we examined the levels of several cytokines and chemokines secreted into the media of MLN8237-treated melanoma cells by cytokine array (Supporting Information Table S2). The results demonstrated that IL-6, IL-7, IL-10, GM-CSF, IL-8, RANTES, GRO and GRO-α were upregulated in response to drug treatment (Fig 4A). We then further tested the levels of IL-6 and IL-8 by ELISA in four melanoma cell lines (Hs294T, SK-Mel-2, SK-Mel-5 and SK-Mel-28) treated with MLN8237 or vehicle and confirmed that both IL-6 (Supporting Information Fig S11A) and IL-8 (Supporting Information Fig S11B) were increased following MLN8237 treatment.

To determine whether the SASP is regulated by induction of NF-κB, we examined the level of phosphorylated NF-κB p65 (p-p65 S536) after MLN8237 treatment by Western blot. Levels of p-p65 were induced and its negative regulator IκB-α was reduced (Fig 4B). An NF-κB luciferase assay was also performed using Hs294T NF-κB reporter cells. NF-κB transcriptional activity was significantly increased after treatment with 1 µM MLN8237 for 5 days (Fig 4C). To rule out the contribution of other signalling pathways to the SASP, we also examined the phosphorylation status of AKT, ERK, p38 MAPK and STAT3 after MLN8237 treatment. Western blot results showed that these pathways were not activated in response to MLN8237 treatment (Fig 4D).

To investigate whether MLN8237-induced SASP results in recruitment of immune cells, we examined the migration of HL60 cells differentiated along the neutrophil lineage in response to conditioned media from MLN8237-treated Hs294T cells. Our data demonstrated that the conditioned media induced migration of dHL60 cells (Fig 4E).

### Targeting aurora kinases leads to senescence, DNA damage response, NF-κB activation and leucocyte recruitment *in vivo*

**Figure 5:**
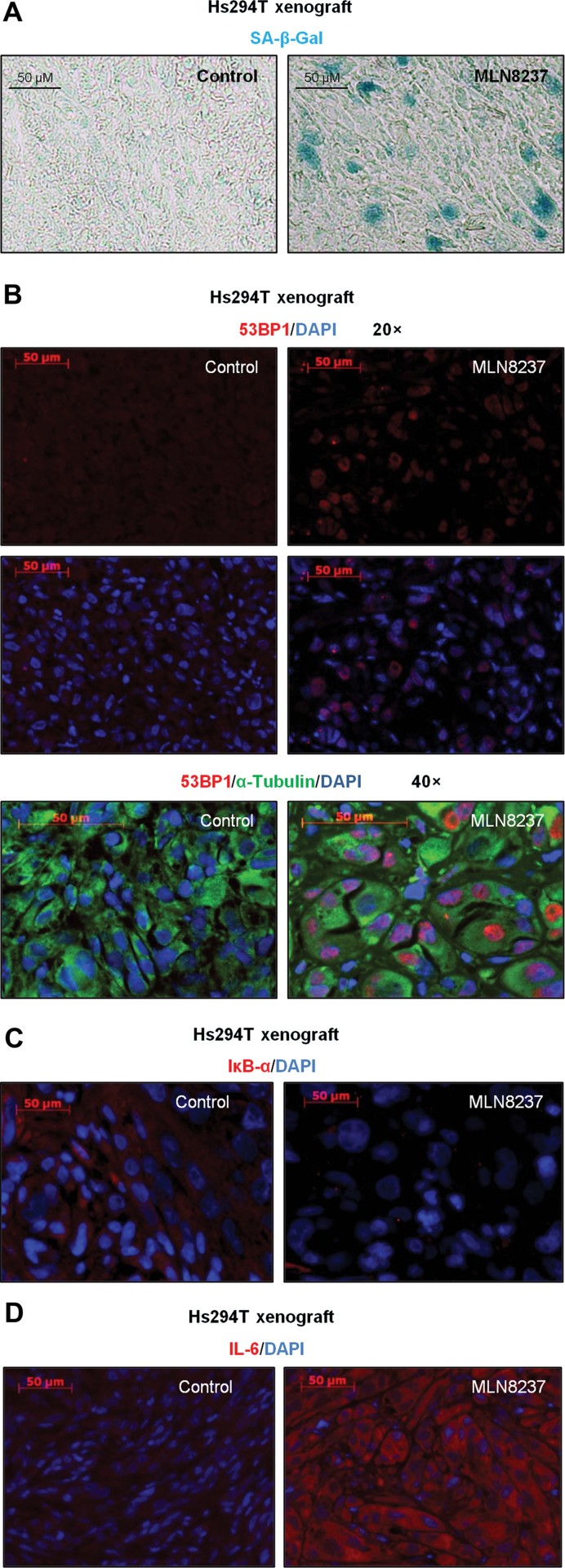
Inhibition of aurora kinases induces senescence, DNA damage and IL-6 *in vivo* **A.** The tissue level of senescence in an Hs294T xenograft treated with MLN8237 or vehicle control was determined by β-galactosidase staining.**B–D.** Tissue levels of 53BP1, α-tubulin, IκB-α and IL-6 in an Hs294T xenograft treated with MLN8237 or vehicle control were visualized by immunofluorescence co-staining with DAPI. Representative micrographs are shown from triplicate experiments.

To extend our findings *in vivo*, we examined senescence (SA-β-Gal staining), DNA damage (53BP1), NF-κB activity (IκB-α) and the SASP (IL-6) in an Hs294T xenograft tumour after MLN 8237 treatment. Tumour tissues treated with MLN8237 were β-galactosidase-positive (blue) (Fig 5A), 53BP1 (red) was increased (Fig 5B, full images are in Supporting Information Fig S12), IκB-α (red) was decreased (Fig 5C) and IL-6 (red) was increased (Fig 5D).

**Figure 6:**
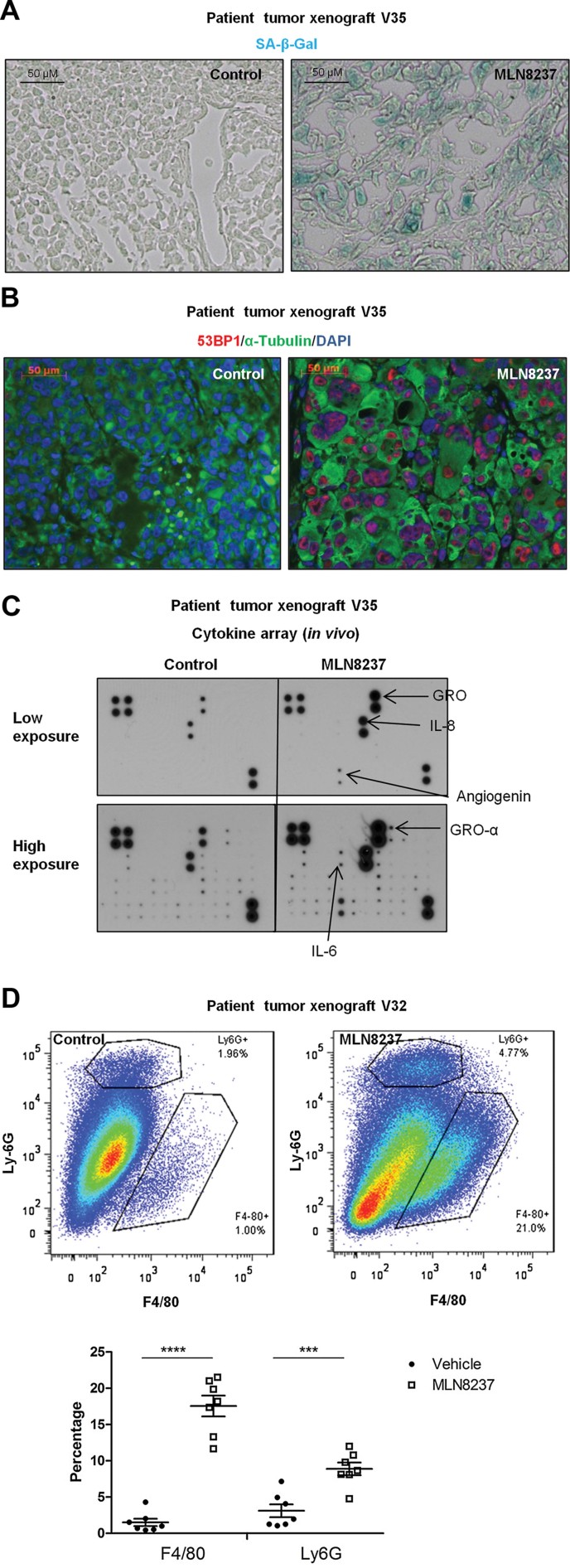
Inhibition of aurora kinases induces senescence, DNA damage and SASP in patient tumour implants treated with MLN8237 The tissue level of senescence in a melanoma patient implant (V35) after mice were treated with MLN8237 or vehicle was determined by β-galactosidase staining.To assess DNA damage, 53BP1 (red) was visualized by immunofluorescence and tissues were co-stained with α-tubulin (green) and DAPI (blue). A and B were analysed from multiple slides and representative images are shown.Cytokine profile of MLN8237-treated tumour tissue was analysed in tumour tissue lysates using cytokine array.The infiltrating neutrophils and macrophages were evaluated by FACS using anti-Ly-6G and F4/80 antibodies, respectively. Seven tumours were analysed from each group. Means ± SEM are shown. *p*-value: ****9.16945E−06; ***0.000573.

Similar results were obtained upon analysis of MLN8237-treated patient tumour implants from patient V35 and V29. These data conclusively demonstrate that MLN8237 treatment induced senescence (Fig 6A and Supporting Information Fig S13), the DDR based upon the formation of 53BP1 foci after drug treatment (Fig 6B), the SASP (Fig 6C and Supporting Information Table S3), where increases in GRO (CXCL1-3), IL-8 (CXCL8), Angiogenin, IL-6 and GRO-α (CXCL1) were observed by cytokine array.

To investigate whether the formation of the SASP boosts recruitment of phagocytic leucocytes to the senescent tumour tissues, we evaluated the infiltration of neutrophils and macrophages into the tumours treated with vehicle or MLN8237 by FACS analysis of Ly6G- and F4/80-stained leucocytes. MLN8237-treated tumours exhibited markedly increased recruitment of Ly6G^+^ neutrophils (*p* = 0.000573) and F4/80^+^ macrophages (*p* = 9.16945E-06) compared to vehicle control-treated tumour tissues (Fig 6D). The differences in mean recruitment of F4/80 or Ly6G cells to the tumour from seven mice are shown at the bottom of Fig 6D.

### Immune surveillance limits senescent tumour development

**Figure 7:**
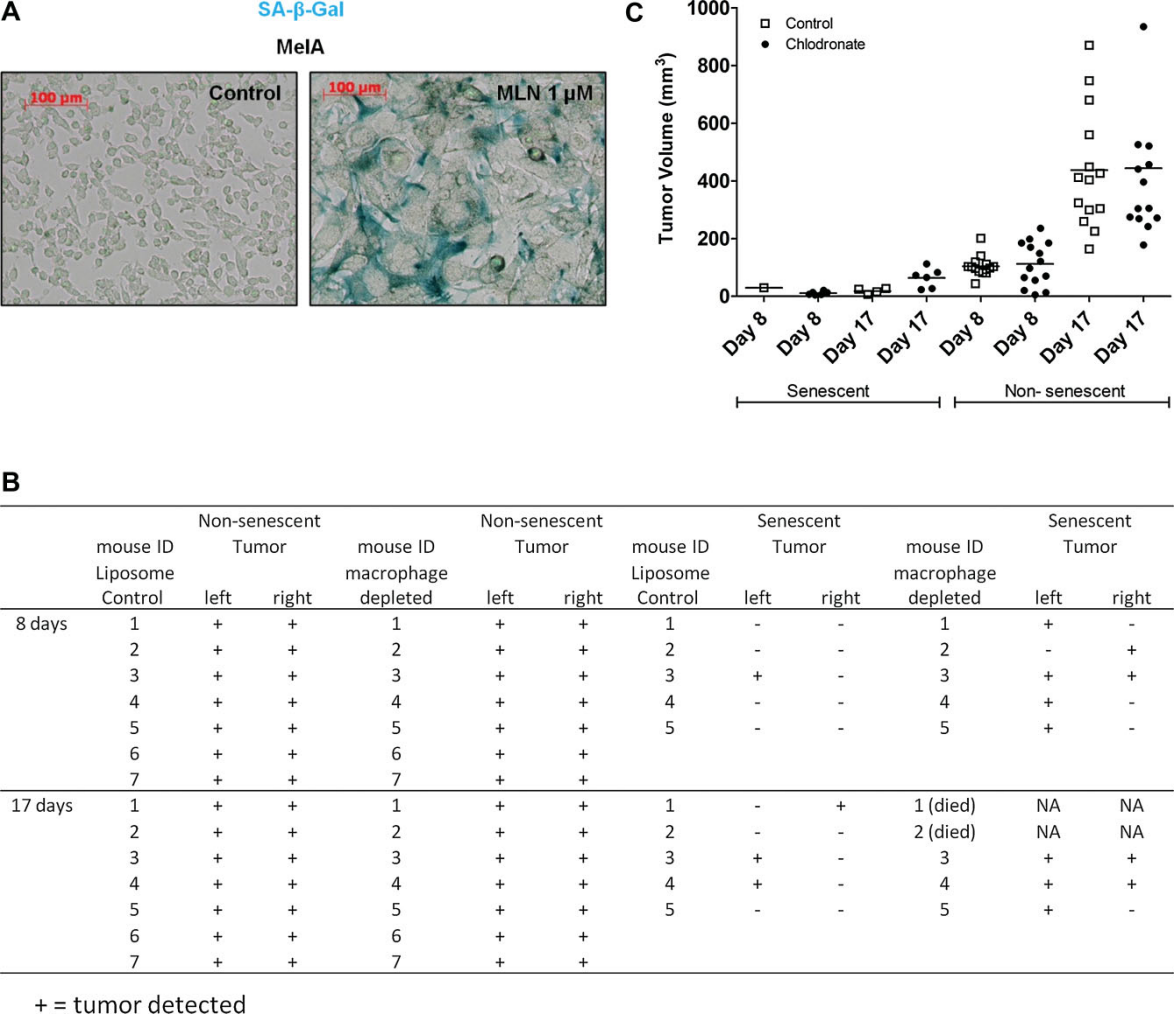
Macrophage depletion impairs senescence surveillance The mouse melanoma line, MelA, was treated with MLN8237 for 5 days and senescence was determined by β-galactosidase staining.MelA cells were pre-treated with MLN8237 for 1 week to induce senescence. Macrophages were depleted with 1 mg of clodronate in C57BL/6 mice. One day after clodronate treatment (macrophage depletion), 5 × 10^6^ MLN8237-pre-treated MelA cells were injected into C57BL/6 mice. Control mice were treated with liposome vehicle (*n* = 5). Non-pre-treated MelA cells were also injected into C57BL/6 mice with or without macrophage depletion as controls (*n* = 7). Tumour formation was monitored in each group of mice for 17 days.Tumour volume was measured once per week. The scatter plot shows the tumour volume for each mouse at day 8 and Day 17.

In the nude mouse model, we observed marked increases in macrophage and neutrophil recruitment to MLN8237-treated tumours (Fig 6D), where they presumably exhibit some anti-tumour activity. Athymic nude mice exhibit enhanced T-cell-independent activation of macrophages (Cheers & Waller, 1975; Mills et al, 2000), but recently CD4^+^T cells were implicated in the licensing of macrophages for clearance of senescent cells in immunocompetent mice (Kang et al, 2011). Due to its translational relevance, we sought to investigate the role of macrophages in the clearance of senescent melanoma cells in a fully immunocompetent mouse model. To this end, we utilized the immunocompetent C57Bl/6 mice and a spontaneously transformed mouse melanoma cell line derived from C57Bl/6 mice (MelA) (Bennett et al, 1987). MelA cells were pretreated with MLN8237 (1 µM) for 1 week to induce senescence (Fig 7A), then the drug pre-treated MelA cells or vehicle pre-treated MelA cells were injected into C57Bl/6 mice, which were either pre-treated with clodronate (to deplete macrophages) or with liposome carrier control. Eight days after senescent MelA cells were injected into mice, tumour nodules were present in 5/5 macrophage-depleted mice. In contrast, for the mice where macrophages were not depleted, tumour growth was observed in only one out of five mice (Fig 7B) injected with senescent MelA cells. After 17 days, more tumours developed in both groups (Fig 7B). Non-senescent (drug vehicle pre-treated) MelA cells formed tumours in all mice and the mean tumour volume was much greater than in the MLN8237-pretreated senescent MelA cells. However, macrophage depletion did not affect the tumour development in vehicle pretreated tumours (8 days *p* = 0.7222; 17 days *p* = 0.9405; Fig 7C). These data suggest that macrophages recruited into the tumour in response to SASP exhibit anti-tumour activity *in vivo* and consequently slow tumour growth. In contrast, non-senescent tumour cells appear to retain a type of immune privilege, escaping macrophage-mediated tumour surveillance.

### Disruption of IKKβ/NF-κB bypasses MLN8237-induced senescence

**Figure 8:**
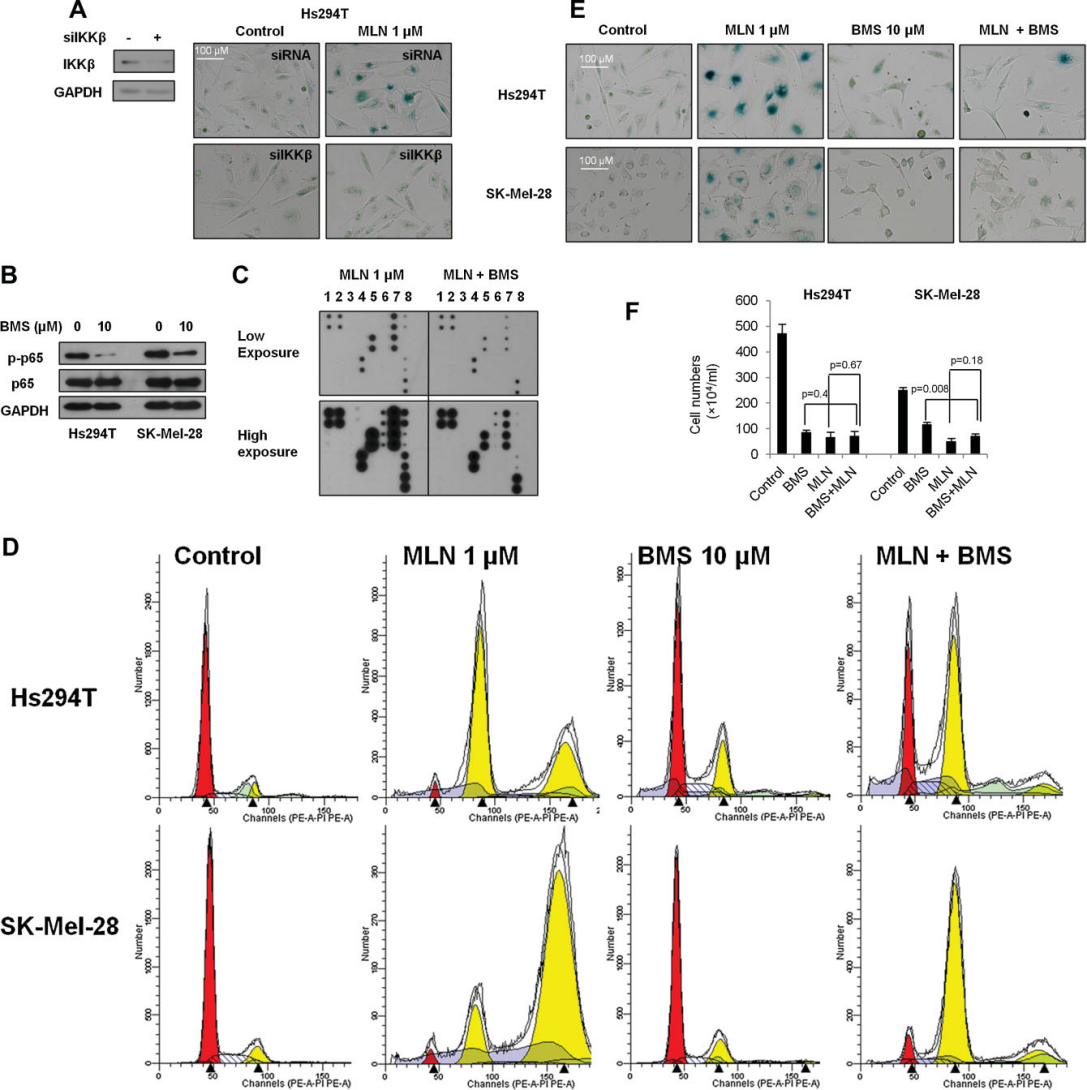
Disruption of IKKβ/NF-κB compromises drug-induced senescence Hs294T cells were transfected with IKKβ siRNA, and knockdown of IKKβ verified by Western blot. siRNA control or siIKKβ transfected cells were treated with 1 µM MLN8237 for 5 days, and senescence was evaluated by β-galactosidase staining.Hs294T and SK-Mel-28 cells were treated with the IKKβ inhibitor BMS-345541 (10 µM) for 48 h, and the level of p-p65 and p65 were analysed by Western blot.Hs294T cells were treated with 1 µM MLN8237 with or without 10 µM BMS-34554 for 5 days. After treatment, viable cells were counted and 5 × 10^5^ cells were seeded into 10-cm plates in DMEM F-12 with 10% FBS. Once cells attached, serum-containing medium was replaced with serum-free medium and cells were cultured overnight. Cytokine secretion into the medium was assayed by cytokine array.Hs294T and SK-Mel-28 cells were treated with 10 µM BMS-345541, 1 µM MLN8237, or both for 2 days and DNA content was examined by FACS.Hs294T and SK-Mel-28 cells were treated with 10 µM BMS-345541, 1 µM MLN8237, or both for 5 days. After treatment, senescence was evaluated by β-galactosidase staining.Hs294T and SK-Mel-28 cells were treated with 10 µM BMS-345541, 1 µM MLN8237, or both for 5 days, and the viable cells were counted using a haemocytometer. Data indicate mean values ± SD (*n* = 3). With the exception of Fig 8C, all experiments were performed in triplicate with high reproducibility and representative experiments are shown.

To address the significance of NF-κB activation on treatment outcome, we knocked down IKKβ to reduce NF-κB activity and observed that aurora kinase inhibitor-induced senescence was impaired (Fig 8A). IKKβ stable-knockdown cells gave rise to a similar phenotype (Supporting Information Fig S14). We also confirmed our results using the IKKβ inhibitor BMS-345541 (10 µM) to block the NF-κB p65 pathway (Fig 8B). When IKKβ activity was suppressed, the MLN8237-induced SASP was decreased (Fig 8C), polyploidy was reduced (Fig 8D), and less senescence was observed (Fig 8E). Although targeting IKKβ/NF-κB with BMS-345541 induces apoptosis in melanoma cells (Yang et al, 2006b), we did not observe synergistic effects on cell growth/survival when BMS345541 was combined with MLN8237 *in vitro* (Fig 8F), likely because blocking IKKβ reduces the induction of senescence by MLN8237, so the effect of combined treatment is largely the result of apoptosis induction by inhibition of IKKβ.

**Figure 9:**
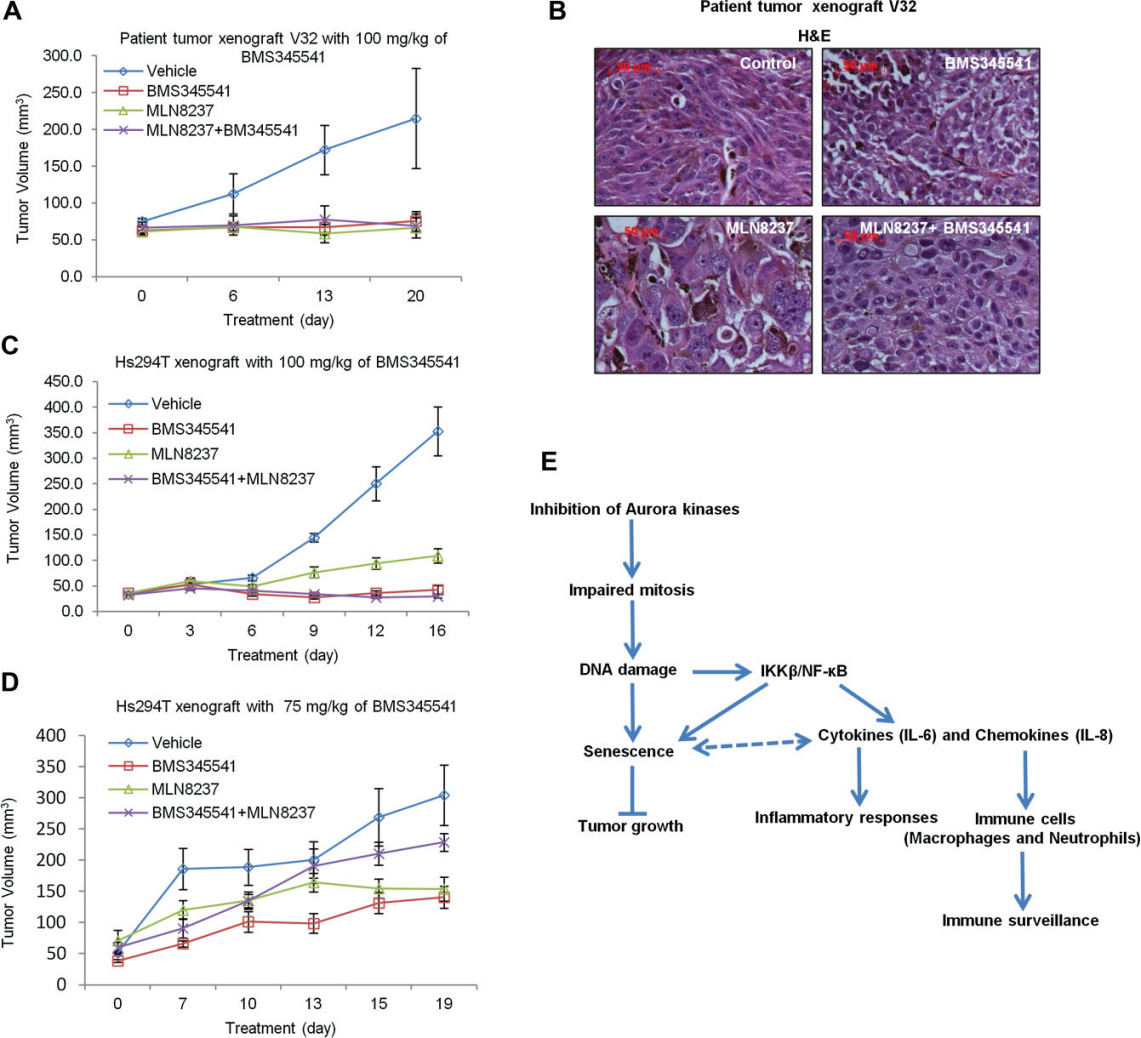
Combination of IKKβ/NF-κB inhibitor (BMS345541) and AURKA inhibitor (MLN8237) does not produce a synergistic inhibitory effect on tumour growth **A.** Patient tumour tissues (V32) were implanted subcutaneously into nude mice. After 1 week, tumour-bearing mice received daily oral doses of BMS-345541 (100 mg/kg) or MLN8237 (30 mg/kg) or both. Mean tumour volumes ± SEM are shown (*n* = 5).**B.** H&E staining of the V32 patient tumours from mice treated with vehicle, BMS345541, MLN8237, or both BMS345541 and MLN8237.**C,D.** Hs294T melanoma cells were injected subcutaneously into nude mice (2 × 10^6^ cells per mouse). After 1 week, tumour-bearing mice received daily oral doses of BMS-345541 [100 mg/kg (**C**) or 75 mg/kg (**D**)] or MLN8237 (30 mg/kg) or 30 mg/kg MLN8237 combined with 100 mg/kg BMS345541 (**C**) or 75 mg/kg BMS345541 (**D**). Mean tumour volumes ± SEM are shown. (*n* = 5).**E.** Diagrammatic representation of the proposed model of MLN8237-induced senescence and senescence surveillance by immune cells.

To extend our findings *in vivo*, we treated patient tumour-bearing mice with vehicle, IKKβ inhibitor (BMS345541 100 mg/kg daily), aurora kinase inhibitor (MLN8237 30 mg/kg daily), or both. After treatment, we observed no synergistic effects with combined treatment (Fig 9A). H&E staining demonstrated that disruption of IKKβ/NF-κB bypasses aurora kinase inhibitor-induced senescence (Fig 9B). Similar results were obtained in Hs294T-bearing mice with the same treatment (Fig 9C). Since BMS345541 treatment induces cell death, we reduced the dose of BMS345541 from 100 to 75 mg/kg once daily. When 75 mg/kg of BMS345541 was administered, we found that combined treatment impaired the growth inhibitory response compared to treatment with either single agent alone (Fig 9D).

## DISCUSSION

Cellular senescence is regarded as a tumour-repressive mechanism that limits the proliferation of damaged cells to stop neoplastic transformation at an early stage. Diverse stimuli can trigger senescence, including telomere shortening, DNA damage, oncogene activation, tumour suppressor inactivation, oncogene inactivation and tumour suppressor re-activation (Campisi & d'Adda di Fagagna, 2007; Kuilman et al, 2010; for review). While senescent cells undergo growth arrest, they remain metabolically active and secrete cytokines, chemokines and growth factors that may trigger various cellular responses (Coppe et al, 2008; Kuilman et al, 2010). Some cytokines, such as IL-6 and IL-8, are essential for maintenance of senescence (Acosta et al, 2008; Kuilman et al, 2008) but at high levels, these factors can contribute to tumour progression (Coppe et al, 2008). Other secreted pro-inflammatory factors have similar effects: VEGF stimulates migration, invasion and angiogenesis (Coppe et al, 2006) and GRO1(CXCL1) promotes tumour growth (Yang et al, 2006a). Mouse xenograft experiments provide evidence that senescent fibroblasts stimulate tumour growth when co-injected with premalignant cells (Krtolica et al, 2001; Liu & Hornsby, 2007). While tumour suppressor inactivation allows damaged cells to bypass OIS (Serrano et al, 1997), tumour cells retain the capacity to senesce (Goodwin et al, 2000). However, it is not clear whether induction of senescence limits or increases tumour growth *in vivo*. Also, the long-term effects of senescence on tumour development remain unclear.

In this study, we explored whether induction of senescence in human melanoma is a possible approach for cancer therapy. It has been shown that knockdown of either AURKA or AURKB induces cellular senescence (Huck et al, 2010; Kim et al, 2011). Our data reported here agree with prior studies showing an increased percentage of aurora kinase-positive cells in melanoma (Wang et al, 2010). To evaluate AURKA as a therapeutic target in melanoma, we targeted AURK in human melanoma tumour implants growing in mice using an AURK inhibitor currently in clinical trials for solid tumours. Our data show that the AURKA inhibitor, MLN8237, significantly reduces melanoma tumour burden. Although inhibition of AURKA in multiple myeloma induced both apoptosis and senescence (Gorgun et al, 2010), using the same inhibitor we did not observe significant apoptosis in response to MLN8237 in melanoma tumours *in vivo*. Further analysis showed that senescence is the major process affected by aurora kinase inhibition *in vitro* and *in vivo*, thus providing a good model with which to study the effect of senescence induction on tumour growth. Our results demonstrate that the induction of senescence blocked tumour growth in most of the tested melanoma patient tumour implants. More interestingly, when we suspended the treatment on a subset of these tumours, 50% did not relapse within 12 months. Among the relapsed tumours, 2/3 responded to a second round of treatment. These findings provide solid evidence that induction of senescence in tumours limits melanoma tumour growth in mice.

To investigate the mechanisms by which targeting aurora kinase induces senescence, we explored signalling pathways implicated in senescence. Previous studies reported that p53 and p21 play a critical role in senescence (Serrano et al, 1997; Sugrue et al, 1997). While both p53 and p21 were upregulated in wild-type p53 senescent cells, senescence was still induced in response to MLN8237 in mutant p53 melanoma cells, suggesting that p53 and p21 are not absolutely required for drug-induced senescence. To further extend these studies, we used a p53-specific inhibitor to block the p53-signalling pathway in MLN8237-treated cells. We found that the p53 inhibitor did not impair drug-induced senescence, indicating that other pathways are responsible for MLN8237-induced senescence.

It is well established that one of the hallmarks of senescence is DNA damage (Campisi, 2011). In the present context, knocking down AURKA or AURKB results in polyploidy (Chan et al, 2007; Chefetz et al, 2011; Manfredi et al, 2007, 2011) and polyploidy causes genomic instability [reviewed in (Storchova & Kuffer, 2008; Storchova & Pellman, 2004)]. Therefore, we hypothesized that the senescence induced by aurora kinase inhibitors results from the DDR. Our data demonstrate the formation of 53BP1 foci in senescent cells *in vitro* and *in vivo*, suggesting the occurrence of double-strand breaks (DSB). Since both ATM and ATR kinases can be activated upon DNA damage (d'Adda di Fagagna, 2008; for review), we investigated which of these two kinase pathways is responsible for drug-induced senescence. Our results show that the ATM/Chk2 pathway is activated upon drug treatment. Chk2 can trigger replicative senescence via either p53/p21 or other pathways in response to telomere dysfunction and DNA damage (Gire et al, 2004). Therefore, we conclude that the drug-induced senescence reported here is mediated by ATM/Chk2. Although the DDR induces senescence and cell cycle arrest, it also has the potential for promoting drug resistance. We observed that patient tumour V23 did not respond to a second round of treatment with MLN8054. Thus, in some cases, DNA damage may produce secondary hits to bypass senescence and lead to tumour progression (Tu et al, 2011). MLN8054 or MLN8237-induced polyploidy in conjunction with DNA damage may result in tumour relapse if the additional hits target oncogenes or tumour suppressors.

A recent study showed that immune surveillance of senescent pre-malignant hepatocytes, as mediated by macrophages and T cells, limits tumour formation (Kang et al, 2011). Previous studies also demonstrated that the immune response contributes to the induction of senescence or the clearance of senescent tumour cells to limit tumour growth (Rakhra et al, 2010; Xue et al, 2007). We have demonstrated here that senescent melanoma cells secrete IL-6, IL-8 [CXCL8] and GRO [CXCL1, 2 and 3] *in vitro* and *in vivo*, which are associated with the pro-inflammatory response and recruitment of neutrophils and macrophages to senescent tumour cells. According to the premalignant model by Xue et al, we expected that the recruitment of immune infiltrates in response to inflammatory cytokines and chemokines would aid in the removal of senescent tumour cells that were produced in response to MLN8237. However, in this model, we observed tumour growth inhibition, but not significant tumour regression in most tumour-bearing nude mice.

To address this issue in a more relevant immune model, we depleted macrophages in immunocompetent mice and then injected senescent mouse melanoma cells. More tumours developed in macrophage-depleted mice compared to mice that retained macrophages. However, macrophages did not inhibit growth of tumours arising from tumour cells not pre-treated with MLN8237 to induce senescence. These data suggest that in this model, macrophages play a critical role in the clearance of senescent tumour cells but play a limited protective role in immune surveillance of non-senescent tumour cells. To further explore the contribution of the immune cells to surveillance of senescent and non-senescent tumour cells, mice with engineered deficiencies of specific immune cells need to be used.

In addition, Xue et al reported that p53 restoration can trigger tumour clearance through p53 dependent senescence (Xue et al, 2007). In contrast, in our model, the aurora kinase inhibitor-induced senescence is p53 independent. Therefore, although p53 was induced in p53 wild type melanomas, its reactivation did not lead to tumour clearance.

It is clear that NF-κB is commonly activated in tumour cells in response to therapy-induced DNA damage and this can confer chemotherapy resistance (Janssens & Tschopp, 2006; for review). Recent studies demonstrate that NF-κB also contributes to maintaining cellular senescence (Rovillain et al, 2011; Wang et al, 2009). We found that NF-κB was activated in drug-induced senescent melanoma cells, which conferred development of the SASP. However, previous studies have reported inhibition of AURKA downregulates NF-κB (Briassouli et al, 2007; Chefetz et al, 2011).Therefore, we predicted that in our model, the induction of NF-κB is not due to the inhibition of AURKA, but is in response to the ATM/Chk2-mediated DDR since ATM can mediate NF-κB activation upon DNA damage (Li et al, 2001; Wu et al, 2006).

To explore whether blocking NF-κB would increase apoptosis in senescent cells, we blocked IKKβ with either siRNA or a small-molecule inhibitor. Surprisingly, drug-induced senescence was impaired when IKKβ/NF-κB was suppressed. Mechanistic analysis showed that drug-induced formation of polyploidy was blocked. A high dose of IKKβ inhibitor impaired MLN8237 induced senescence but did not affect the therapeutic outcome due to the pronounced induction of cell death in response to 100 mg/kg/day of the IKKβ inhibitor. However, administration of a lower dose of IKKβ inhibitor impaired the therapeutic outcome in response to MLN8237 when the two drugs were combined, as compared to treatment with single agent alone. Similar results have been reported in which disruption of the NF-κB-mediated SASP leads to chemo-resistance in a mouse lymphoma model (Chien et al, 2011; Jing et al, 2011). Another mouse model demonstrated that NF-κB inhibition by either genetic depletion or a pharmacological inhibitor attenuates DNA damage and delays DNA damage-induced senescence (Tilstra et al, 2012). In addition, induction of NF-κB can promote senescence (Rovillain et al, 2011). Taken together, the data show that NF-κB activation is correlated with DNA damage-induced senescence.

In summary, we have shown that targeting aurora kinase activity in melanoma cells impaired mitosis, caused DNA damage, induced senescence and inhibited tumour growth. DNA damage-mediated IKKβ/NF-κB activation promotes the SASP and boosts the immune response, which may remove the senescent tumour cells (Fig 9E). Our data predict that carefully designed delivery of aurora kinase inhibitors may effectively slow tumour growth via senescence to provide effective therapy for some melanoma patients. However, since the induction of senescence does not result in tumour regression and elimination, these inhibitors may need to be used in combination with other therapeutic agents.

## MATERIALS AND METHODS

### Cell culture and chemical reagents

Melanoma cell lines A375, Hs294T, SK-Mel-2, SK-Mel-5, SK-Mel-28 and WM115 were obtained from American Type Culture Collection (ATCC, Manassas, VA) and cultured in DMEM F12 supplemented with 10% foetal bovine serum, 2 mmol/L glutamine, 100 µmol/L MEM nonessential amino acids (Invitrogen Corporation, Carlsbad, CA) and 1 mmol/L sodium pyruvate (Sigma–Aldrich, St. Louis, MO). The mouse melanoma cell line MelA (the kind gift of Dorthea Bennett) was cultured in RPMI with 10% foetal bovine serum. Aurora kinase inhibitors MLN8054 and MLN8237 (Alisertib) were obtained from Millennium Pharmaceuticals, Inc. The IKKβ inhibitor BMS-345541 was described previously (Yang et al, 2006b) and was synthesized in the Vanderbilt University Chemical Biology Core laboratory. The ATM inhibitor KU-55933 was obtained from EMD Millipore (Billerica, MA). The p53 inhibitor pifithrin-α (PTF-α) was obtained from Tocris Bioscience (Ellisville, MO), and the pan-caspase inhibitor Z-VAD-FMK was obtained from Molecular Probes (Eugene, OR).

### Western blot

Cells were lysed by ice-cold RIPA buffer containing proteasome-inhibitor cocktail and phosphatase-inhibitor cocktail. The lysates were then centrifuged at 4°C and the supernatant was collected. Protein samples were separated by SDS–PAGE, transferred onto a nitrocellulose membrane, and probed with an appropriate antibody. Antibodies to AURKA (#4718), AURKB (#3094), p53 (#2524), p21 (#2947), γ-H2A.X (#9718), p-Chk1 (#2349), p-Chk2 (#2661), Chk2 (#2662), ATM (#2873), p-p65 (Ser536, #3033), p65 (#4764), IκB-α (#9247), p-AKT (Ser473, #9271), AKT (#2697), p-ERK (Thr202/Tyr204, #9101), ERK (#9102), p-p38 MAPK (Thr180/Tyr182, #9211), p38 MAPK (#9212), p-STAT3 (#9145), STAT3 (#9132) and GAPDH (#2118) and an HRP-conjugated secondary antibody were obtained from Cell Signaling Technology (Beverly, MA). An antibody to p63 (#53039) was obtained from Abcam (Cambridge, MA). An antibody to p16 (C-20, sc-468) was purchased from Santa Cruz Biotechnology (Santa Cruz, CA). An antibody to p73 (#A300-126A) was obtained from Bethyl Laboratories, Inc (Montgomery, TX). An antibody to p-IKKβ (Ser177, #MA5-14857) was purchased from Pierce Biotechnology (Rockford, IL). The target protein was examined by chemiluminescence (Cell Signaling Technology). Control siRNA (#6568) and siRNAs targeting ATM (#6328), Chk2 (#6276), or IKKβ (#6377) were obtained from Cell Signaling Technology.

**The paper explained**

PROBLEM:

Cellular senescence was originally believed to be a cell culture artefact that limits proliferation of normal cultured cells after a finite number of divisions. Recent studies show that removal of accumulating senescent cells from organs can delay ageing-associated disorders. Nonetheless, while OIS is considered to be a protective mechanism against tumourigenesis, production of cytokines and growth factors by senescent cells as a result of NF-κB activation may contribute to either tumour formation or regression. In this study, we sought to clarify whether induction of senescence represents a viable approach for cancer therapy.

RESULTS:

Here, using a mouse model with implantation of human melanoma tumour tissues taken from 19 patients, we observed that targeting aurora kinase with a small-molecule inhibitor impaired mitosis, induced tumour senescence and markedly blocked tumour growth in the patient tumour implants. Fifty percent of the treated tumours did not progress within 12 months. Like other anti-cancer therapies, induction of senescence activated IKKβ/NF-κB pathway, which may confer resistance to apoptosis. However, blockade of the IKKβ/NF-κB pathway induced apoptosis, but combined inhibition of aurora kinase and IKKβ led to reversal of the drug-induced senescence.

IMPACT:

Our findings suggest that carefully designed delivery of aurora kinase inhibitors may effectively slow tumour growth via senescence to provide effective therapy for some melanoma patients. Identification of an appropriate agent to use in combination therapy with aurora kinase inhibitors to increase tumour cell killing and tumour regression will be an important future consideration.

### Cell viability assay

Cells were trypsinized, collected, treated with trypan blue and the cells that excluded trypan blue (viable cells) were counted using a haemocytometer.

### Senescence assay

Senescence was examined by a senescence-associated β-galactosidase assay (Sigma–Aldrich). Briefly, cells or tissue were washed with phosphate-buffered saline (PBS) buffer and fixed with fixation buffer for 7 min. Cells were then washed three times with PBS and stained with staining buffer at 37°C.

### Cell cycle analysis by FACS

Cells were washed with PBS and fixed by 70% cold ethanol at −20°C > 2 h. After fixation, cells were stained by propidium iodide (PI) staining solution (Triton X-100, RNase A, propidium and EDTA) at room temperature for 30 min. After staining, cell content was analysed by FACS using a LSRII (BD Biosciences, San Jose, CA).

### Apoptosis assay

Apoptosis was assessed by flow cytometry after co-staining of cells with Annexin V-PE and 7-AAD in accordance with manufacturer's recommendations (BD Biosciences). Annexin V-positive, 7-AAD negative cells and double-positive cells were considered to be in early and late stages of apoptosis, respectively. Cells negative for both markers were identified as viable.

### Macrophage and neutrophil analysis by FACS

Tumours were dissociated in PBS pH 7.2, 0.5% BSA containing Collagenase I (EMD Millipore, Billerica, MA), Dispase II (Roche Applied Science, Indianapolis, IN) and DNase I (Sigma, St. Louis, MO) using the GentleMacs Dissociator (Miltenyi Biotec, Cambridge, MA). Cells were filtered and washed, then stained for 30 m on ice with anti-CD11b FITC, Ly-6G PE (BD Biosciences) and F4/80 PerCp-Cy5.5 (eBioscience, San Diego, CA). Analysis was performed on a custom 5-laser LSRII (BD Biosciences).

### siRNA transfection

Cells were cultured in serum and antibiotic free medium. siRNA (50 nM) and 7.5 µl of transfection reagent (lipofectamine RNAiMAX, Invitrogen) were each diluted in 500 µl of medium. After 5 min, the siRNA solution and RNAiMAX solution were combined and incubated at room temperature for 20 min, the siRNA and RNAiMAX complexes were added to the cultured cells. The cells were cultured at 37°C for 48 h prior to fixation and SA-β-Gal staining or lysis and Western blot.

### NF-κB luciferase assay

NF-κB Gaussia-luciferase reporter Hs294T cells were generated in our lab and cultured in DMEM F12 with puromycin (Yang & Richmond, 2009). Cells were treated with MLN8237 or vehicle for 5 days and 20 µl of the culture medium was added in a luminometer tube. Gaussia luciferase assay reagent (Targeting systems, CA; 50 µl) was added to the culture medium and the luciferase activity was measure on a luminometer.

### Immunocytochemistry

Cells (10^4^) were seeded on a micro cover glass and treated with MLN8237 or vehicle control as indicated. After treatment, cells were washed with PBS buffer then fixed with cold methanol for 15 min at −20°C. After two quick washes with cold PBS, the slides were blocked with 5% normal donkey serum and 0.3% Triton X-100 in PBS for 1 h at room temperature. The slides were then incubated with 53BP1 or γ-H2A.X antibody (Cell Signaling) at 4°C. After overnight incubation, the slides were washed with PBS-T solution (PBS with 0.1% Tween-20) and then incubated with Alexa Fluor 594 secondary antibody (Molecular Probes, Invitrogen, Carlsbad, CA) for 1 h at room temperature. Cells were subsequently washed three times with PBS-T, counterstained with Hoechst, and mounted with Prolong Gold anti-fade reagent (Invitrogen). Photomicrographs were captured using an AxioVision microscope (Zeiss).

### Cytokine array

Hs294T cells were treated *in vitro* with 1 µM MLN8237 or vehicle control for 5 days. After treatment, 5 × 10^5^ viable cells were seeded back into a 10-cm dish in DMEM/F12 with 10% FBS. When cells attached, serum-containing medium was replaced with serum-free medium. After overnight incubation, levels of cytokine and chemokine were evaluated by cytokine array. Cytokine array (RayBioTech, Norcross, GA) analysis was performed according to the RayBioTech procedural manual. Conditioned medium (100 µl) was used to determine the levels of cytokines and chemokines. The tissue cytokine array was performed using tumour lysates from patient tumour V35 treated with MLN8237 (30 mg/kg each day) or vehicle for 25 days. Total protein (250 µg) was used to evaluate the levels of cytokines and chemokines.

### Patient characteristics

The patient information was described previously (Su et al, 2012). The study was approved by the Institutional Review Board of Vanderbilt University and informed consent was obtained from each patient prior to surgery.

### Orthotopic implant tumour model

All of the animal experiments were performed in accordance with the Vanderbilt University Institutional Animal Care and Use Committee (IACUC) guidelines and regulations. The experiments were approved under the IACUC protocol number M/10/034.

The orthotopic implant tumour model was described previously (Su et al, 2012). Briefly, when tumour size reached 50–100 mm^3^, mice were given MLN8054 (60 mg/kg, QD) or MLN8237 (30 mg/kg, QD) or vehicle control (water) once daily by oral gavage. Tumour growth and mouse body weight were measured once a week, and when control or treated tumours reached a size of 1.5 cm diameter, mice were sacrificed and tumours were collected for analysis.

### Tissue micro-array (TMA) analysis

The preparation of TMA was described previously (Su et al, 2012). IHC staining was performed to analyse Ki67. The Vector ABC kit and NovaRED substrate kit (both from Vector Laboratories, Burlingame, CA) were used for IHC detection. Immunofluorescence staining was performed to analyse p-AURKA (developed by Millennium), IL-6 (#ab6672, Abcam), IκB-α, and 53BP1.

### Macrophage depletion

One milligram of clodronate in a liposome formulation (Encapsula NanoSciences, TN) was delivered by intra-peritoneal injection into mice to deplete macrophages. The reagent was injected once per week for 2 weeks. Controls received the liposome formulation alone.

### Statistical analysis

Statistical significance was determined using the Student's *t*-test, Fischer's Exact test, or two-way ANOVA analysis. *p* < 0.05 was considered significant.

## Supplementary material

Detailed facts of importance to specialist readers are published as ”Supporting Information”. Such documents are peer-reviewed, but not copy-edited or typeset. They are made available as submitted by the authors.
