# Supplementary material for: Targeting aurora kinases limits tumour growth through DNA damage-mediated senescence and blockade of NF-κB impairs this drug-induced senescence
Source: EMBO Mol Med. 2012 Nov 25;5(1):149–66. doi: 10.1002/emmm.201201378 (PMC3569660; doi:10.1002/emmm.201201378)
Supplement: Supplementary file 1 [file emmm0005-0149-SD1.pdf]

Manuscript EMM-2012-01378

Targeting Aurora Kinases Limits Tumor Growth through DNA Damage-Mediated Senescence and Blockade of NF-κB Impairs this Drug-Induced Senescence

Yan Liu, Oriana E. Hawkins , Yingjun Su, Anna E. Vilgelm, Tammy Sobolik, Yee-Mon Thu, Sara Kantrow, Ryan C. Splittgerber, Sarah Short, Katayoun I. Amiri, Jeffery A. Ecsedy, Jeffery A. Sosman, Mark C. Kelley, and Ann Richmond

Corresponding author: Ann Richmond, Vanderbilt University

Review timeline:

Submission date:	14 March 2012
Editorial Decision:	16 April 2012
Revision received:	19 July 2012
Editorial Decision:	22 August 2012
Revision received:	08 October 2012
Accepted:	11 October 2012

Transaction Report:

(Note: With the exception of the correction of typographical or spelling errors that could be a source of ambiguity, letters and reports are not edited. The original formatting of letters and referee reports may not be reflected in this compilation.)

1st Editorial Decision 16 April 2012

Thank you for the submission of your manuscript "Targeting Aurora Kinase Limits Tumor Growth through Induction of NF- B-Associated Senescence" to EMBO Molecular Medicine. We have now received reports from the three referees whom we asked to evaluate your manuscript. You will see that the Reviewers find the topic of your manuscript potentially interesting. However, they also raise significant concerns on the study, which should be addressed in a major revision of the manuscript.

Importantly, reviewer #2 highlights that the conclusion regarding NFkB-induced senescence and reduction in tumor growth should be further substantiated. In addition, reviewer #1 notes that secondary tumor formation should be investigated.

On a more editorial note, EMBO Molecular Medicine requires that the description of all reported data that includes statistical testing must state the name of the statistical test used to generate error bars and P values, the number (n) of independent experiments underlying each data point (not replicate measures of one sample), and the actual P value for each test (not merely 'significant' or 'P < 0.05') (please see http://onlinelibrary.wiley.com/journal/10.1002/(ISSN)1757-4684/homepage/ForAuthors.html#data2 for more information). Please also include a Table of Contents as the first page of your Supporting Information.

Given the balance of these evaluations, we feel that we can consider a revision of your manuscript if you can convincingly address the issues that have been raised within the space and time constraints outlined below.

Revised manuscripts should be submitted within three months of a request for revision. They will otherwise be treated as new submissions, unless arranged otherwise with the editor.

I look forward to seeing a revised form of your manuscript as soon as possible.

Yours sincerely,

Editor
EMBO Molecular Medicine

***** Reviewer's comments *****

Referee #1 (Comments on Novelty/Model System):

The technical quality of the study is mixed, though could be improved through better controls and clarifications in the text. The role of senescence in therapy response is interesting and novel in the context studied, but many of the mechanisms have been seen in other contexts. Models are adequate.

Referee #1 (Other Remarks):

Liu et al. used patient-derived melanoma-implants and melanoma cell lines to investigate the biological responses of tumor cells to treatment with Aurora kinase inhibitors MLN8054 and MLN8237. The authors demonstrate that Aurora Kinase A inhibition induces a senescence response, which in vitro was associated with DNA damage response signaling, increased NF B activity and presence of a SASP. Durable anti-tumor responses in the orthotopic xenograft model were achieved with 3 weeks of inhibitor treatment (in the absence of marked apoptosis), suggesting that that the induction of tumor-cell senescence is sufficient for long-term therapeutic responses in this model.

Given the recent interest in senescence biology, its involvement in drug responses, and the importance of the disease context, the study touches on some timely topics. From a pre-clinical perspective, the authors at times use a novel 'patient-derived' orthotopic implant model together with a clinically relevant, second generation Aurora Kinase inhibitor (MLN8237). Very few published studies of MLN8237 currently exist, therefore the manuscript is of likely interest to the AKi field. A phase II clinical trial of MLN8237 in unresectable stage III-IV melanoma is currently recruiting (Study NCT01316692, Note: the trial PI, Jeffery Sosman, is an author on this manuscript), thus publication of these pre-clinical data would be timely, and may contribute to further defining the mechanism of anti-tumor activity of these compounds.

I am unaware of any study that shows that NF B inhibition can prevent Aurora-A inhibitor induced -polyploidy and -senescence, which represents a novel advance. However, the study is not particularly novel for its insights into senescence biology: it is known that Aurora kinase inhibitors can induce senescence, that melanocytic nevi can exhibit a senescence response and that NF B controls the SASP, and that disruption of NF B can promote a poor response to therapy. Additionally, some data are not particularly convincing (see below).

Major Comments
1. The authors state that mice were implanted with tumors that had been surgically resected from 19 patients and treated with AKis over 4 weeks, defining a positive response as 50% decrease in tumor growth vs vehicle control. Thus tumors can technically progress under therapy and still be considered as responders (indeed this occurs in 2/3 tumor growth curves presented in Figure 1). The growth curve for only one tumor treated with each AKi (MLN8054 and MLN8237) was presented, and the end point classification for all tumors is presented in the table. The actual tumor/patient shown in Figure 1a are not defined, thus it is not possible to cross compare with the table. The complete tumor growth/dose response curves for the remaining 17 patients should also be shown, as tumor growth rates and responses relative to time 0 (i.e. tumor regression) cannot be gauged from the presented data.

2. Figure S2 suggests that there is no direct correlation between therapeutic responsiveness and AURKA inhibition - have the authors investigated possible mechanisms of non-responsiveness? Do non-responding tumors also display the enlarged cellular size and multi-nuclear characteristics of responding tumors? Do the 'relapse' tumors that are non-responsive to the second round of therapy display these characteristics? Other than the morphological changes associated with senescence, do the authors have any data supporting the induction of senescence in vivo (for example SA Gal positivity...this is shown for Hs294T, but not for any of the implants)? How can the authors rule out the possibility that the anti-tumor activity due to an off target effect?

3. The authors mention that durable responses can be induced following short term AKi treatment without the generation of secondary tumors, yet the authors present no evidence that untreated or vehicle-treated tumor implants are capable of secondary tumor formation.

4. The authors imply that immune infiltration is due to the SASP but provide little data in support of this notion. Nor do the authors provide data suggesting that neutrophils or macrophages contribute to the anti-tumor effect of MLN8237 (i.e. by antibody-based neutrophil depletion). Did co-treatment with MLN and BMS affect immune infiltration in vivo or any of the in vivo biomarkers for senescence, the SASP or Aurora kinase inhibition? Also, the quantification does not agree with the images shown and the numbers don't make sense - there are only a few infiltrating cells. If the quantification is indeed correct, it seems that the IHC images are not representative.

5. In figure 3H, I cannot see any difference in SA Gal positivity with siATM, siChk2 or siATM+siChk2 in Hs294T, thus it is difficult to accept the conclusion that senescence is dependent upon ATM or Chk2 in these cells. Quantification of the percentage of SA Gal positive cells would be helpful. Similarly, I don't see a difference between H2AX staining in control and MLN-treated cells (Figure 5). Quantification is required (% nuclei with foci).

6. The authors claim that there is no synergistic effect between MLN and BMS, but what happens if sub-optimal doses of drug are used? In addition, if NF B inhibition bypasses the senescence response, would one expect tumor outgrowth rather than synergistic inhibition? The DNA content analyses and inhibition of senescence support the idea that cells have re-entered the cell cycle, but is not really reflected in Fig 6F or 6G. Is NF B is expressed in the recipient stroma? Is it possible to inhibit NF B in a tumor cell-autonomous manner (through transduction of Hs294T with shRNAs)? Why are single time points shown and not growth curves? Again, the patient xenograft ID is not defined.

7. The authors should improve their discussion of the literature. Previously published studies of interplay between aurora kinase A and NF B signaling were not discussed or mentioned. The findings of the present study oppose those of Chefetz et al (Cell Cycle 2011), who showed in epithelial ovarian cancer stem cells that Aurora Kinase A inhibition (using MK-5108) induces polyploidy cell cycle arrest, with decreased NF B activity, decreased cytokine production and accumulation of I B . The study by Chefetz et al was not discussed in the present manuscript. Similarly, Aurora Kinase A has been shown to directly phosphorylate I B . (Briassouli et al, Cancer Res. 2007), yet Liu et al. show degradation of I B with AKi treatment. Liu et al. must be careful to avoid overstating the generality of their findings or extensively extrapolating the findings of others to their study.

Minor points
9. Only one mouse for V18 and four mice for V24 are shown in Table 2. Where are the data for V18A1, 2, 4 or 5, and V24A5?

10. I do not agree with the authors' impact statement that their 'data demonstrate the contribution of the NF B pathway to chemotherapy induced senescence and chemosensitivity', as inhibition of Nf B, despite inhibiting some senescence characteristics, did not affect the therapeutic outcome in the presented model. The authors state twice on 'the paper explained' page that therapy-induced senescence limits tumor growth and will not result in secondary tumors, yet produce no data demonstrating that their model is capable of secondary tumor formation.

11. Some of the experiments are missing controls or could present the data more clearly.

- Figure 2E - Authors need to label the cell lines either above or below the western blots. It's insufficient for readers/reviewers to assume an order. Please indicate p63 and p73 bands (or size markers) on the western blots.
- Figure 3: Authors need to include 'total' Chk2 and Chk1 on western blots. (3C,D and G)
- Figure 4: GM-CSF is not indicated on the cytokine array.
- Figure 5: I cannot see any difference in H2AX staining between control and MLN treated samples. Authors please provide quantification - i.e. number of nuclei with foci.
- Figure 6: need a 'total' p65 control blot.

Referee #2:

This paper aims to address an important and interesting issue in tumor cell biology: Can therapy-induced senescence result in tumor promotion or tumor progression?
Mice with ectopically transplanted human melanomas were treated with the Aurora A inhibitors MLN8054 and MLN8237. Interestingly, 8 out of 15 tumors (derived from in total 4 patients) responded to the treatment, while 7/15 relapsed. Further studies with cell line-induced tumors showed that MLN treatment induced senescence driven by the ATM/Chk2 pathway. This is all very interesting and well-performed work. But then the authors take a big leap in concluding that the reduction in tumor growth is a consequence of NF-kB induced senescence. In fact, this is the main conclusion (as reflected by the title) of the manuscript.

My major problem is that this conclusion is not substantiated by the data shown in figure 6. While inhibition of NF-kB by BMS inhibits the typical senescent features (b-galactosidase activity, SASP) induced by MLN treatment, BMS treatment itself causes a massive reduction in cell number and tumor shrinkage and it is therefore impossible to conclude if the effect of MLN treatment on tumor growth is mediated via NF-kB induced senescence. The authors should be more precise and state that their data show correlations.

Other points:
On page 6 the authors conclude that the levels of Aurora A and B are significantly higher in melanoma cell line than in normal melanocytes (Suppl fig 1). Without information on the mitotic index of the treated cells this conclusion cannot be drawn. If the melanoma cell lines cycle faster than the NHEM cells, more cells will accumulate in mitosis after 16h treatment with nocodazole. Expression of Aurora A and B is highest in mitosis and the high Aurora levels in the tumor cell lines could thus be explained by a higher number of mitotic cells. The authors should therefore collect mitotic cells by mitotic shake-off and prepare cell extract thereof. In line with this, to what extend are the Aurora levels in the cell lines representative of the levels in the patient-derived tumors transplanted into the mice?

Based on the IF pictures presented in figure 1C I am unable to appreciate the effect of the MLN drugs on pAurA or the mitotic spindles.

Page 7: ....'exhibited greatly enlarged cellular size and cells were often multi-nucleated, which are both characteristic of senescence'.
The authors should be aware that these are also characteristics of failed cell division.

7/15 tumors relapsed after drug administration, but only 3 of the 7 were tested in a second regime of MLN8054. To conclude anything on acquired drug-resistance all 7 tumors should have been tested.

In figure 2B the authors show that the apoptotic index of SK-Mel2 cells increases from approx. 1% to 25% after 72 h of treatment with MLN. Yet, on page 8 they conclude that apoptosis was not significantly increased. This maybe true for the other cell lines but not for SK-Mel2.

I miss the point of the TNFalpha experiment (fig. 7). To me it shows that two cell lines treated with MLN are responsive to TNFalpha treatment. I fail to see how this can be used to conclude that "induction of IKKb/NK-kB reinforces drug-induced senescence".

Page 15: "These findings provide solid evidence that induction of senescence in tumors are highly

resistant to apoptosis, as in melanoma, can limit tumor growth". I find this a puzzling remark since on page 12 it is mentioned that BMS induces apoptosis in melanoma cells.

Referee #3:

Liu et al report that Aurk inhibition triggers a senescence response in primary xenografts of human melanoma. Senescence induction was shown to be dependent on DNA damage signaling via ATM/Chk2 and furthermore on NfKB signaling. The authors also show that senescent melanomas secrete chemo- and cytokines that lead to the attraction of lecucocytes into the tumor tissue.

Overall, this is a very nice piece of work. The chosen experimental outline is sound and the presented data is comprehensive and sufficient to support the conclusions. From a general point of view it is a downside that several individual aspects that are nicely brought together as one unit in this study, have been published before. For example, it was shown that Aurk inhibition can result in senescence induction. Also, it was shown that NfKB is important for senescence induction in different settings and that NfKB signaling is crucial for the senescence associated secretory phenotype. Nevertheless, as this study was conducted in a very relevant in vivo system (primary melanoma xenografts) and the presented results have a high potential for clinical development, I am in favor of publication in Embo Mol Med if the following major and minor points can be addressed:

Major points:

1. What are the reasons for acquired drug resistance, e.g. in tumor V23P3A? Data or at least a discussion regarding this point is needed.
2. The authors describe higher leucocyte infiltration in senescent melanoma xenografts but only a "steady state" level of tumor size is observed and no tumor remissions. Do the leucocytes indeed contribute to long term tumor control ? How does the growth curves look when leukocyte function is inhibited, e.g. via antibodies ? How do the authors explain that there are no real tumor remissions induced by the infiltrating immune cells ? A study by Scott Lowe and colleagues has shown that senescence induction by p53 reactivation led to rapid tumor clearance via different innate immune cells. Is there a disctint secretory phenotype of senescent melanoma cells in tumors trerated with Aurka inhibitors ? As all studies were done in nude mice it is also conceivable that the adaptive immune system might be needed (see Kang et al., Nature 2010) to get full immune responses against senescent melanoma cells after Aurk inhibition. Obviously, studies in immunocompetent mice can¥t be conducted with primary human melanomas, however a discussion regarding this point would help.
3. The authors state "These results suggest that p53, p21, and p16 are not essential regulators of drug-induced senescence". As only Aurk inhibition was tested the statement should be relativized.

Minor points:

1. The introduction to the manuscript is somehow unidirectional, in particular regarding the SASP. It is well established that the SASP can also have antitumorigenic effects, for example as shown by work from Scott Lowe and others (Xue et al., Nature 2007, Kang et al., Nature 2011).
2. It is mentioned that a recent study by Kang et al showed that senescent cells are cleared by macrophages. My unserstanding is that this study showed that precancerous senescent hepatocytes are cleared through a CD4 T-cell response that is dependent on macrophages as effector cells. This should be specified. Along the lines the sentence "We predict that the recruitment of immune infiltrates in response to inflammatory cytokines and chemokines aids the removal of senescent tumor cells in vivo (Kang et al, 2011)" could be misleading for the reader, as the indicated study by Kang et al showed indeed already that secreted factors from senescent hepatocytes recruit immune cells which then clear senescent hepatocytes. The manuscript would benefit from a more precise discussion of the literature.

3. Fig. 1C - better resolution is needed
4. Fig. 1E - define the magnification (please provide figures with the same magnification), show multinuclei with arrows
5. Fig. 2E- label names of cell lines in the western blot

6. Fig. 3B - define MLN treatment, similar to fig.5B
7. Fig. 4A - GM-CSF is not defined on the array
8. Fig. 4D - number of days specified in the figure differs from information in the text
9. Fig. 5E -please specify the used marker for neutrophil staining
10. Information regarding figures: 2A, 3A, 3F-G; 4C, 6A, 6D, 6F, 7B is missing in the material and methods part.

1st Revision - authors' response 19 July 2012

Thank you very much for reviewing our manuscript entitled "Targeting Aurora Kinases Limits Tumor Growth through DNA Damage-Mediated Senescence and the NF-κB Inhibition Impairs Senescence". We appreciate the reviewers' suggestions and comments. In response to the reviewers' comments we have completed a series of *in vitro* and *in vivo* studies to better support our conclusions.

Editor:
Reviewer #2 indicates the conclusion regarding NFkB-induced senescence and reduction in tumour growth should be further substantiated. In addition, reviewer #1 notes that secondary tumour formation should be investigated.

Response: We have completed an *in vivo* study to further explore the combined treatment with the aurora kinase inhibitor, MLN8237, and the IKKβ inhibitor, BMS345541. The results of this study are found in Figure 9 and the associated results section of the manuscript. In regards to the question of secondary tumour formation, we communicated with reviewer 1 through the editor for definition of the phrase "secondary tumour". When we refer to secondary tumour, we referred to drug-induced spontaneous tumour formation. We have revised the manuscript to reflect this definition, accordingly.

Referee #1:

Major Comments

Question: *1.The authors state that mice were implanted with tumours that had been surgically resected from 19 patients and treated with aurora kinase inhibitors over 4 weeks, defining a positive response as 50% decrease in tumour growth vs vehicle control. Thus tumours can technically progress under therapy and still be considered as responders (indeed this occurs in 2/3 tumour growth curves presented in Figure 1). The growth curve for only one tumour treated with each aurora kinase inhibitor (MLN8054 and MLN8237) was presented, and the end point classification for all tumours is presented in the table. The actual tumour/patient shown in Figure 1a are not defined, thus it is not possible to cross compare with the table. The complete tumour growth/dose response curves for the remaining 17 patients should also be shown, as tumour growth rates and responses relative to time 0 (i.e. tumour regression) cannot be gauged from the presented data.*

Response: The growth curves of patient tumours V13 and V35 treated with MLN8054 and MLN8237, respectively, were shown in Fig. 1. Growth curves of other patient tumours are now shown in supplementary information Fig. S2 and S3.

Question: *2. Figure S2 suggests that there is no direct correlation between therapeutic*

responsiveness and AURKA inhibition - have the authors investigated possible mechanisms of non-responsiveness? Do non-responding tumours also display the enlarged cellular size and multi-nuclear characteristics of responding tumours?

Response: We defined 50% volume reduction compared with vehicle control as "responder". Almost all of the tumours responded to the treatment with decreased p-Aurora Kinase A, although some tumours did not show a 50% reduction. We feel the definition of responder and non-responder may be confusing to the readers. Since we have shown the growth curves for all patient tumours in the current manuscript and for the sake of clarity, we have opted to remove this table and avoid such a strict definition of responder and non-responder.
According to literature, when tumours acquire resistance to a drug they may develop gatekeeper mutations that block the ability to respond to the drug, or alternatively the drug target may continue to be inhibited, but alternative growth promoting pathways become activated, allowing tumour growth in the face of continued inhibition of drug target. The melanoma patients included in this study had received prior treatment before the surgical samples were obtained for this study. Therefore, it is likely that tumour cells with driver mutations, genetic deletions or amplifications may have be selected for during treatment with the Aurora kinase inhibitor that allowed growth of the tumour through the alternative pathway even though Aurora Kinase A continued to be inhibited.

Question: *Do the 'relapsed' tumours that are non-responsive to the second round of therapy display these senescent characteristics?*

Response: We performed H&E staining in the relapsed tumours V24 and V26 and found that in some areas, the relapsed tumours lost the morphological changes associated with senescence (Fig. S7). We had shown in the original manuscript that we treated these relapsed tumours (V23, V24 and V26) with MLN8054 again some mice with implanted tumour from the same patient responded while others did not: none of the V23 tumours responded to the second treatment; one V24 tumour did not respond whereas others responded; all V26 tumours responded. H&E staining demonstrated that all of the responding tumours display the large cellular size and multi-nucleated features whereas the non-responding tumours did not display these features.

Question: *Other than the morphological changes associated with senescence, do the authors have any data supporting the induction of senescence in vivo (for example SA-β-Gal positivity...this is shown for Hs294T, but not for any of the implants)?*

Response: We performed SA-β-Gal staining using V29 and V35 tumour implants, which exhibited a response to the aurora kinase inhibitor (Fig. 6A and Fig. S13). We also measured cytokine levels by cytokine array using V35, which showed up-regulation of cytokines, such as IL-6, IL-8 and GRO (Fig. 6C).

Question: *How can the authors rule out the possibility that the anti-tumour activity is due to an off target effect?*

Response: In the first manuscript describing MLN8054 (Manfredi, MG, PNAS 2007), the authors determined the inhibition of 226 kinases by MLN8054. Their data showed that MLN8054 is much more specific to aurora kinase A and aurora kinase B. They also compared MLN8054 with siAurora A and siAurora B. In our manuscript, we used both MLN8054 and MLN8237 and observed the same phenotype.

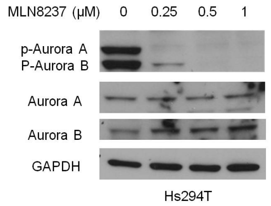

Although our data supported that MLN8237 is more specific to Aurora A than Aurora B, after 3 day-treatment, 0.5 μM MLN8237 inhibited both p-Aurora A and p-Aurora B (Fig. S1B). Therefore, we concluded that the phenotype we observed is due to the inhibition of both p-Aurora A and p-Aurora B. In the title, we emphasize that we are "Targeting Aurora Kinases". In addition, when we increased MLN8237 concentration to 50 μM, we still observed the same phenotype.

Question: *3. The authors mention that durable responses can be induced following short term Aurora kinase inhibitor treatment without the generation of secondary tumours, yet the authors present no evidence that untreated or vehicle-treated tumour implants are capable of secondary tumour formation.*

Response: When we refer to secondary tumour formation we meant drug-induced spontaneous tumour. We have revised the manuscript for clarity. To provide solid evidence, we treated aged FVB mice (22 MLN8237 and 16 vehicle control mice) for 4 months. Different organs were examined by a pathologist
(Table S1). Nothing unusual for an aged FVB population was noted.

Question: *4.The authors imply that immune infiltration is due to the SASP but provide little data in support of this notion. Nor do the authors provide data suggesting that neutrophils or macrophages contribute to the anti-tumour effect of MLN8237 (i.e. by antibody-based neutrophil depletion).*

Response: In addition to IHC, we analysed neutrophil and macrophage infiltration in patient tumours using FACS. We wanted to analyse patient tumour V35 to keep consistent. Unfortunately, some V35 tumours disappeared after treatment. Others are small, which were used to analyse *in vivo* senescence, DNA damage, and tissue cytokine array. Therefore, we analysed V32 and our results revealed a considerable increase in Ly-6G and F4/80 cells in the tumour implant in the mice treated with MLN8237 as compared to vehicle control. Six pairs of tumours were analysed and one representative graph is shown (Fig. 6D). In the MLN8237 treated patient tumours, Ly-6G positive cells was increased from 1.96% to 4.77% and F4/80 positive cells were increased from 1.0% to 21% compared with vehicle treated tumours. In additional experiments described in the revised manuscript, we depleted macrophages in immunocompetent mice and injected senescent mouse melanoma cells into these mice. Our data show that tumours arose in macrophage-depleted mice much earlier than in mice where macrophages were not depleted (Fig. 7).

Question: *Did co-treatment with MLN and BMS affect immune infiltration in vivo or any of the in vivo biomarkers for senescence, the SASP or Aurora kinase inhibition?*

Response: When we combined BMS345541 and MLN8237, we lost the characteristics of enlarged cellular size and multi-nuclei (Fig. 9B). We also demonstrated a reduction in cytokine secretion *in vitro*, indicating that SASP was inhibited (Fig. 8C).

Question: *Also, the quantification does not agree with the images shown and the numbers don't make sense - there are only a few infiltrating cells. If the quantification is indeed correct, it seems that the IHC images are not representative.*

Response: The quantification of neutrophil and macrophage infiltration was removed from Fig. 5E and 5F. We analysed neutrophil and macrophage infiltration in V32 after treatment using FACS (Fig. 6D).

Question: *5. In figure 3H, I cannot see any difference in SA-β-Gal positivity with siATM, siChk2 or siATM+siChk2 in Hs294T, thus it is difficult to accept the conclusion that senescence is dependent upon ATM or Chk2 in these cells. Quantification of the percentage of SA-β-Gal positive cells would be helpful.*

Response: The results were derived from the quantification of β-Gal positive cells from 3 separate experiments as indicated in the figure legend. Images in Fig. 3H are representative.

Question: *Similarly, I don't see a difference between H2AX staining in control and MLN-treated cells (Figure 5). Quantification is required (% nuclei with foci).*

Response: We agree that this figure lacked clarity and additional experimentation showed that 53BP1 is a better DNA damage marker in this case. We stained 53BP1 both *in vitro* and *in vivo. An a*pparent change was observed (Fig. 3B, 5B and 6B).

Question: *6.The authors claim that there is no synergistic effect between MLN and BMS, but what happens if sub-optimal doses of drug are used? In addition, if NFκB inhibition bypasses the senescence response, would one expect tumour outgrowth rather than synergistic inhibition?*

Response: When we used 100 mg/kg of BMS, we did not observe a synergistic effect between MLN and BMS (Fig. 9A and 9C). However, when we used 75 mg/kg of BMS once daily, the combined treatment resulted in less inhibition of tumour growth than either single treatment alone (Fig. 9D).

Question: *The DNA content analyses and inhibition of senescence support the idea that cells have re-entered the cell cycle, but is not really reflected in Fig 6F or 6G. Is NFκB is expressed in the recipient stroma? Is it possible to inhibit NFκB in a tumour cell-autonomous manner (through transduction of Hs294T with shRNAs)? Why are single time points shown and not growth curves? Again, the patient xenograft ID is not defined.*

Response: In Fig. 8D, we treated cells for 2 days, but in Fig. 8F (6F in the original manuscript), we treated cells for 5 days. Five-day treatment with BMS *in vitro* can induce apoptosis. Therefore, if we treat for 5 days, we cannot analyse DNA content. When we lowered the dose of BMS *in vivo*, we can see tumour cells re-entered the cell cycle (Fig. 9D).

According to the literature, senescence of stromal fibroblasts is related to production of the chemokine GRO-α (Yang, G PNAS 2006), which can be regulated by NF-κB.

IKKβ knockdown cell line was generated using shRNA. MLN8237-induced senescence in these cells was impaired.

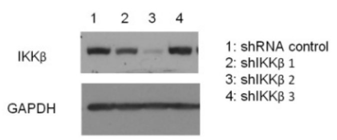

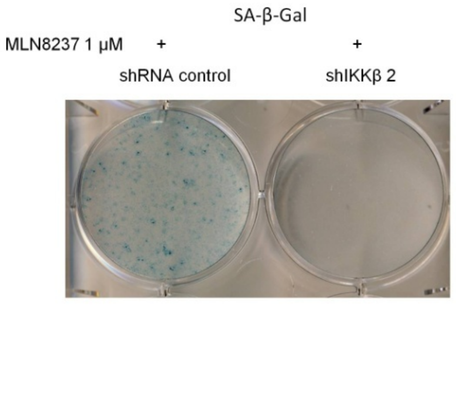

Question: *Why are single time points shown and not growth curves? Again, the patient xenograft ID is not defined.*

Response: The full growth curves are shown in Fig. 9A, 9C, and 9D. Patient ID is V32.

Question: *7.The authors should improve their discussion of the literature. Previously published studies of interplay between aurora kinase A and NFκB signalling were not discussed or mentioned. The findings of the present study oppose those of Chefetz et al (Cell Cycle 2011), who showed in epithelial ovarian cancer stem cells that Aurora Kinase A inhibition (using MK-5108) induces polyploidy cell cycle arrest, with decreased NFκB activity, decreased cytokine production and accumulation of IB1. The study by Chefetz et al was not discussed in the present manuscript. Similarly, Aurora Kinase A has been shown to directly phosphorylate I;. (Briassouli et al, Cancer Res. 2007), yet Liu et al. show degradation of I ; with AKi treatment. Liu et al. must be careful to avoid overstating the generality of their findings or extensively extrapolating the findings of others to their study.*

Response: Related articles are now cited and discussed. Our results do not disagree with the results of Chefetz et al. In our model (Fig. 9E), we demonstrated that activation of NF-κB is via the MLN8237-induced DNA damage response. Previous publications demonstrated that ATM can activate NF-κB in response to DNA damage (Li, N, JBC 2001 and Wu, ZH, Science 2006). We believe that although inhibition of Aurora A can under some circumstances down-regulate NF-κB, activation of ATM/Chk2 can overcome this down-regulation of NF-κB. We discussed these data on page 17 in the revised manuscript.

Minor points

Question: *9.Only one mouse for V18 and four mice for V24 are shown in Table 2. Where are the data for V18A1, 2, 4 or 5, and V24A5?*

Response: V24A5 died during treatment, which was noted in the revised manuscript on. We had 6 V18 tumour-bearing mice. We just kept one mouse because its tumour was too small to do other analysis. Other V18 tumour-bearing mice were sacrificed to generate tissue microarray. We have removed data from V18, which did not change any of our conclusions.

Question: *10. I do not agree with the authors' impact statement that their 'data demonstrate the contribution of the NFκB pathway to chemotherapy induced senescence and chemosensitivity', as inhibition of NfκB, despite inhibiting some senescence characteristics, did not affect the therapeutic outcome in the presented model. The authors state twice on 'the paper explained' page that therapy-induced senescence limits tumour growth and will not result in secondary tumours, yet produce no data demonstrating that their model is capable of secondary tumour formation.*

Response: We agree that this statement could have been misunderstood and have revised the impact statement accordingly. "IMPACT: Our findings suggest that therapy-induced senescence limits tumour growth. Induction of senescence represents a viable approach for cancer therapy."

In regards to the question of secondary tumour formation, we communicated with reviewer 1 through Dr. Funk for definition of the phrase "secondary tumour". When we refer to secondary tumour, we referred to drug-induced spontaneous tumour formation. We have revised the manuscript to reflect this definition, accordingly.

Question: *11. Some of the experiments are missing controls or could present the data more clearly.*
- Figure 2E - Authors need to label the cell lines either above or below the western blots. It's insufficient for readers/reviewers to assume an order. Please indicate p63 and p73 bands (or size

markers) on the western blots.
- Figure 3: Authors need to include 'total' Chk2 and Chk1 on western blots. (3C,D and G)
- Figure 4: GM-CSF is not indicated on the cytokine array.
- Figure 5: I cannot see any difference in γ H2AX staining between control and MLN treated samples. Authors please provide quantification - i.e. number of nuclei with foci.
- Figure 6: need a 'total' p65 control blot.

Response: Figure 2E: cell lines are now labelled. The size markers of p63 and p73 were added. Figure3: Total Chk2 and Chk1 were added. Figure 4: GM-CSF was labelled. Figure 5: A new marker 53BP1 was used. Figure 6: Total p65 was added.

Referee #2:

Mice with ectopically transplanted human melanomas were treated with the Aurora A inhibitors MLN8054 and MLN8237. Interestingly, 8 out of 15 tumours (derived from in total 4 patients) responded to the treatment, while 7/15 relapsed. Further studies with cell line-induced tumours showed that MLN treatment induced senescence driven by the ATM/Chk2 pathway. This is all very interesting and well-performed work. But then the authors take a big leap in concluding that the reduction in tumour growth is a consequence of NF-kB induced senescence. In fact, this is the main conclusion (as reflected by the title) of the manuscript.

Response: We recognize that we did not make our conclusions clear. We did not mean the senescence is induced by NF-kB. The senescence is induced by polyploidy and DNA damage. But inhibition of NF-kB can impair senescence. We have revised the title from **"Targeting Aurora Kinase Limits Tumor Growth through Induction of NF-κB-Associated Senescence"** to "**Targeting Aurora Kinases Limits Tumor Growth through DNA Damage Mediated Senescence and NF-κB Inhibition Impairs Senescence"** and believe the text more accurately represents our conclusions.

My major problem is that this conclusion is not substantiated by the data shown in figure 6. While inhibition of NF-kB by BMS inhibits the typical senescent features (b-galactosidase activity, SASP) induced by MLN treatment, BMS treatment itself causes a massive reduction in cell number and tumour shrinkage and it is therefore impossible to conclude if the effect of MLN treatment on tumour growth is mediated via NF-kB induced senescence. The authors should be more precise and state that their data show correlations.

Response: We repeated the combined treatment with a lower dose of BMS, which impaired the outcome of the therapy (Fig. 9D). We have revised the title accordingly to better reflect our conclusions. We are not stating that NF-kB induces senescence—but that "Taken together, NF-κB activation is correlated with DNA damage-induced senescence." This is now more clearly discussed on page18 of the revised manuscript.

Other points:

Question: *On page 6 the authors conclude that the levels of Aurora A and B are significantly higher in melanoma cell line than in normal melanocytes (Suppl fig 1). Without information on the mitotic index of the treated cells this conclusion cannot be drawn. If the melanoma cell lines cycle faster than the NHEM cells, more cells will accumulate in mitosis after 16h treatment with nocodazole. Expression of Aurora A and B is highest in mitosis and the high Aurora levels in the tumour cell lines could thus be explained by a higher number of mitotic cells. The authors should therefore collect mitotic cells by mitotic shake-off and prepare cell extract thereof. In line with this, to what*

extend are the Aurora levels in the cell lines representative of the levels in the patient-derived tumours transplanted into the mice?

Response: We have revised the figure legend for clarity. We treated cells with nocodazole for 16 hrs and collected cells by mitotic shake-off. Data show that melanoma cells synchronized in this fashion and collected by mitotic shake off have elevated levels of aurora kinases compared to normal melanocytes. Previous studies have shown melanoma patient tumours have high levels of p-Aurora A and p-Aurora B (Wang X, Genes &Cancer 2010). We were curious whether they over-express Aurora A and B. Since we cannot synchronize patient tumours, we used melanoma cell lines. Our data cannot conclude that patient-derived tumours overexpress Aurora A and Aurora B, but they are supportive of the work of Wang et al. and lead us to propose that melanoma tumours might respond to aurora kinase inhibitors.

Question: *Based on the IF pictures presented in figure 1C I am unable to appreciate the effect of the MLN drugs on pAurA or the mitotic spindles.*
Response: Resolution and size were modified (Fig. 1D) to better visualize the data here.

Question: *Page 7: ....'exhibited greatly enlarged cellular size and cells were often multi-nucleated, which are both characteristic of senescence'.* .
The authors should be aware that these are also characteristics of failed cell division.
Response: We agree and have deleted this sentence.

Question: *7/15 tumours relapsed after drug administration, but only 3 of the 7were tested in a second regime of MLN8054. To conclude anything on acquired drug-resistance all 7 tumours should have been tested.*

Response: We agree with the reviewer that drug-resistance will require a full and more extensive study. As such, it falls outside the scope of this manuscript. However, detailed studies on the mechanism of resistance to MLN8237 are on-going and will be the focus of a future manuscript. For the purpose of the study described herein, we kept some surviving mice and retreated with drug to monitor the long term effects of the drug. We observed both resistant and sensitive phenotypes. One question we wanted to address is whether relapsed tumours still display the enlarged cellular size and multi-nucleated characteristics of the responsive tumours (Fig. S7 and S8). We found that the relapsed tumours displayed regions of enlarged cellular size and multi-nucleated characteristics, but overall had lost these characteristics.

Question: *In figure 2B the authors show that the apoptotic index of SK-Mel2 cells increases from approx. 1% to 25% after 72 h of treatment with MLN. Yet, on page 8 they conclude that apoptosis was not significantly increased. This may be true for the other cell lines but not for SK-Mel2.*

Response: We revised the statement thus, "As apoptosis was not significantly increased in most melanoma cell lines, we predicted that other processes were responsible for reduced tumour growth in response to drug treatment."

Question: *I miss the point of the TNFalpha experiment (fig. 7). To me it shows that two cell lines treated with MLN are responsive to TNFalpha treatment. I fail to see how this can be used to conclude that "induction of IKKb/NK-kB reinforces drug-induced senescence".*

Response: We agree that these data were confusion so this figure has been removed, as it neither supports or detracts from our overall conclusions.

Question: *Page 15: "These findings provide solid evidence that induction of senescence in tumours are highly resistant to apoptosis, as in melanoma, can limit tumour growth". I find this a puzzling remark since on page 12 it is mentioned that BMS induces apoptosis in melanoma cells.*

Response: We have revised this statement for clarity. "These findings provide solid evidence that induction of senescence in melanoma can limit tumour growth". "

Referee #3:
Major points:

Question: *1. What are the reasons for acquired drug resistance, e.g. in tumour V23P3A? Data or at least a discussion regarding this point is needed.*

Response: We have performed RNA deep sequencing of the resistant tumours. Many DNA mutations (more than 100) were found, which may be due to DNA damage. Because we haven't identified mutations responsible for the drug resistance, we didn't add these data to the manuscript. Studies related to resistance are on-going and will be the focus of another manuscript. However, we discuss this briefly in the revised manuscript on page 16.

Question: *2.The authors describe higher leucocyte infiltration in senescent melanoma xenografts but only a "steady state" level of tumour size is observed and no tumour remissions. Do the leucocytes indeed contribute to long term tumour control? How do the growth curves look when leukocyte function is inhibited, e.g. via antibodies? How do the authors explain that there are no real tumour remissions induced by the infiltrating immune cells?*

Response: Since CD4+ T cells are required for senescence surveillance (Kang, TW, Nature, 2011) and our experiments were performed in nude mice (without T cells), we observed stagnation of tumour growth, without remission. When we depleted macrophages from immunocompetent mice and implanted senescent mouse melanoma cells, we observed more tumours formed in macrophage-depleted mice compared to mice with macrophages (Fig 7), suggesting that macrophages can clear senescent cells. However, when the senescent cells are not cleared by macrophages, tumours grow more rapidly. These data show that participation of SASP recruitment of macrophages is important to the tumour response to MLN8237.

Question: *A study by Scott Lowe and colleagues has shown that senescence induction by p53 reactivation led to rapid tumour clearance via different innate immune cells.*

Response: In their model, senescence is dependent on p53. In our model, senescence is independent of p53 (Fig. 2E). We discussed this in the revised manuscript on page 17.

Question: *Is there a distinct secretory phenotype of senescent melanoma cells in tumours treated with Aurka inhibitors? As all studies were done in nude mice it is also conceivable that the adaptive immune system might be needed (see Kang et al., Nature 2010) to get full immune responses against senescent melanoma cells after Aurk inhibition. Obviously, studies in immunocompetent mice can't be conducted with primary human melanomas, however a discussion regarding this point would help.*

Response: We measured cytokine levels by cytokine array using patient tumour V35 (Fig. 6C). We agree with the reviewer that the adaptive immune system, particularly Tcells, are required for effective immune surveillance. We have discussed this in the revised manuscript on page 16 and 17.

Question: *3.The authors state "These results suggest that p53, p21, and p16 are not essential regulators of drug-induced senescence". As only Aurk inhibition was tested the statement should be relativized.*

Response: We have revised this statement as suggested on page 9. “The results show that blocking p53 did not alter drug-induced senescence in Hs294T or SK-Mel-28 cells (Figure 2F), indicating that p53 is not required for MLN8237-induced senescence.”

Minor points:

Question: *1.The introduction to the manuscript is somehow unidirectional, in particular regarding the SASP. It is well established that the SASP can also have antitumourigenic effects, for example as shown by work from Scott Lowe and others (Xue et al., Nature 2007, Kang et al., Nature 2011).*

Response: We have re-organized the introduction to reflect this.

Question: *2.It is mentioned that a recent study by Kang et al showed that senescent cells are cleared by macrophages. My understanding is that this study showed that precancerous senescent hepatocytes are cleared through a CD4 T-cell response that is dependent on macrophages as effector cells. This should be specified. Along the lines the sentence "We predict that the recruitment of immune infiltrates in response to inflammatory cytokines and chemokines aids the removal of senescent tumour cells in vivo (Kang et al, 2011)" could be misleading for the reader, as the indicated study by Kang et al showed indeed already that secreted factors from senescent hepatocytes recruit immune cells which then clear senescent hepatocytes. The manuscript would benefit from a more precise discussion of the literature.*

Response: We have revised the discussion for clarity and with emphasis on the subject of immune clearance of senescent cells on page 16 and 17.
“A recent study showed that immune surveillance of senescent pre-malignant hepatocytes limits tumour formation mediated by macrophages and T cells (Kang et al, 2011). Previous studies also demonstrated that the immune response contributes to the induction of senescence or the clearance of senescent tumour cells to limit tumour growth (Rakhra et al, 2010; Xue et al, 2007). We have demonstrated here that senescent melanoma cells secrete IL-6, IL-8 [CXCL8], GRO, and GRO-α [CXCL1]) *in vitro* and *in vivo*, which is associated with the pro-inflammatory response and recruitment of neutrophils and macrophages to senescent tumour cells. According to the premalignant model by Xue et al, we expected that the recruitment of immune infiltrates in response to inflammatory cytokines and chemokines aids the removal of senescent tumour cells induced by the drug *in vivo*. However, in this model, we did not observe significant tumour regression in most tumour-bearing nude mice probably because we used immunodeficient mice. Therefore, we could not investigate the contribution of innate immune cells to senescence. To address this issue, we depleted macrophages in immunocompetent mice and then injected senescent mouse melanoma cells. More tumours developed in macrophage-depleted mice compared to mice that retained macrophages.”

Question: *3. Fig. 1C - better resolution is needed*
4. Fig. 1E - define the magnification (please provide figures with the same magnification), show multinuclei with arrows
5. Fig. 2E- label names of cell lines in the western blot
6. Fig. 3B - define MLN treatment, similar to fig.5B
7. Fig. 4A - GM-CSF is not defined on the array
8. Fig. 4D - number of days specified in the figure differs from information in the text
9. Fig. 5E -please specify the used marker for neutrophil staining

10. Information regarding figures: 2A, 3A, 3F-G; 4C, 6A, 6D, 6F, 7B is missing in the material and methods part.

Response: Fig 1D (Fig 1C in the original manuscript): A better resolution image was added. Multinucleated cells are shown.
Fig 1E: magnification is shown.
Fig 2E: Names of cell lines were labelled.
Fig. 3B: The treatment was defined in the figure legend.
Fig. 4A: GM-CSF was labelled.
Fig. 4D: number of days was changed.
Fig. 5E: The marker is Ly-6G/C.
The information regarding figures: 2A, 3A, 3F-G; 4C, 6A, 6D, 6F, 7B was added to the material and methods.

We are submitting the revised manuscript "Targeting Aurora Kinases Limits Tumor Growth through DNA Damage Mediated Senescence and NF-κB Inhibition Impairs Senescence" for consideration for publication in the EMBO Molecular Medicine. The co-authors and I believe we have appropriately addressed the concerns of the reviewers and revised the manuscript accordingly.

Again, we would like to thank the reviewers for their constructive criticisms, which have enabled us to generate a manuscript which more clearly supports our conclusions.

2nd Editorial Decision 22 August 2012

Thank you for the submission of your manuscript "Targeting Aurora Kinases Limits Tumor Growth through DNA Damage Mediated Senescence and NF- B Inhibition Impairs Senescence" to EMBO Molecular Medicine. We have now heard back from the referees whom we asked to re-evaluate your revised manuscript.

As you will see, the reviewers acknowledge that the manuscript was significantly improved during revision. However, while reviewer #2 indicates that that the manuscript is suitable for publication, reviewer #1 raises issues that should be convincingly addressed. Since we do acknowledge the potential interest of your findings, we would therefore be open to allow a second revision of the manuscript that would address the outstanding issues.

Importantly, reviewer #1 points out that data in Figure 7 should be improved and that a number of inconsistencies in presentation should be addressed.

Revised manuscripts should be submitted within three months of a request for revision; they will otherwise be treated as new submissions, unless arranged otherwise with the editor.

I look forward to seeing a revised form of your manuscript as soon as possible.

Yours sincerely,

Editor
EMBO Molecular Medicine

***** Reviewer's comments *****

Referee #1:

Summary:
Liu et al demonstrate that the Aurora kinase inhibitors induce a senescence-like response in melanoma cell lines and xenograft (implant) models in vitro and in vivo. The authors show that the Aurora-kinase inhibitor-mediated senescence can occur independently of p53, is associated with ATM/Chk2 pathway activation, NF B signaling and induction of a SASP. Further the authors show that components of the innate immune system (neutrophils and macrophages) are recruited to senescent tumors following Aurora kinase inhibitor treatment.

The revised manuscript is an improvement from the original submission, in that appropriate controls for some figures have been included, the images are generally more interpretable and in vivo data supporting the induction of senescence by Aurora-kinase inhibition has been included. Discussion of the literature has improved, and the overall clarity of the manuscript is improved. However, errors and inconsistencies throughout the figures remain substantial. Moreover, a number of the figure panels have changed between the original and revised manuscripts without explanation, raising concerns that the authors are being selective with the data they present. In addition, there are spelling and grammatical errors throughout the text (too numerous to list below).

Specific comments:
1. Support for the induction of senescence and immune surveillance in vivo has improved in the revised manuscript. The experimental xenograft system is of interest, however the general findings (therapy-induced senescence, NF B-dependent SASP, immune surveillance etc) are already known and have been published elsewhere.

2. There remain problems with the figures. Some examples:
(1) Inconsistencies in figure legends and figure titles, predominantly with respect to the patient/tumor IDs. For example Figs S5 (V09 in the title vs V29 in the legend) and S6 (V19 in the title vs V29 in the legend).
(2) Incorrect citation of literature, for example, the authors cite Sugimoto et al 2002 for the xenograft model, yet Sugimoto et al do not perform or describe any xenograft experiments.
(3) Inconsistency in scale bars, for example in Figure 1 the H&E panels have 10um scale bars, whereas the IF and Ki67 panels have 50um bars. The magnification appears to be the same.
In other figures, the scale bars have changed considerably (previous scale bars of 10um in the original submission have been replaced with scale bars of 100um in the revised manuscript, however the figures in both versions appear to have the same magnification).
(4) In Figure 3E, the SKMel-28 MLN1uM panel has changed from color to black and white.
(5) In figure 3H the authors stated that the panels in the original submission were representative, yet the images shown in the revised version of the manuscript are new. In figures where the quantification and images didn't match up the authors have chosen to remove the quantification.
(6) Figure 7B suggests that activated macrophages are capable of decreasing the viability of senescent MelA cells. This figure also suggests that the SASP is not sufficient to activate RAW macrophages. Targeting of MelA cells by RAW cells may not have anything to do with senescence or the SASP.
Figure 7C suggests that depletion of macrophages can enable tumor formation by MelA cells. Firstly, the fact that MLN-treated MelA form tumors suggests that not all of the cells were senescent (if in fact senescence is an irreversible process). Secondly, the authors do not present the appropriate positive controls to demonstrate that untreated MelA cells for tumors in immunocompetent or macrophage depleted mice. Also, I'm unsure as to why the authors changed the tumor system for this experiment. Nude mice have an intact innate immune system (including macrophages), thus macrophage depletion experiments could have been performed in the xenograft model.
(6) The data in Table S1 do not appear to relate to any aspect of the senescence study. The fact that 6/22 MLN8237 mice developed some kind of spontaneous tumor or hyperplasia, whereas only 1/16 of the vehicle treated mice developed hyperplasia suggests that MLN8237 may influence tumorigenesis. Is this significant? - The authors state in the rebuttal that 'nothing unusual for an aged FVB population was noted'...why then, are many more cases of hyperplasia noted in the MLN treated mice?
(7) Figure S7 - How did the authors decide whether tumor regions were growing or senescent? I

could not see any obvious difference between regions indicated by blue vs black arrows in the two panels.
(8) Figure S9 - patient/tumor is not defined
(9) Figure S12 - patient/tumor is not defined
(10) Figure S14 - is ambiguous. It is difficult to tell whether there is no -gal staining of whether there are simply no cells in the shIKK 2 well. From the data presented, there is no way to know whether shIKK kills/arrests cells in the presence or absence of MLN8237. The authors mention in the rebuttal that BMS treatment for 5 days induces apoptosis in vitro, thus it would not be surprising if shIKK kills cells.
(11) The tumor growth curve for V13 is duplicated in figure 1 and fig.S2.
(12) Patients V23 and V32 are missing from fig.S4 despite the growth curves being shown for these tumors in S2 and S3.
(13) In text (p7) the authors make reference to the fact that non-responding V23 tumors do not display the senescent phenotype, yet they have removed all previous references to a senescent phenotype in their model (the senescent phenotype is therefore completely undefined).

Referee #2:

The authors have greatly improved the manuscript based on the suggestions by the three referees. The manuscript reads much better and the conclusions are now valid. Moreover, the resolution and quality of the various IF images in the figures have also been improved. In my opinion this new version of the manuscript is now suitable for publication in EMBO Molecular Medicine.

2nd Revision - authors' response 08 October 2012

Thank you very much for reviewing our manuscript entitled "Targeting Aurora Kinases Limits Tumor Growth through DNA Damage-Mediated Senescence and Blockade of NF-κB Impairs this Drug Induced Senescence". We appreciate the reviewer's suggestions and comments. We are pleased that Reviewer 2 was satisfied with our revisions, and in response to the comments from reviewer 1 we have completed a series of *in vitro* and *in vivo* studies to better support our conclusions. We have also carefully edited the manuscript and removed any errors and added additional clarification throughout. Please also note the small change in the title with the addition of "this Drug Induced" for clarification and qualification.

Specific comments from Reviewer 1:

***Comment 1**. Support for the induction of senescence and immune surveillance in vivo has improved in the revised manuscript. The experimental xenograft system is of interest, however the general findings (therapy-induced senescence, NF-κB-dependent SASP, immune surveillance, etc) are already known and have been published elsewhere.*

Response: We agree that therapy-induced senescence, NFκB-dependent SASP, and immune surveillance of tumours have been explored in prior publications in other models. However, most of these results were published using either genetic mouse models or using models to investigate the role of senescence in tumour progression, such as the report from the Campisi lab that senescent fibroblasts promote tumour growth (Krtolica, et al, 2001, PNAS). The strength of our manuscript is that we investigate drug induced senescence of tumour cells using clinical samples and provide key data that will impact on-going clinical trials using MLN8237 for treatment of melanoma. Our results suggest that while systemic treatment with MLN8237 induced senescence and reduced tumour growth, there was not significant induction of apoptosis or tumour regression and combined use of MLN8237 with inhibition of IKKβ did not provide advantage. Moreover, we investigated potential tumour promoting effects on normal tissues in aging mice with continuous *in vivo* delivery of MLN8237.

***Comment 2.** There remain problems with the figures. Some examples: (1) Inconsistencies in figure*

legends and figure titles, predominantly with respect to the patient/tumour IDs. For example, Figs S5 (V09 in the title vs V29 in the legend) and S6 (V19 in the title vs V29 in the legend).

Response: We have corrected the errors in the figure legends. In Figure S5, the tumour ID is V09. In Figure S6, the tumour is V19. We have carefully reviewed all of the tumour identifications in the revised manuscript and made corrections in titles and legends.

Comment 3: *Incorrect citation of literature, for example, the authors cite Sugimoto et al 2002 for the xenograft model, yet Sugimoto et al do not perform or describe any xenograft experiments.*

Response: We have corrected this citation error and double checked all the references. They are all correctly cited.

Comment 4: *Inconsistency in scale bars, for example in Figure 1 the H&E panels have 10um scale bars, whereas the IF and Ki67 panels have 50um bars. The magnification appears to be the same. In other figures, the scale bars have changed considerably (previous scale bars of 10um in the original submission have been replaced with scale bars of 100um in the revised manuscript. However the figures in both versions appear to have the same magnification).*

Response: We apologize for this confusion. We have noted that the software associated with our new microscope does not automatically change the scale bar when changing the objective and we had to manually make corrections. These corrections were included in the first revision of the manuscript. However, we somehow missed Figure 1, which was supposed to show a scale bar of 50µM, like the others. All scale bars were double checked and corrected in this second revision of the manuscript.

Comment 5: *In Figure 3E, the SKMel-28 MLN1uM panel has changed from colour to black and white.*
Response: We appreciate this point. We have corrected Figure 3E, which is now in colour.

Comment 6: *In figure 3H the authors stated that the panels in the original submission were representative, yet the images shown in the revised version of the manuscript are new. In figures where the quantification and images didn't match up the authors have chosen to remove the quantification.*

Response: To better clarify the issues raised by Reviewer One in the initial review, we repeated this experiment and performed double transfection to improve the transfection efficiency. We quantitated the results from three independent experiments and these data were shown in Figure 3H. Regarding the second point, in Figure 5 (now Figure 6D), we removed E and F because the reviewer was not satisfied that we were showing sufficient resolution and quantitation to clearly show a difference in leukocyte infiltration into the tumour. Instead, in a new series of experiments, we utilized FACS analysis to accurately quantitate the macrophages and neutrophils that infiltrated into the tumours treated with vehicle control or MLN8237. Seven tumours from each group were analysed. The FACS data shown in Figure 6D support our conclusion that there was an increase in immune cells infiltrating the tumours with MLN8237 induced senescence. We have now replaced the dynamite plot with scatter plot to better visualize the data.

Comment 7: *Figure 7B suggests that activated macrophages are capable of decreasing the viability of senescent MelA cells. This figure also suggests that the SASP is not sufficient to activate RAW macrophages. Targeting of MelA cells by RAW cells may not have anything to do with senescence or the SASP.*

Response: Our intention was not to claim SASP can activate macrophages, but rather to show that SASP can contribute to the recruitment of immune cells into the senescent tumours. Since the reviewer thought RAW cells may be relevant to our *in vivo* model for studying immune cell recruitment in response to SASP, this result was removed.

Comment 8: *Figure 7C suggests that depletion of macrophages can enable tumour formation by MelA cells. Firstly, the fact that MLN-treated MelA cells form tumours suggests that not all of the cells were senescent (if in fact senescence is an irreversible process). Secondly, the authors do not present the appropriate positive controls to demonstrate that untreated MelA cells for tumours in immunocompetent or macrophage depleted mice. Also, I'm unsure as to why the authors changed the tumour system for this experiment. Nude mice have an intact innate immune system (including*

macrophages), thus macrophage depletion experiments could have been performed in the xenograft model.

Response: We agree with the reviewer that it is unlikely that all the MLN8237 treated cells were senescent. There appears to be a small population of cells that are not responding to MLN8237 with induction of senescence. However, we can see from β-gal staining (Figure 7A) that most of the MLN8237 treated cells were senescent. Moreover, the mean tumour volume of the MLN8237 pre-treated tumours was very small compared to the tumour volume associated with injection of non-senescent cells (Figure 7C). These data strongly support the concept that the majority of the MLN8237 treated cells were senescent and did not grow when placed into the mouse.

We did include the untreated control group when this experiment was initially performed, but due to the unexplained death of 2 of the untreated mice by the end of week one, and a third death during week two of the experiment, we did not include this group, though the two surviving mice did develop tumours. However, we have now repeated this entire experiment and have an appropriate number of vehicle treated controls (n=7) to compare to the MLN8237 treated group (n=5). Our data indicate that macrophages play an important anti-tumour role when tumours have a large percentage of senescent cells. In contrast, for the tumours formed from MLN8237-untreated and non-senescent tumour cells, clodronate removal of macrophages had little effect on tumour growth, possibly because fewer are recruited into the tumour (as shown if Figure 6D). Additionally, the macrophages that are recruited (though fewer in number than in MLN8237 treated mice), do not have a net positive or negative effect on tumour growth.

B

	mouse ID Liposome Control	Non-senescent Tumor left	right	mouse ID macrophage depleted	Non-senescent Tumor left	right	mouse ID Liposome Control	Senescent Tumor left	right	mouse ID macrophage depleted	Senescent Tumor left	right
8 days	1	+	+	1	+	+	1	-	-	1	+	-
	2	+	+	2	+	+	2	-	-	2	-	+
	3	+	+	3	+	+	3	+	-	3	+	+
	4	+	+	4	+	+	4	-	-	4	+	-
	5	+	+	5	+	+	5	-	-	5	+	-
	6	+	+	6	+	+						
	7	+	+	7	+	+						
17 days	1	+	+	1	+	+	1	-	+	1 (died)	NA	NA
	2	+	+	2	+	+	2	-	-	2 (died)	NA	NA
	3	+	+	3	+	+	3	+	-	3	+	+
	4	+	+	4	+	+	4	+	-	4	+	+
	5	+	+	5	+	+	5	-	-	5	+	-
	6	+	+	6	+	+						
	7	+	+	7	+	+						

+ = tumor detected

C

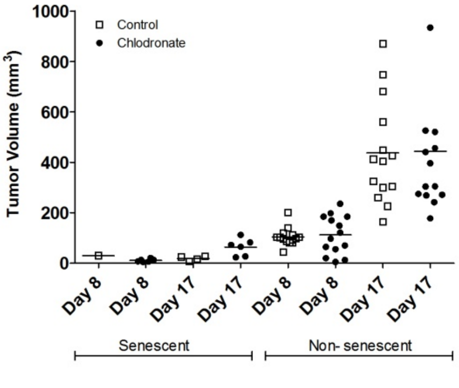

Regarding the rationale for using wild type C57BL/6 in this experiment instead of nude mice, reviewer 3 had mentioned that "As all studies were done in nude mice it is also conceivable that the adaptive immune system might be needed (see Kang et al., Nature 2010) to get full immune responses against senescent melanoma cells after Aurk inhibition. Obviously, studies in immunocompetent mice cannot be conducted with primary human melanoma, however a discussion regarding this point would help." Since T cells may be required to assist macrophages to destroy senescent tumour cells (Kang et al, 2010) and since nude mice have impaired T cells and in response to these comments from reviewer 3, we chose to use C57BL/6 wild type mice and mouse melanoma cells to evaluate the role of macrophages on tumour growth when senescence has been induced in

tumour cells by treatment with MLN8237. This rationale is discussed on page 13 of the revised manuscript.

Comment 9: *The data in Table S1 do not appear to relate to any aspect of the senescence study. The fact that 6/22 MLN8237 mice developed some kind of spontaneous tumour or hyperplasia, whereas only 1/16 of the vehicle treated mice developed hyperplasia suggests that MLN8237 may influence tumorigenesis. Is this significant? - The authors state in the rebuttal that 'nothing unusual for an aged FVB population was noted'...why then, are many more cases of hyperplasia noted in the MLN treated mice?*

Response: The rational for this experiment was based upon our concern that AURKA inhibition and subsequent induction of polyploidy, DNA damage and senescence may induce tumour in aging mice. In response to the reviewer's question, we requested an independent evaluation from a trusted statistician and we now include these analyses in our results on page 7 of the revised manuscript. When aging mice were treated for a prolonged period (4 months), we found tumour in the lung in only 2/22 MLN8237 treated mice and no spontaneous tumours were observed in the control group ($p=0.499$, Fisher's Exact Test). Liver hyperplasia was observed in 3/22 treated mice and 1/16 control mice ($p=0.625$, Fisher's Exact Test), while colon hyperplasia was present in 1/22 drug treated mice but not in the control group (p.0.99, Fisher's Exact Test) (Table S1). These non-significant p-values are not evidence of no effects of MLN8237 on spontaneous tumour formation, but suggest the effect is very small, requiring a much larger sample size to detect the true effect. Our data do suggest that secondary tumour formation should be evaluated with prolonged continuous treatment with MLN8237 in the on-going clinical trials. This revised statement and analysis are included on page 7 of the revised manuscript.

Comment 10: *Figure S7 - How did the authors decide whether tumour regions were growing or senescent? I could not see any obvious difference between regions indicated by blue vs. black arrows in the two panels.*

Response: In Fig. S7, we provided H&E staining of vehicle or MLN8054 treated tumours and relapsed tumours. We can see the morphology of relapsed tumour is similar to the vehicle treated tumour. These areas are now identified by a red circle on the figure.

Comment 11: *Figure S9 - patient/tumour is not defined*

Response: Tumour ID is V35. This is now stated in the figure and in the figure legend.

Comment 12: *Figure S12 - patient/tumour is not defined*

Response: We provided full images of Figure 5B and in Figure S12 which shows the Hs294T xenograft. This is now clearly stated in the figure legend.

Comment 13: *Figure S14 - is ambiguous. It is difficult to tell whether there is no β-gal staining of whether there are simply no cells in the shIKKβ well. From the data presented, there is no way to know whether shIKKβ kills/arrests cells in the presence or absence of MLN8237. The authors mention in the rebuttal that BMS treatment for 5 days induces apoptosis in vitro, thus it would not be surprising if shIKKβ kills cells.*

Response: The reviewer is correct that sh IKKβ knock down cells are not healthy. In this experiment, we infected Hs294T cells with lentivirus to knockdown IKKβ, before they were treated with MLN8237. Since some apoptosis is induced when IKKβ is knocked down, the reduced β-gal staining is not independent of the reduced number of viable cells. Enlarged photomicrographs are provided in the lower panel of Figure S14B showing that while about the same number of cells are present in the plate, many of the cells in the shIKKβ plate do not appear healthy and do not stain strongly for β-gal.

Comment 14: *The tumour growth curve for V13 is duplicated in figure 1 and fig.S2.*

Response: To avoid this duplication, V13 was removed from Figure S2. It was placed there initially to ensure the whole data set was together in Figure S2.

Comment 15: *Patients V23 and V32 are missing from fig.S4 despite the growth curves being shown for these tumours in S2 and S3.*

Response: We constructed tissue microarray (TMA) for each 19 patient tumour. However, vehicle treated V23 was highly necrotic and V32 was strongly pigmented, which made it impossible to analyse the results from the immunofluorescence experiments from these two tumours. This is stated on page 6 of the revised manuscript.

Comment 16: *In text (p7) the authors make reference to the fact that non-responding V23 tumours do not display the senescent phenotype, yet they have removed all previous references to a senescent phenotype in their model (the senescent phenotype is therefore completely undefined).*

Response: We apologize for not clarifying our point. We now provide a description of the "senescent phenotype" on page 3 of the revised manuscript. When we treated V23 for a second round of treatment, this tumour did not respond. The H&E staining showed that after the second round of treatment this tumour did not show senescence associated phenotype, such as the significant morphology change and multi-nucleus, compared to V26, which responded to the second round of treatment (Figure S8). We rewrote this sentence. "Tumors from patient V23 that did not respond to the second round of treatment did not display the enlarged flattened cellular morphology associated with the senescent phenotype (Figure S8A, S8C), whereas tumours from patient V26 responded and exhibited the enlarged flattened cellular morphology associated with the senescent phenotype based upon H&E staining (Figure S8B) . One of five V24 patient tumours did not respond to an additional round of treatment (Figure S8C), while the other four responded to treatment (Figure S8D). These data suggest that a second round of treatment may be useful for some patients and when tumours respond to the treatment, they display features associated with senescence, i.e. an enlarged flattened cellular morphology. This information is on page 8 of the revised manuscript.

We appreciate the comments from the reviewer and believe that our manuscript has been strengthened by the careful review. We hope that you will find this revised manuscript acceptable for publication in *EMBO Molecular Medicine*.
