# Supplementary material for: Targeting aurora kinases limits tumour growth through DNA damage-mediated senescence and blockade of NF-κB impairs this drug-induced senescence
Source: EMBO Mol Med. 2012 Nov 25;5(1):149–66. doi: 10.1002/emmm.201201378 (PMC3569660; doi:10.1002/emmm.201201378)
Supplement: Supplementary file 2 [file emmm0005-0149-SD2.pdf]

Targeting Aurora Kinases Limits Tumor Growth through DNA Damage Mediated Senescence and Blockade of NF-κB Impairs this Drug Induced Senescence

Supporting information

Figure S1. Human melanoma cell lines overexpress AURKA and AURKB and MLN8237 inhibits the phosphorylation of AURKA and AURKB. Page 1

Figure S2 and S3. MLN8054 and MLN8237 inhibit human melanoma growth *in vivo*. Page 2-3

Figure S4. The quantitative analysis of the results for p-AURKA staining on all patient tumors receiving vehicle control or MLN8237/MLN8054 treatment. Page 4

Figure S5. The H&E staining of vehicle or MLN8054/MLN8237 treated tumors. Page 5

Figure S6. The Ki67 staining of vehicle or MLN8054 treated tumors. Page 6

Figure S7. The histological features of relapsed tumor were analyzed by H&E staining when treatment was suspended. Page 7-8

Figure S8. The histological features of relapsed tumors after a second round of treatment with MLN8054 were analyzed by H&E staining. Page 9

Figure S9. TUNEL staining of vehicle control, MLN8237 or positive control treated tumor tissues (TMA). Page 10

Figure S10. SA-β-Gal staining of vehicle control or MLN8237 treated melanoma cells. Page 11

Figure S11. The levels of IL-6 and IL-8 in the supernatant of MLN8237-treated cells by ELISA. Page 12

Figure S12. The staining of DNA damage marker 53BP1 by immunofluorescence. Page 13

Figure S13. SA-β-Gal staining of vehicle control or MLN8237 treated patient tumor xenograft V29. Page 14

Figure S14. IKKβ stable knockdown impairs MLN8237-induced senescence. Page 15

Table S1. Spontaneous tumor formation in 12 month old FVB mice treated with MLN8237. Page 16

Table S2. The map of human cytokine antibody array 1. Page 17

Table S3. The map of human cytokine antibody array 2. Page 18

Fig S1

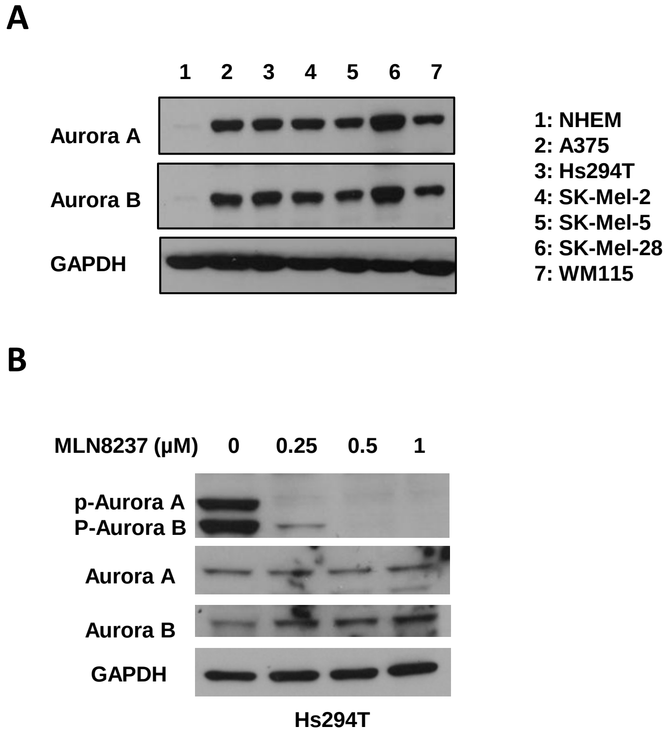

Figure S1. Human melanoma cell lines overexpress AURKA and AURKB and MLN8237 inhibits the phosphorylation of AURKA and AURKB. **A**. Human melanoma cells (A375, Hs294T, SK-Mel-2, SK-Mel-5, SK-Mel-28, and WM115) and normal human epidermal melanocytes (NHEM) were synchronized by 100 ng/ml of nocodazole for 16 hours followed by mitotic shake-off. The levels of Aurora A and and Aurora B were analyzed by western blot. **B.** Hs294T cells were treated with different concentrations of MLN8237 for 3 days. After treatment, the levels of p-Aurora A, p-Aurora B, Aurora A and Aurora B were determined by western blot.

Fig S2

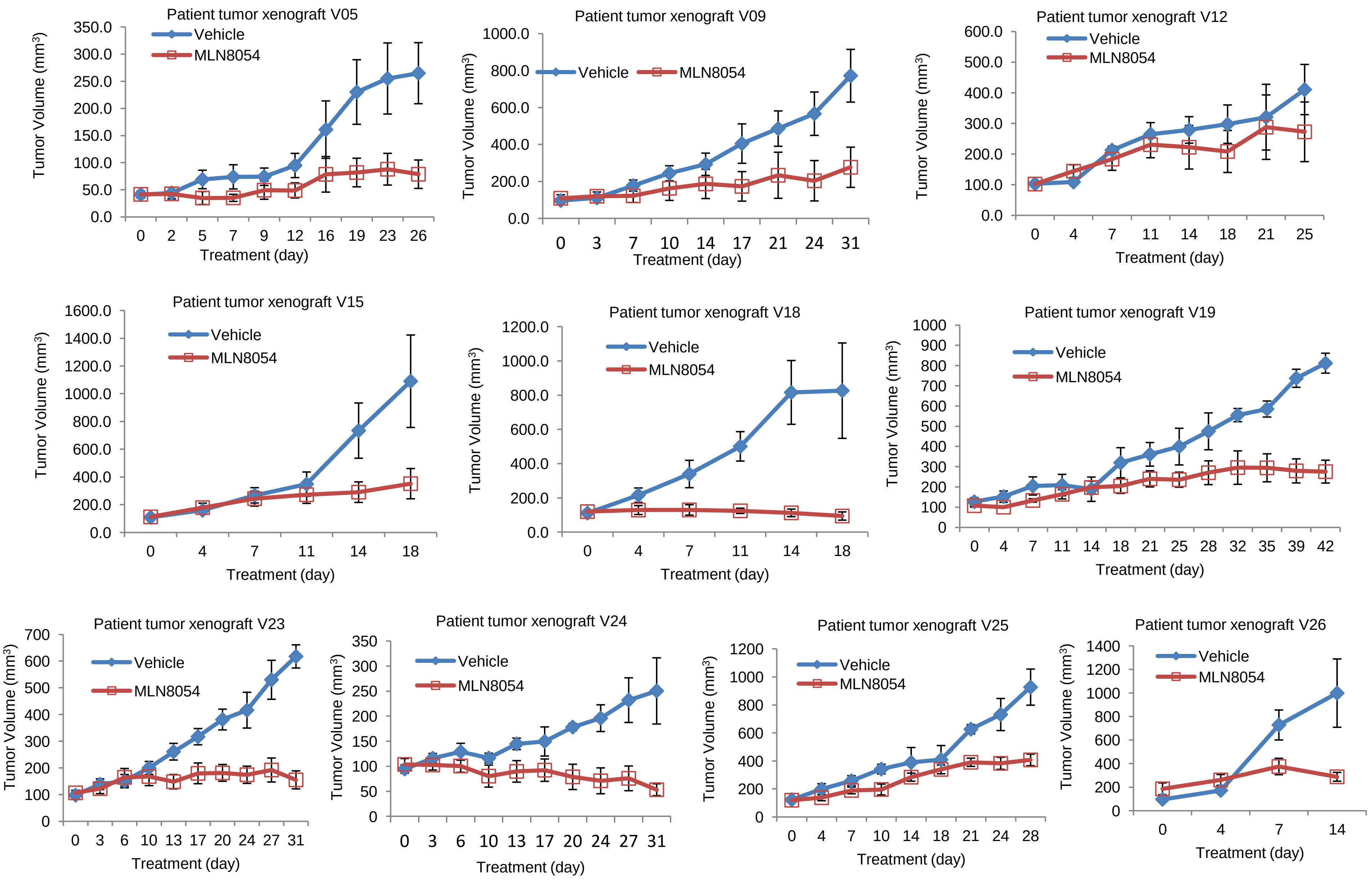
Patient tumor xenograft V05
Vehicle
MLN8054
Tumor Volume (mm³)
Treatment (day)
Patient tumor xenograft V09
Vehicle
MLN8054
Tumor Volume (mm³)
Treatment (day)
Patient tumor xenograft V12
Vehicle
MLN8054
Tumor Volume (mm³)
Treatment (day)
Patient tumor xenograft V15
Vehicle
MLN8054
Tumor Volume (mm³)
Treatment (day)
Patient tumor xenograft V18
Vehicle
MLN8054
Tumor Volume (mm³)
Treatment (day)
Patient tumor xenograft V19
Vehicle
MLN8054
Tumor Volume (mm³)
Treatment (day)
Patient tumor xenograft V23
Vehicle
MLN8054
Tumor Volume (mm³)
Treatment (day)
Patient tumor xenograft V24
Vehicle
MLN8054
Tumor Volume (mm³)
Treatment (day)
Patient tumor xenograft V25
Vehicle
MLN8054
Tumor Volume (mm³)
Treatment (day)
Patient tumor xenograft V26
Vehicle
MLN8054
Tumor Volume (mm³)
Treatment (day)

Fig S3

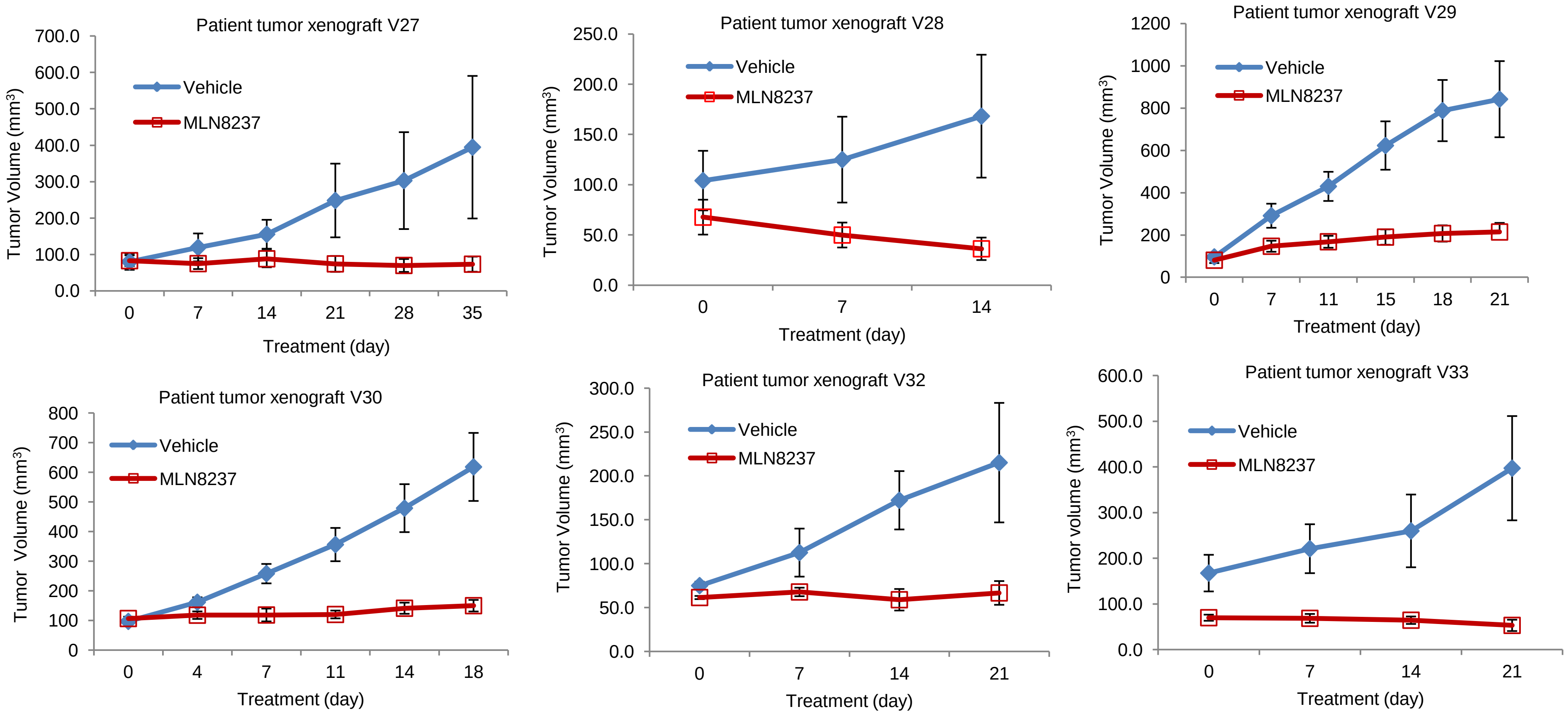

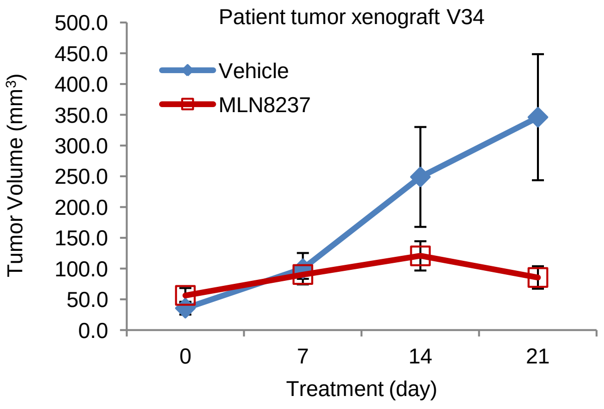

Figure S2 and S3. MLN8054 and MLN8237 inhibit human melanoma growth *in vivo*. Biopsy tumor tissues from melanoma patients were implanted into nude mice (n≥4 mice per group). When tumors were palpable, tumor-bearing mice received MLN8054 (60 mg/kg) (S2) or MLN8237 (30 mg/kg) (S3) by oral gavage for 2-6 weeks depending on their response to the treatment or the tumor volume.

Fig S4

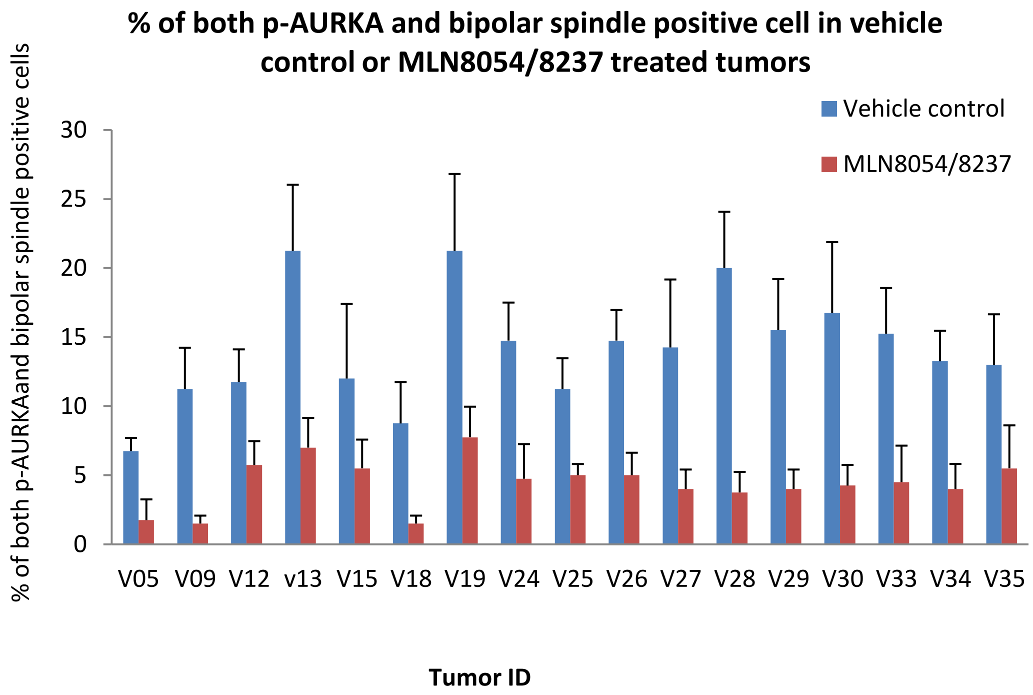

Figure S4. TMAs from patient tumor xenografts treated with MLN8054/MLN8237 or vehicle were examined for phosphorylated AURKA and bipolar spindle formation as measured by tubulin staining. Data are presented as percentage of positive cells.

Fig S5

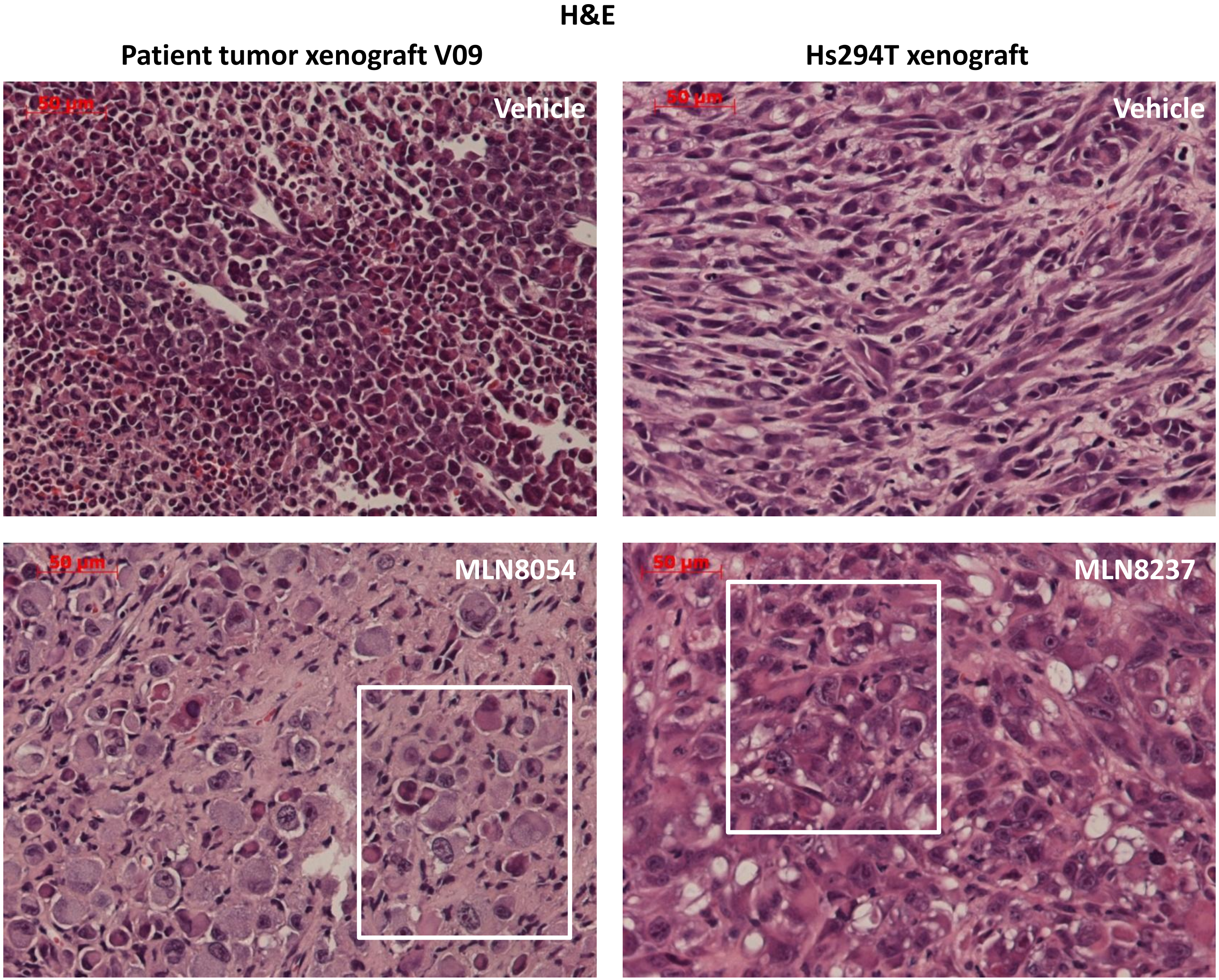

Figure S5. The H&E staining of vehicle or MLN8054/MLN8237 treated tumors. The histological features of patient tumor xenograft V09 or Hs294T xenograft treated with vehicle or MLN8054/MLN8237 were analyzed by H&E staining.

Fig S6

Patient tumor xenograft V19

Ki67

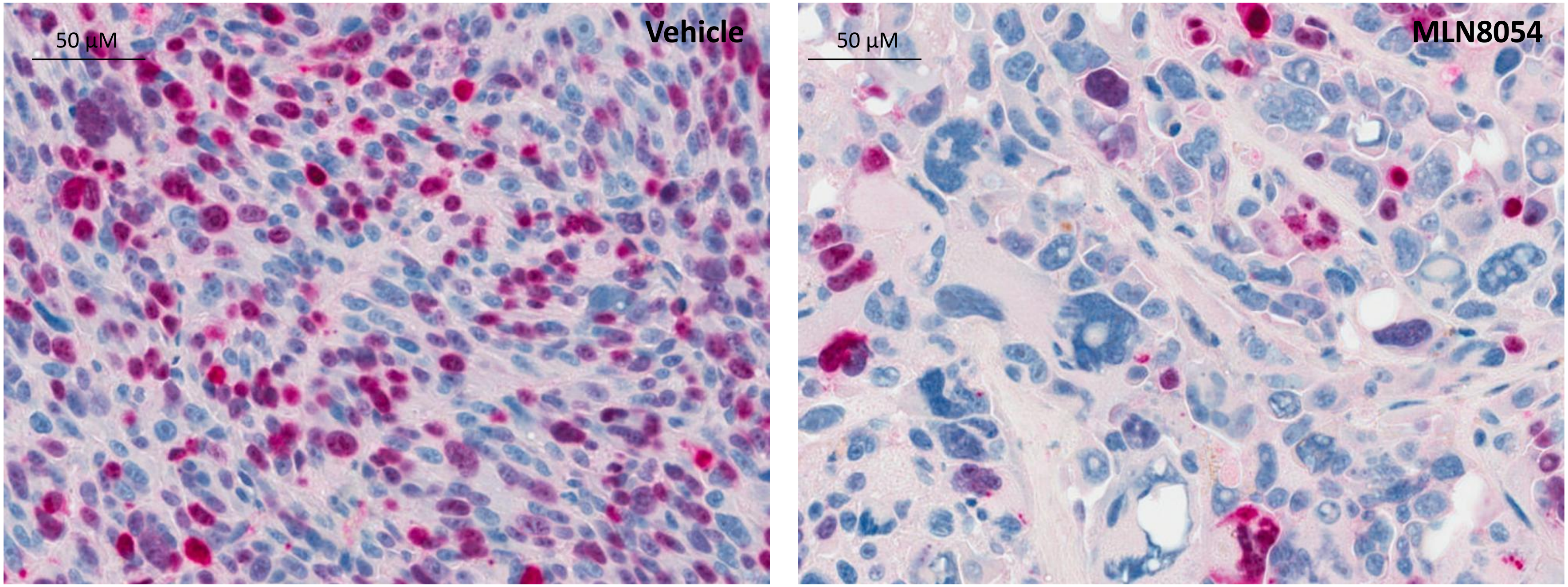

Figure S6. The proliferation of patient tumor xenograft V19 treated with vehicle or MLN8054 was determined by Ki67 staining.

Fig S7 A H&E Patient tumor xenograft V24

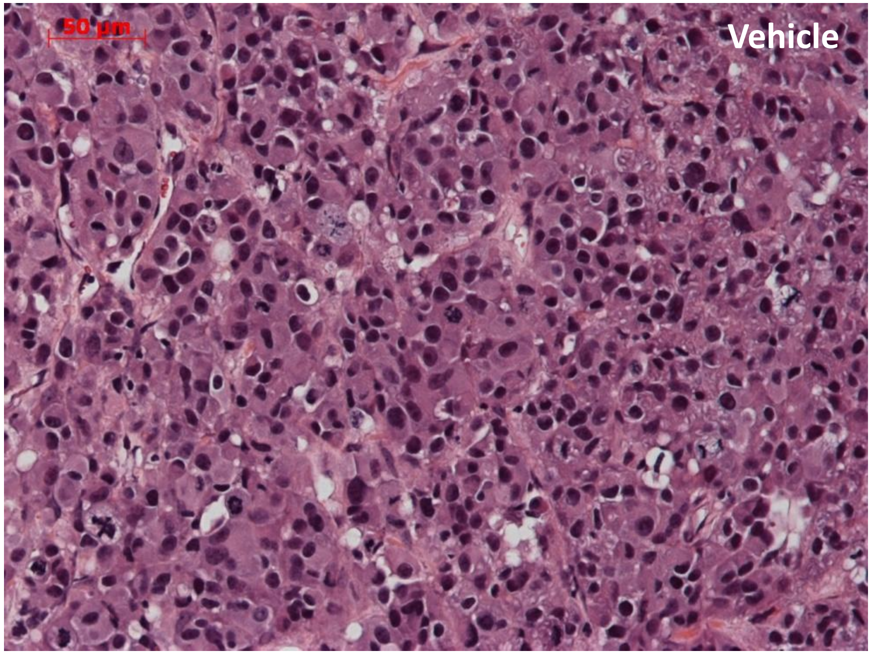

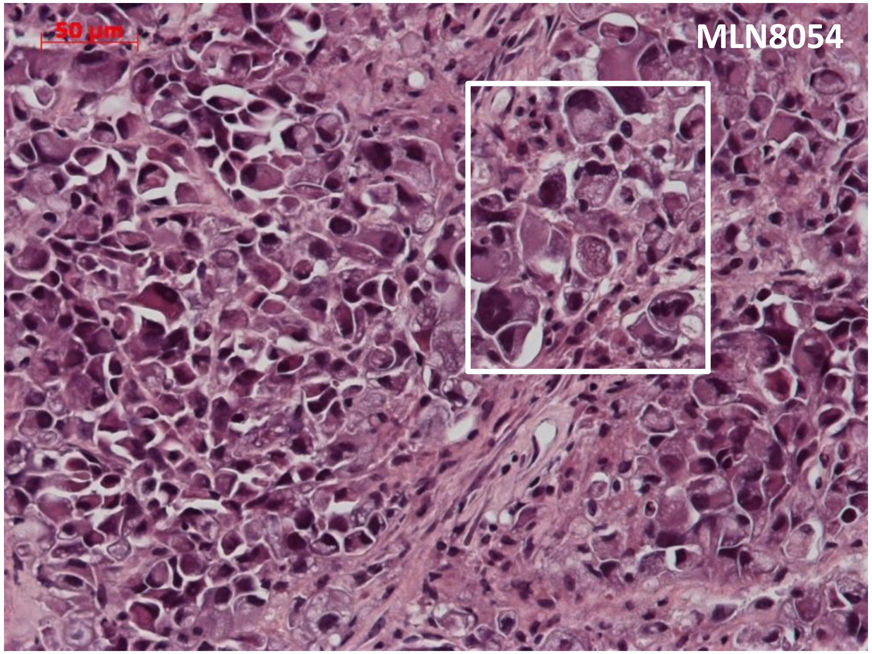

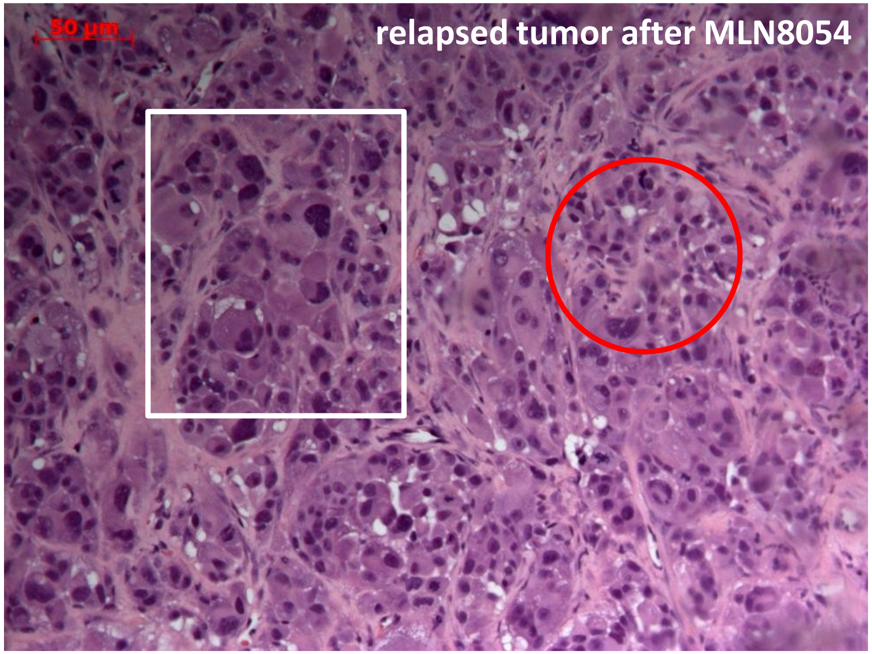

Figure S7. The histological features of relapsed tumor were analyzed by H&E staining when treatment was suspended. **(A)**: V24; **(B)**: V26
White boxes highlight regions of relapsed tumor displaying characteristics associated with senescence, such as the enlarged morphology seen in MLN8054 treated tumors. Red circles highlight regions displaying morphology consistent with vehicle treated tumors.

Fig S7 B H&E Patient tumor xenograft V26

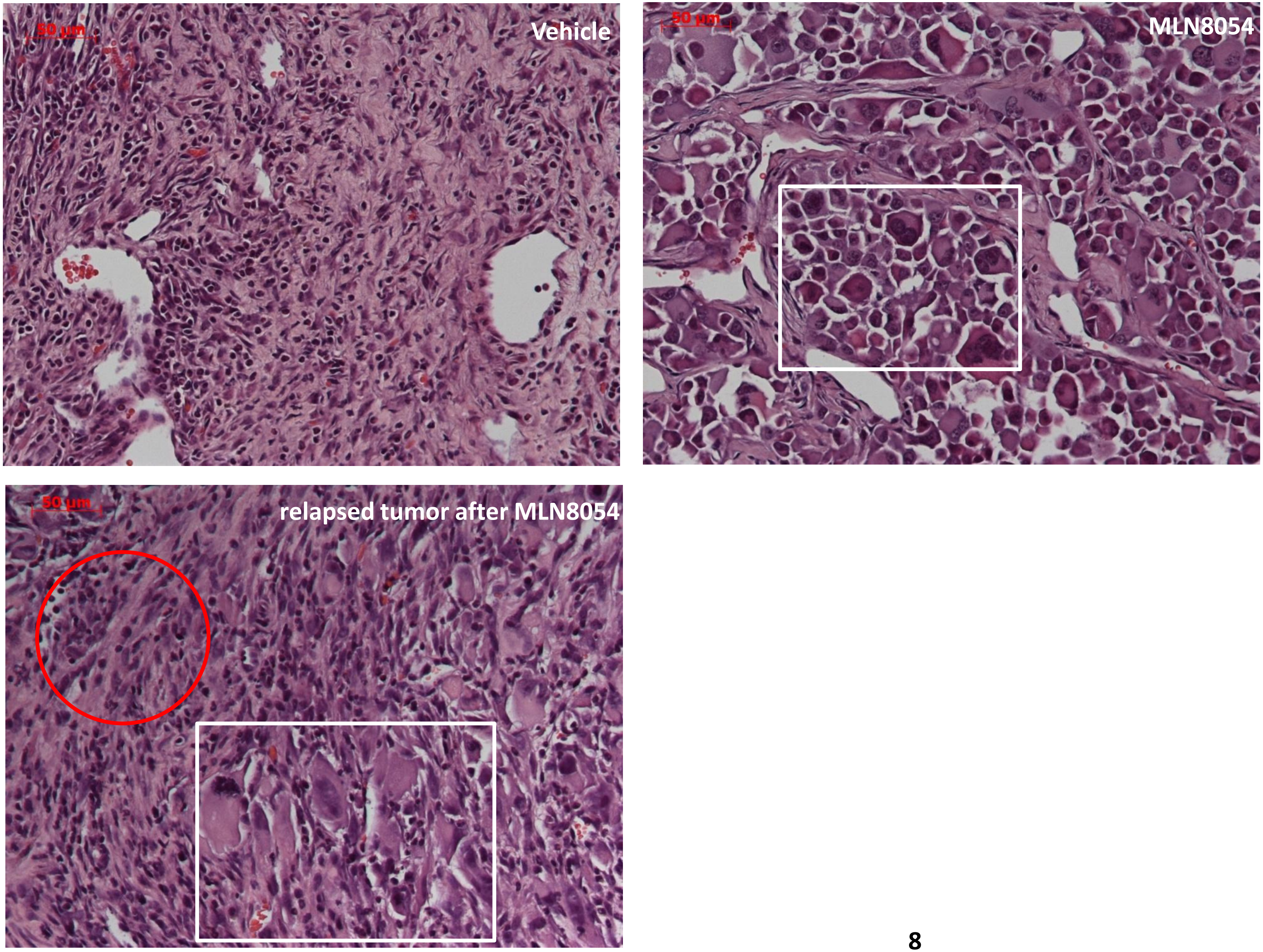

Fig S8

H&E

A

Patient tumor xenograft V23

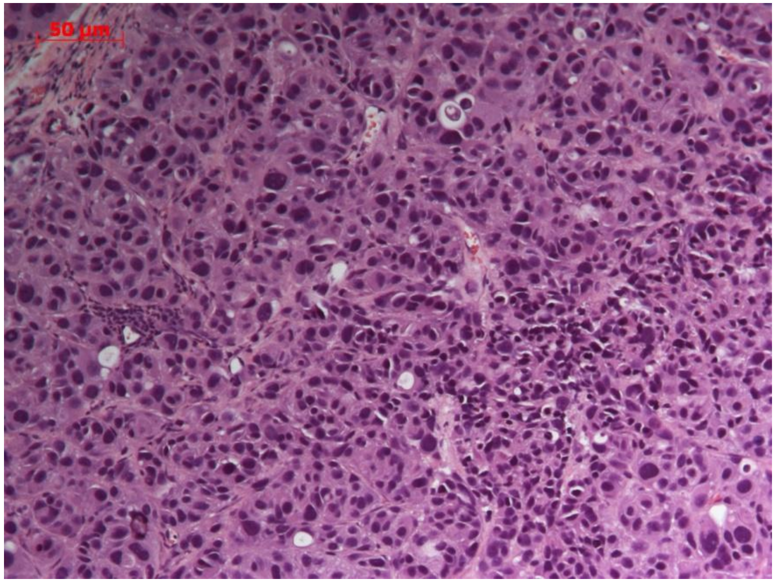

B

Patient tumor xenograft V26

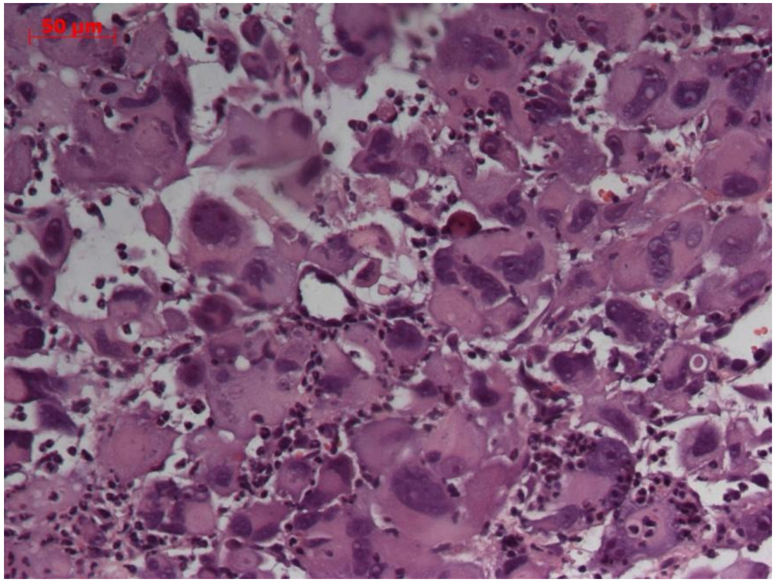

C

Patient tumor xenograft V24 (1)

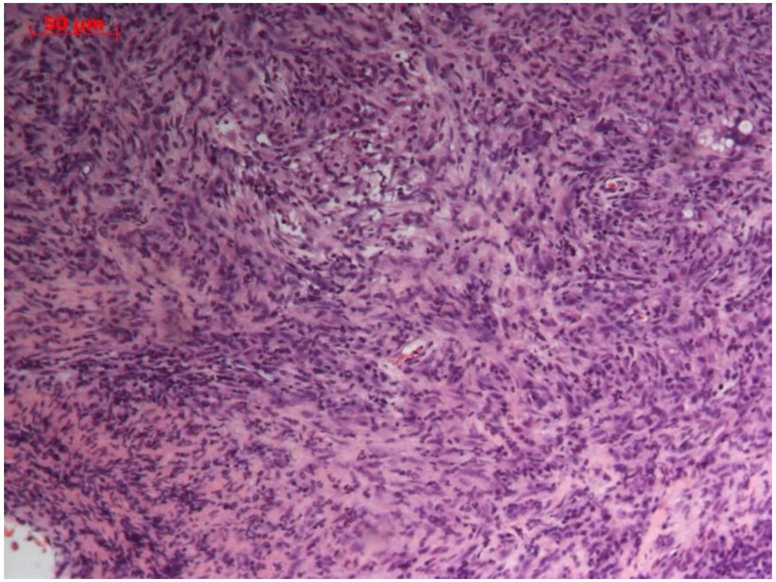

D

Patient tumor xenograft V24 (2)

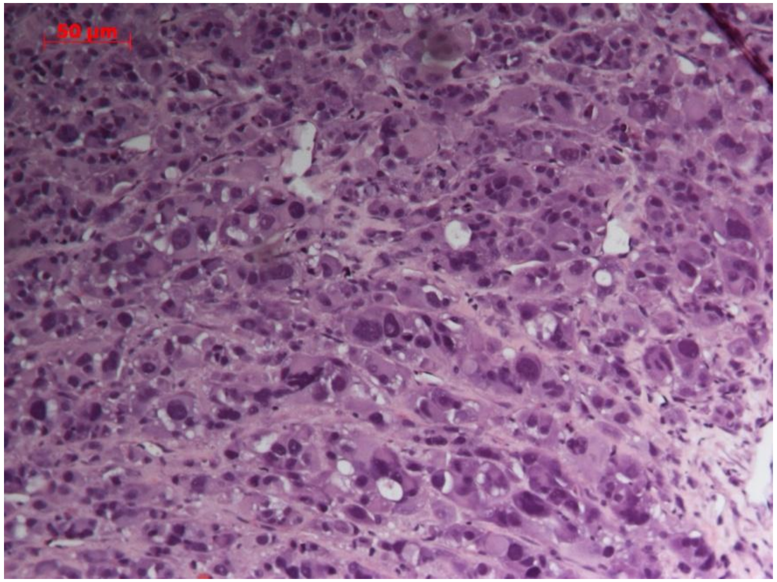

Figure S8. The histological features of relapsed tumors after a second round of treatment with MLN8054 were analyzed by H&E staining. Relapsed tumor-bearing mice were treated with MLN8054 for 2-3 weeks. **(A)** Non-responding tumor xenograft V23 **(B)** Responding tumor xenograft V26 **(C)** Non-responding tumor xenograft V24 **(D)** Responding tumor xenograft V24

Fig S9

Patient tumor xenograft V35

TUNEL staining

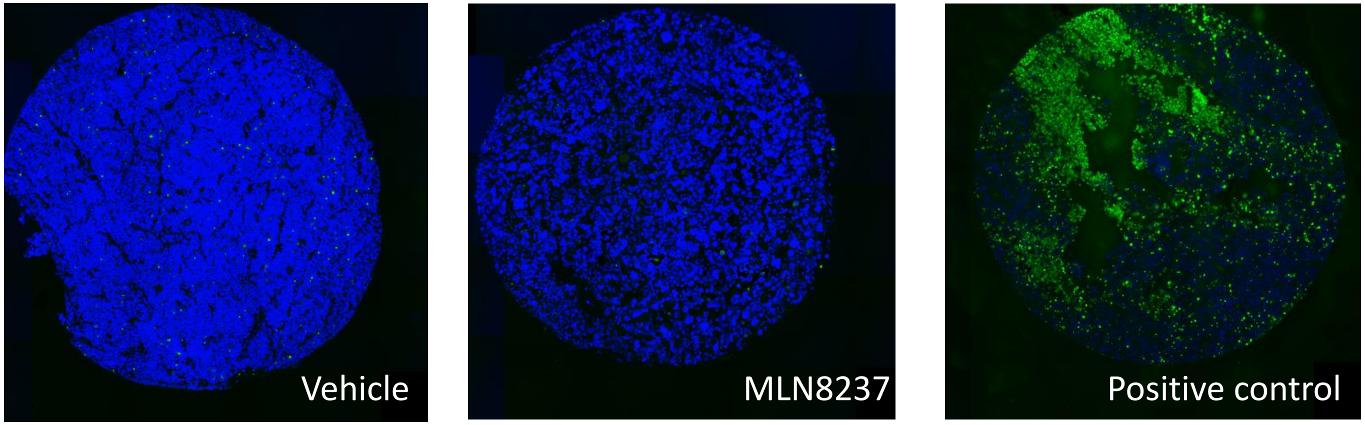

Figure S9. TUNEL staining of vehicle control, MLN8237 or positive control treated tumor tissues (TMA). Apoptosis in MLN8237 treated patient tumor implant was determined by TUNEL staining.

Fig S10

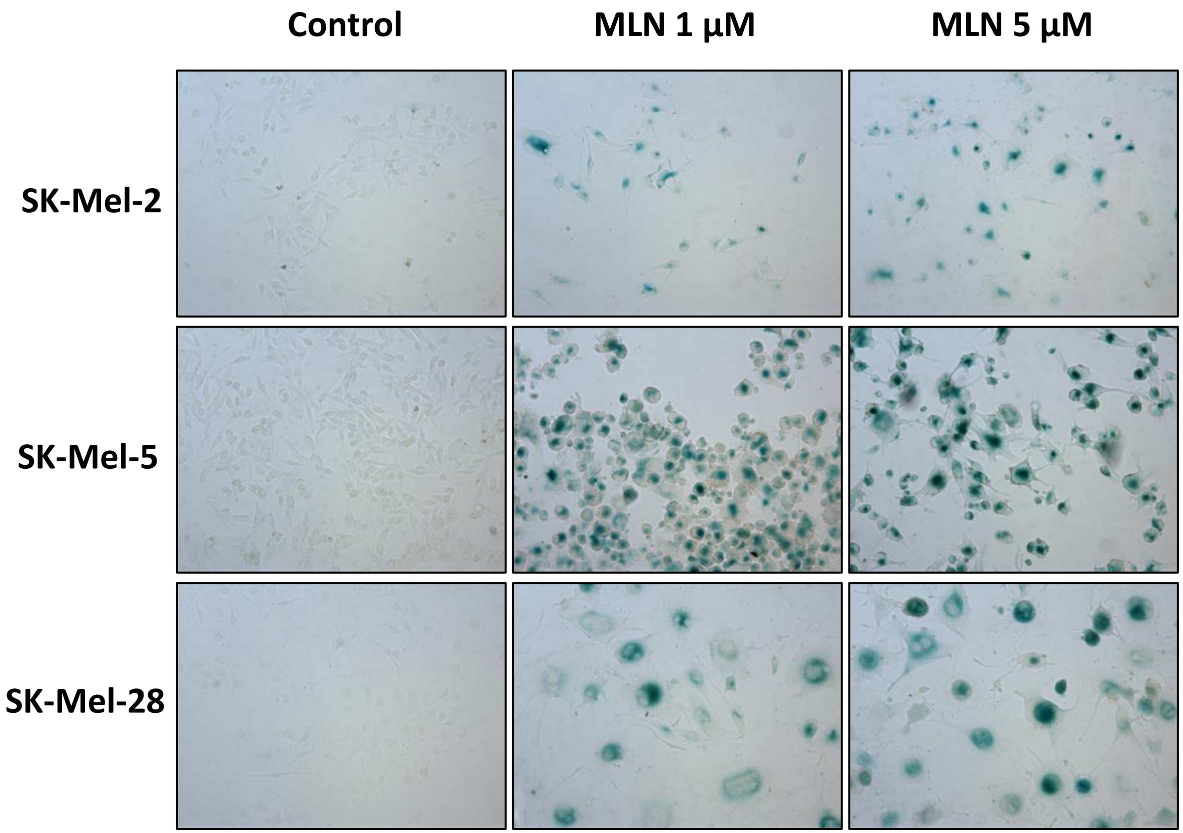

Figure S10. SA-β-Gal staining of vehicle control or MLN8237 treated melanoma cells. Melanoma cells were treated with MLN8237 for 5 days. After treatment, cells were fixed and stained with β-Gal.

Fig S11

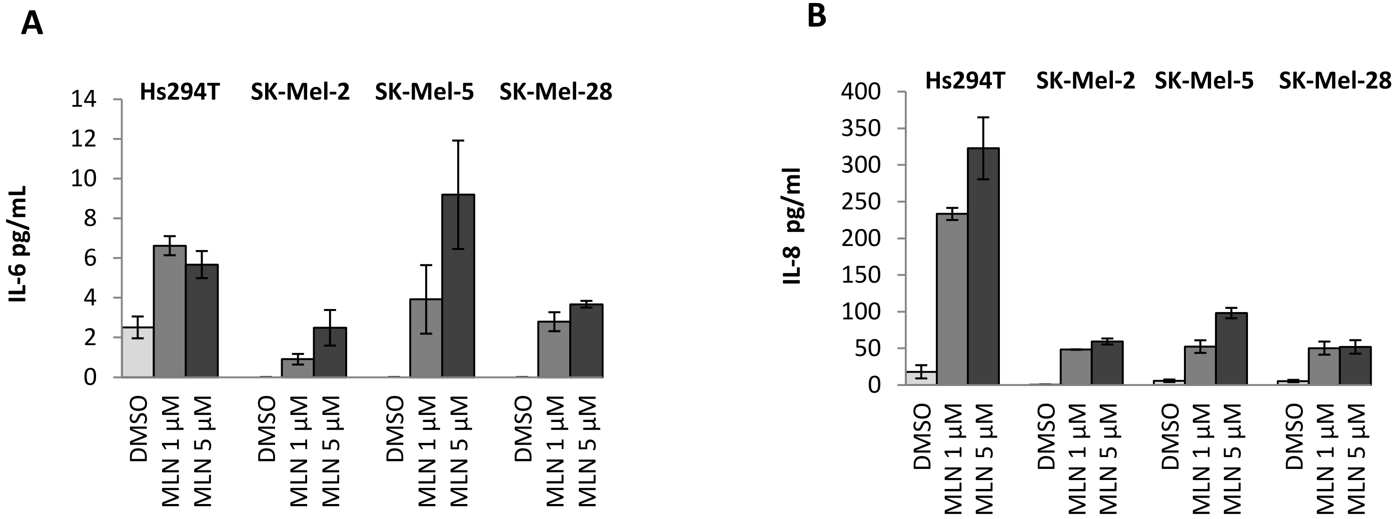

Figure S11. The levels of IL-6 and IL-8 in the supernatant of MLN8237-treated cells by ELISA. 10^4 vehicle or MLN8237 treated cells were seeded in a 24-well plate. After cells attached to the plate, serum free media were replaced and cells were incubated overnight. 100 µl of supernatant was measured for IL-6 or IL-8 by ELISA.

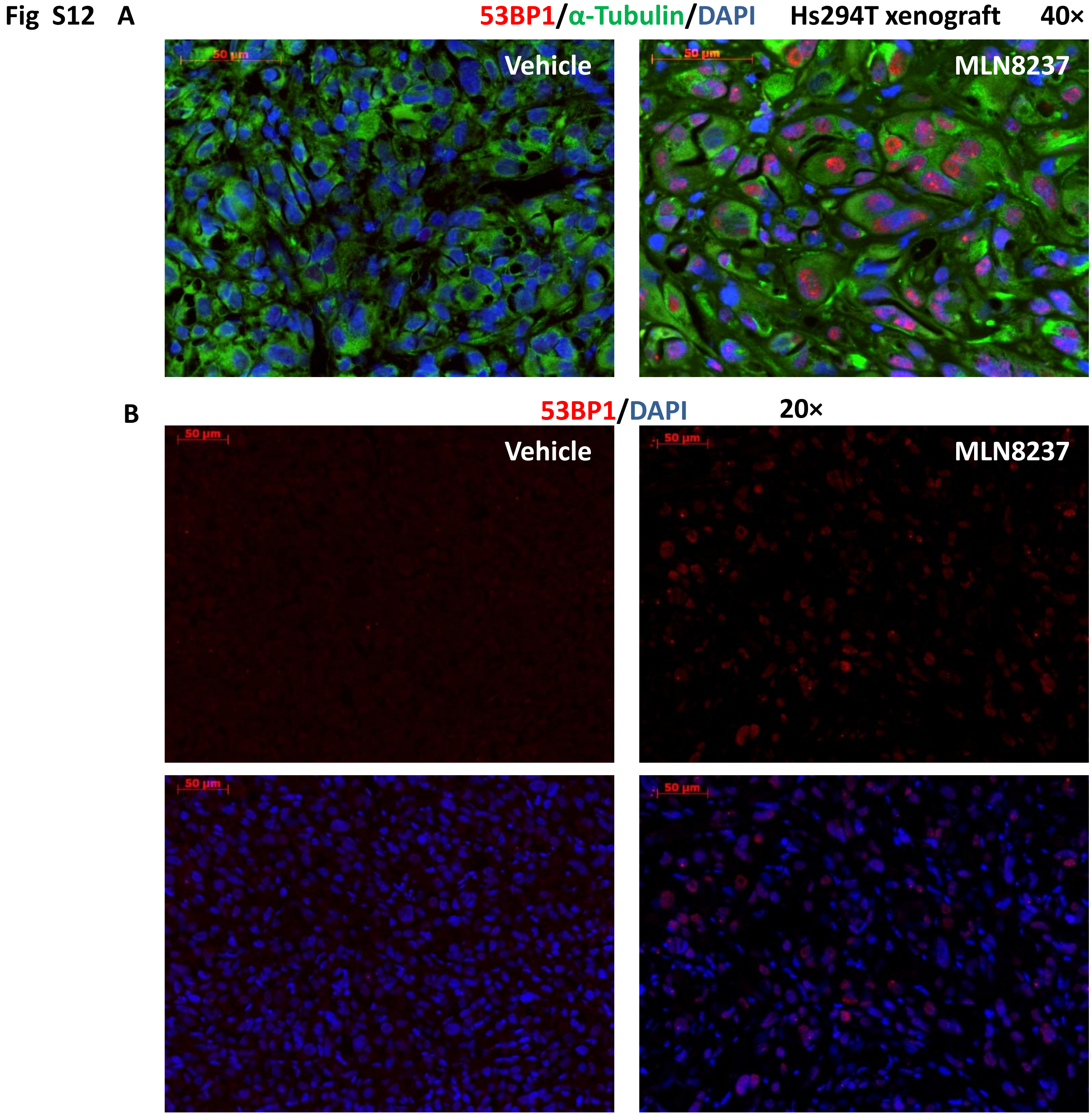

Figure S12. The staining of DNA damage marker 53BP1 by immunofluorescence. Full images of Figure 5B are shown here. (A) and (B) are from Hs294T xenograft tumors from different tumor-bearing mice. (A) Tumor tissue was stained for 53BP1, α-Tubulin, and DAPI. Images were captured at 40x magnification. (B) Tumor tissue was stained for 53BP1 and DAPI. Images were captured at 20x magnification.

Fig S13

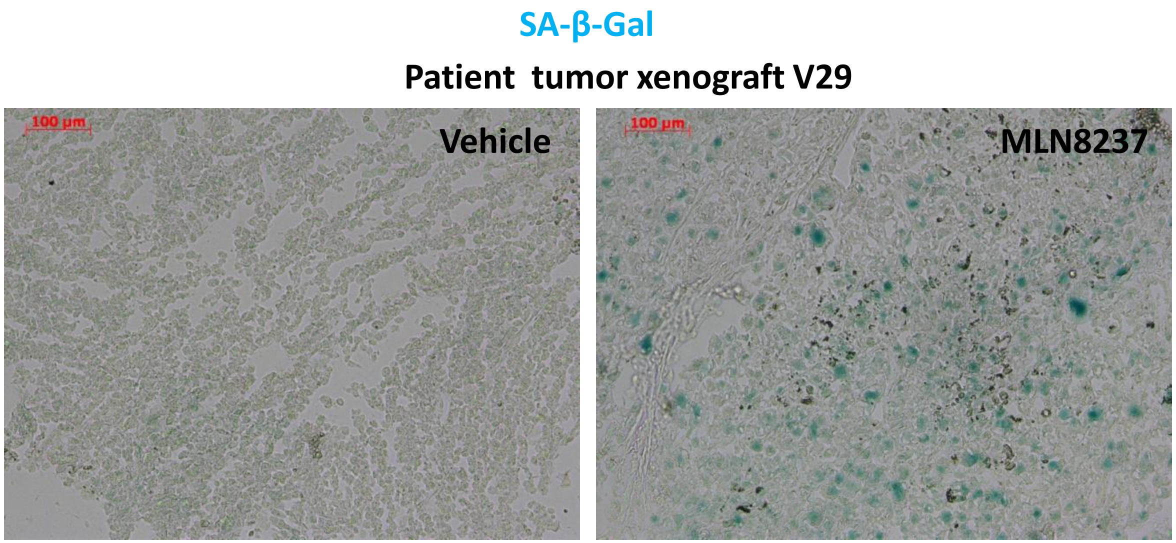

Figure S13. Patient tumor xenograft V29 treated with MLN8237 or vehicle was fixed and stained with β-Gal (blue).

Fig S14

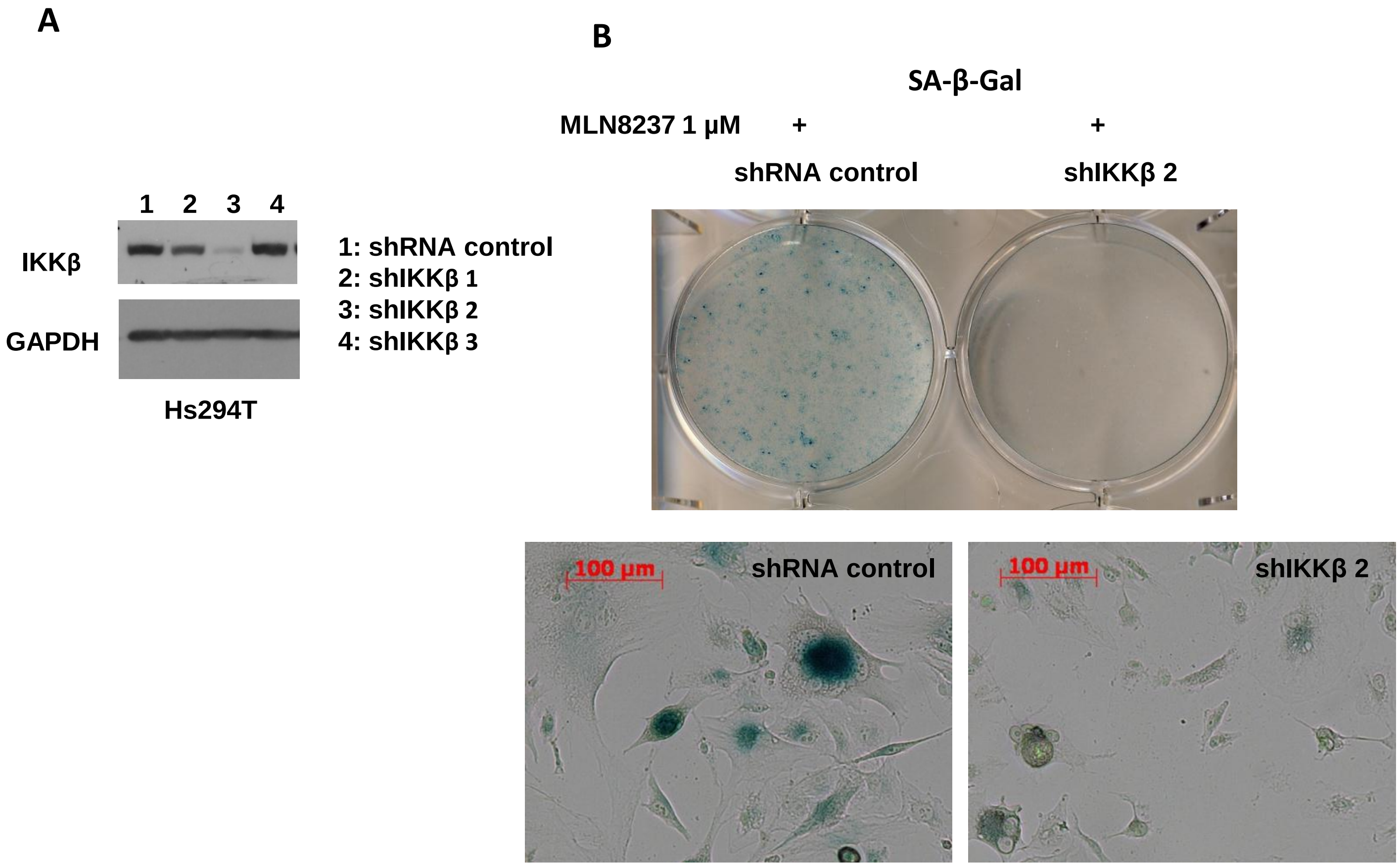

Figure S14. IKKβ stable knockdown impairs MLN8237-induced senescence. (A) IKKβ stable knockdown Hs294T cells were generated using shIKKβ and puromycin selection. (B) IKKβ knockdown and control cells were treated with MLN8237 for 5 days followed by β-Gal staining (blue).

Table S1. Spontaneous tumor formation in 12 month old FVB mice.

Treatment	Pathologic Diagnosis	Lung	Liver	Kidney	Spleen	Mammary Fat Pad	Small Intestine	Colon
MLN8237	Tumor	2/22	0/22	0/22	0/22	0/22	0/22	0/22
	Hyperplasia	0/22	3/22	0/22	0/22	0/22	0/22	1/22
Vehicle	Tumor	0/16	0/16	0/16	0/16	0/16	0/16	0/16
	Hyperplasia	0/16	1/16	0/16	0/16	0/16	0/16	0/16

Note: FVB retired breeders were aged to 12 months of age before receiving 40 mg/kg MLN8237 qd for 4 months. Tissues were collected and paraffin embedded. Five micron sections were stained with hematoxylin and eosin. Stained tissues were examined for tumor by a pathologist blind to the study groups.

Table S2. Human Cytokine Antibody Array 1

Positive	Positive	Negative	Negative	GCSF	GM-CSF	GRO	GRO-α
Positive	Positive	Negative	Negative	GCSF	GM-CSF	GRO	GRO-α
IL-1α	IL-2	IL-3	IL-5	IL-6	IL-7	IL-8	IL-10
IL-1α	IL-2	IL-3	IL-5	IL-6	IL-7	IL-8	IL-10
IL-13	IL-15	IFN-γ	MCP-1	MCP-2	MCP-3	MIG	RANTES
IL-13	IL-15	IFN-γ	MCP-1	MCP-2	MCP-3	MIG	RANTES
TGF-β1	TNF-α	TNF-β	Blank	Blank	Blank	Blank	Positive
TGF-β1	TNF-α	TNF-β	Blank	Blank	Blank	Blank	Positive

Table S3. Human Cytokine Antibody Array 2

Positive	Positive	Negative	Negative	ENA-78	GCSF	GM-CSF	GRO	GRO-α	I-309	IL-1α	IL-1β
Positive	Positive	Negative	Negative	ENA-79	GCSF	GM-CSF	GRO	GRO-α	I-310	IL-1α	IL-1β
IL-2	IL-3	IL-4	IL-5	IL-6	IL-7	IL-8	IL-10	IL-12 p40p70	IL-13	IL-15	IFN-γ
IL-2	IL-3	IL-4	IL-5	IL-6	IL-7	IL-8	IL-10	IL-12 p40p70	IL-13	IL-15	IFN-γ
MCP-1	MCP-2	MCP-3	MCSF	MDC	MIG	MIP-1δ	RANTES	SCF	SDF-1TA	RC	TGF-β1
MCP-1	MCP-2	MCP-3	MCSF	MDC	MIG	MIP-1δ	RANTES	SCF	SDF-1TA	RC	TGF-β1
TNF-α	TNF-β	EGF	IGF-1	Angiogenin	Oncostatin M	Thrombopoietin	VEGF	PDGF BB	Leptin	Negative	Positive
TNF-α	TNF-β	EGF	IGF-1	Angiogenin	Oncostatin M	Thrombopoietin	VEGF	PDGF BB	Leptin	Negative	Positive
